# Emergence of binding and catalysis from a designed generalist binding protein

**DOI:** 10.1101/2025.01.30.635804

**Published:** 2025-01-31

**Authors:** Yuda Chen, Sagar Bhattacharya, Lena Bergmann, Galen J. Correy, Sophia Tan, Kaipeng Hou, Justin Biel, Lei Lu, Ian Bakanas, Nicholas F. Polizzi, James S. Fraser, William F. DeGrado

**Affiliations:** 1 Department of Pharmaceutical Chemistry & Cardiovascular Research Institute, University of California, San Francisco, CA 94158, USA.; 2 Department of Bioengineering and Therapeutic Sciences, University of California, San Francisco, CA 94158, USA; 3 Department of Cancer Biology, Dana-Farber Cancer Institute, Boston, MA 02215, USA.; 4 Department of Biological Chemistry and Molecular Pharmacology, Harvard Medical School, Boston, MA 02215, USA.

## Abstract

The evolution of proteins that bind to small molecules and catalyze chemical transformations played a central role in the emergence of life. While natural proteins have finely tuned affinity for their primary ligands, they also often have weak affinities for other molecules. These interactions serve as starting points for the evolution of new specificities and functions. Inspired by this concept, we determined the ability of a simple *de novo* protein to bind a set of diverse small molecules (< 300 Da) by crystallographic fragment screening. We then used this information to design one variant that binds fluorogenic molecule and another that acts as a highly efficient Kemp eliminase enzyme. Collectively, our work illuminates how the evolution of novel protein functions can emerge from existing proteins.

The evolution of life leveraged the emergence of primordial proteins that could bind, utilize, and create new small molecule metabolites and signaling molecules. These proteins evolve on a landscape that is often portrayed pictorially by mapping sequence space into two dimensions (1–3). Here, we expand this concept to understand binding specificity, which involves both sequence (collapsing sequence space to only the Y-axis) and chemical diversity (representing chemical space by the X-axis, Fig. 1). Proteins that bind to and catalyze small molecules evolve along this sequence-chemical space landscape, as they acquire new functions. The ability to bind to new chemical entities is represented by horizontal moves in chemical space and enabled through alteration of their amino acid sequences, which are represented by vertical moves.

Such new specificities are thought to have primarily been acquired after gene duplication. In these scenarios, a gene encodes a protein with an existing activity (e.g., strongly binding molecule A) and an “off-target” promiscuous activity (e.g., weak-moderate binding affinity to molecule B) (4–6). The duplication event frees the two copies – which initially have “generalist” activities to specialize independently. Mutations to the amino acid sequence of one copy can increase the specificity for B by substituting residues that contact B or that increase the population of a conformational sub-state that promotes binding. Thus, cells have been evolving an increasing array of proteins with distinct specificities that complement increasing functional complexity and adaptability.

Even within the gene duplication paradigm, searching broadly and simultaneously through both chemical and sequence space to evolve new functions is daunting. Most experimental work has focused on exploring each of these two variables in isolation: drug discovery involves an extensive search of chemical space to discover small molecules that bind to a target protein with a fixed sequence (7), while methods such as yeast display, phage display or directed evolution explore sequence space to discover new protein sequences for a given small molecule (2, 3). *De novo* protein design provides an alternative approach to explore sequence space, and, although *de novo* design of small molecule-binding proteins is still considered a significant challenge (8), a number of proteins have been designed to bind drugs and cofactors (8–15). However, it is unknown whether designed proteins are more or less specific than natural proteins. The great conformational stability of *de novo* proteins (14) and the idealized nature of their designed interactions might increase their specificity, making it difficult for them to act as generalists and to evolve new functions. Alternatively, they might have relatively low specificity because their binding sites have not been subject to the negative selective pressures that enforce specificity in a natural context.

Aside from acting on their primary substrates and closely related analogs, natural proteins have a low affinity (*K*_D_ in the low mM range) for a plethora of small molecular fragments (MW < 300 Da). Although these molecules can bear little resemblance to the protein’s natural ligands, they present starting points for evolution of new specificities (1, 5, 16). In this thinking, the active sites of natural proteins have a repertoire of weak binding sites, which stand ready to evolve novel functions (5). Here, we probe the implications of this idea by using fragment discovery to design new binding and catalytic activity paired with *de novo* sequence design.

We chose helical bundles for this application, because they represent one of the most ancient, evolvable, and arguably the most functionally diverse tertiary structures (17). Most transmembrane proteins are helical bundles (with the notable exception of beta barrels in outer membranes of bacteria and mitochondria) and they are involved in transport, signal transduction, small molecule binding, and catalysis of a wide range of reactions from proteolysis to the generation and utilization of O_2_ (18). For instance, G-coupled protein receptors (GPCRs) specialize to recognize a wide array of small molecules in sites bounded by four transmembrane helices (19). Helical bundles are also found in water soluble proteins involved in electron transport (20), metalloenzymes (21), small molecule binding and transport (e.g., serum albumin (22)), and natural enzymes such as chorismate mutase (23). *De novo* helical bundles have also been evolved to perform essential functions *in vivo* (24, 25).

As a starting point to explore the evolution of novel binding and catalytic functions from promiscuous binding, we used a *de novo* protein, the Apixaban-Binding helical BundLE (ABLE), which was previously designed to bind the antithrombotic drug, apixaban (Fig. 1) (13). While ABLE can discriminate apixaban from close congeners, we addressed its specificity towards more distantly related molecules using X-ray crystallography. This crystallographic method is one of several experimental and computational methods that is frequently used to discover fragments with very weak affinity but geometrically well-defined poses for structure-based drug design (26). We show that the malleability and promiscuity of ABLE is within the range seen in natural proteins studied by fragment screening (26–28). Conformational rearrangements of ABLE’s binding site were primarily restricted to sidechain rotamer shifts. Many of the ABLE-binding fragments showed little similarity to apixaban, and some bound in a novel aromatic box opened by conformational rearrangement of a Tyr sidechain. We next asked whether ABLE might be converted from a generalist for binding multiple fragments to a specialist for the binding of turn-on fluorophores. Using different cues from fragment complexes, we also converted ABLE to an enzymatic catalyst for the Kemp Elimination reaction (29–37) with a catalytic efficiency (*k*_cat_/*K*_M_ = 600,000 M^−1^s^−1^) exceeding the rates of mechanistically similar natural enzymes such as ketosteroid isomerase (*k*_cat_/*K*_M_ = 540,000 M^−1^s^−1^) (38) and triosephosphate isomerase (*k*_*cat*_/*K*_*M*_ = 440,000 M^−1^s^−1^) (39). Together with previous studies (13, 14, 40–46), our results show that an impressive range of catalytic and binding functions can emerge through sequence variation of even a simple four-helix scaffold similar to ones that might have emerged early in evolution. These findings have fundamental implications inspired by protein evolution, as well as practical implications for the design of (13, 47–51) new detectors and catalysts.

## Plasticity of the binding site from analyses of crystal structures and molecular dynamics (MD) simulations.

**Fig. 2.**
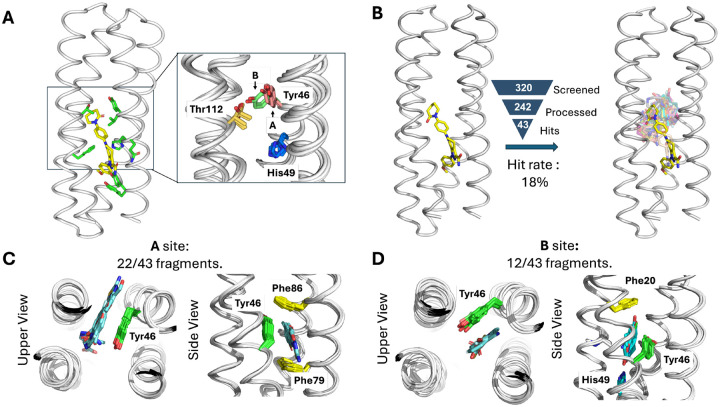
ABLE binds multiple fragments with small alterations of the binding sites. (**A**) Binding pockets in the apixaban-bound ABLE (PDB: 6W70) and the ligand-free forms (PDBs: 6W6X and 6X8N). The crystal structures have multiple monomers in the asymmetric unit, each of which was superimposed. (**B**) Crystallographic fragment screening reveals expanded chemical space of weak binders; the crystal structures of ABLE in complex with 43 fragments were each solved to to between 1.3–1.6 Å resolution, using Pan-Dataset Density Analysis. The structures were superposed onto the structure of the ABLE-apixaban complex. The backbone structure shown is from chain A of PDB: 6W70, the carbon atoms from the structure of apixaban are shown in yellow sticks, and the remaining residues’ carbon atoms in a different color for different fragments. (**C-D**) Most fragments interact with ABLE at one of two binding sites, designated **A** and **B**, which differ by a conformation shift of Tyr46. The complexes are shown looking down the axis of the bundle (left) and rotated by approximately 90° (right). **(C)** 22/43 fragments bind ABLE in an aromatic box formed by Tyr46, Phe79, Phe86, and the backbone of one helix (site **A**). **(D)** 12/43 fragments interact with ABLE at site **B**, where Tyr 46 adopts a different rotamer than in the ABLE-apixaban complex. Site **B** is formed by Phe20, Tyr46, and the backbone of two helices. His49 sits at the bottom of the pocket, forming hydrogen bonds with polar atoms of the fragments. The presence of the fragments in the binding site had only a small effect on the overall tertiary structure of the protein (sub-Å Cα RMSD).

To assess the potential of ABLE (Fig. 2A) to bind ligands other than apixaban, we first examined multiple crystallographic structures of the protein in the absence of apixaban to identify multi-conformational regions that might bind other small molecules. Although the binding site was largely pre-organized to the shape of apixaban (13), multiple sidechain conformations were observed near the upper portion of the site (Fig. 2A). In particular, Tyr46 adopted two conformations in the drug-free state, only one of which was present in the apixaban complex. We define the conformer seen in the apixaban complex as “**A**” (for apixaban) and the alternate sub-state as “**B**”. A similar conformation emerged from running multiple molecular dynamics simulations of the protein (see section 1.9.1–1.9.2 of the SI). In simulations, Tyr46 exchanged between both sidechain conformations in the drug-free state (fig. S1). Although there is a relatively modest difference in the chi1 and chi2 angles between the two conformers, this small change propagates to a larger displacement of its long aryl sidechain due to the lever arm effect. Other sources of conformational variability in this region included the sidechains of His49, which also polulates more than one rotamer (fig. S2). Collectively, this analysis suggests that the apixaban binding site displays limited sidechain flexibility, particularly at Tyr46 and His49, in the apo state.

## Conformational plasticity and binding specificity of ABLE from crystallographic fragment screening

We next used X-ray crystallography to map regions of ABLE’s site that were capable of binding to a diverse set of “fragment” (MW < 300 Da) molecules (52), which span a range of aliphatic and aromatic character, a logP value between −2.3 and 2.6, and up to four heteroatoms. (see section 1.1–1.2 of the SI). We screened a modest-sized library of 320 small fragments, which represent drug-like moieties, for their ability to bind to ABLE when soaked for two hours into ABLE crystals at about 10 mM concentration (Fig. 2B). Of the 242 compounds where high-quality X-ray diffraction datasets were collected, 43 unique fragments were modeled (Fig. 2B). The resulting hit rate (18%) is similar to the reported hit rates of 7% and 19% for Nsp3 macrodoamin of SARS-CoV-2 and Chikungunya virus, respectively, using the same fragment library and screening method (27, 28) (Fig. 2B). All but four fragments bound within the apixaban binding site. Thus, by this criterion, ABLE has a specificity within the range seen in natural proteins.

The 39 fragments that bound in ABLE’s apixaban-binding site represented diverse chemotypes. The hits collectively have a small but statistically significant tendency to be larger, more apolar, and richer in atoms that are part of aromatic rings when compared to the overall library. (fig. S3) While apixaban has a primary carboxamide (–CO–NH_2_) that is bound near the surface of the protein in the ABLE-drug complex. However, the fragments in the 39 complexes were bound more deeply into the pocket and none were found to contain this polar functional group.

Almost all of the bound fragments slot into one of two boxes formed by the distinct conformations of Tyr46 (figs. S4–5). About two-thirds of the fragments bound to conformer **A**, characterized by an aromatic box comprised of the sidechains of Tyr46 (conformer **A**), Phe79, Phe86, and the backbone of **helix 3**. (Fig. 2C) The other third bound to conformer **B**, which is also an aromatic box comprised of a distinct Tyr46 side chain conformation, Phe20, His 49, and the backbone of **helix 1** (Fig. 2D). The great majority of the fragments also form polar interactions with residues adjacent to the aromatic boxes: namely, Thr112 in conformer **A** and His49 in conformer **B**. (fig. S5) This malleability of ABLE enables adjustment to the shape and properties of the fragments by conformational selection, which is a property of generalist binders (1).

## Designing Proteins to Bind Fluorogenic Ligands

**Fig. 3.**
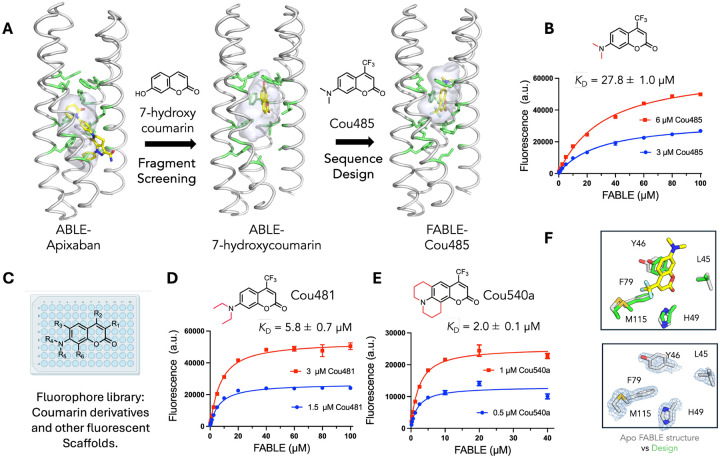
Design of a fluorescent ABLE (FABLE) binding to fluorogenic coumarins. (**A**) 7-hydroxycoumarin was found from crystallographic fragment screening to bind ABLE. The **B** box (Fig. 2D) was used to design five sequences for binding to Cou485. The positions of the apixaban-interacting residues (green sticks) and the binding cavity (grey surface) show how the size and location of a binding site change as the site transitions from binding apixaban, to a small coumarin, and then to a Cou485. **(B)** A titration of FABLE into Cou485 shows a single-site binding isotherm. The dissociation constant was obtained by globally fitting a single-site binding model to data obtained by titrating FABLE into Cou485 at two fixed fluorophore concentrations, 3 and 6 μM. **(C)** Returning to the exploration of chemical space of FABLE’s binding properties using a set of 37 fluorophores, including coumarin derivatives and other fluorophore scaffolds. Two additional FABLE-binding coumarin derivatives were identified. **(D-E)** The binding constants for FABLE’s interaction with Cou481 **(D**) and Cou540 **(E)** were obtained at the indicated fixed fluorophore concentrations and variable FABLE concentrations as in **B**. **(F)** Upper: Overlay of the designed FABLE-cou485 complex with the uncomplexed FABLE crystal structures (PDB: 9DWC, comparison with 9DWA and 9DWB are in fig. S10). Lower: 2mF_O_-DF_C_ electron density (1 σ) contoured around the active site residues of FABLE (PDB: 9DWC). Error bars in B, D and E represent standard deviations of three independent measrements.

We next asked whether the newly discovered site defined by conformer **B** would be a good starting point to design a protein with altered binding specificity. Site **B** of ABLE bound the fragment 7-hydroxycoumarin (Fig. 3A), which represents to core of a large class of fluorophores. This suggests that it would be possible to redesign ABLE to bind environment-sensitive, turn-on fluorophores that are larger than the starting fragments. We therefore redesigned the B site to accommodate the larger environment-sensitive coumarin, Cou485 (1, 7-(dimethylamino)-4-(trifluormethyl)-coumarin) using the 7-hydroxycoumarin complex as the starting structure (Fig. 3A) (53). The goal of the design was to: 1) create more space to accommodate the additional trifluoromethyl and dimethylamino groups of Cou485, and 2) fill in the pockets that had been used to bind apixaban but were no longer needed for binding Cou485. (Fig. 3A) Using a previously described protocol (44, 54), we selected 43 of ABLE’s 126 residues to vary during redesign. Fig. 3A illustrates the gradual migration of the binding site, which accompanied binding to the larger ligand. OmegaFold (55) was used to determine whether the new designs would be preorganized in a conformation capable of binding Cou485; other selection metrics are described in the supplement (see section 1.3 of SI).

Five of the designed proteins with sequence pairwise identity ranging from 77% to 81% were selected for experimental characterization.(fig. S6) All five were helical based on far-UV circular dichroism spectroscopy and thermostable to 90 °C (fig. S7) (see section 1.4 and 1.10 of SI). They also all bound the target as assessed by fluorescence spectroscopy. The intensity of the emission spectra of Cou485 increases markedly and shifts towards lower wavelengths when bound in a rigid, hydrophobic binding site (56). Indeed, the fluorescence of Cou485 (6 μM) increased by 10 to 99-fold in the presence of 40 μM of the five different proteins (fig. S8). Attesting to their specificity, the starting ABLE protein did not show signs of binding to Cou485 based on the same fluorescence assay (fig. S8). Global analysis of the titration curves obtained at two fixed Cou485 concentrations and variable protein concentrations indicated that all five proteins bound Cou485 with *K*_D_ < 100 μM, and three exhibited *K*_D_ ranging from 28 μM to 38 μM (fig. S7). The design showing the most potent *K*_D_ (27.8 ±1.0 μM) was designated as FABLE (Fluorescent ABLE) (Fig. 3B) (see section 1.7 of SI).

Ligand efficiency is an empirical metric used to assess the effectiveness of a designed protein’s interaction with a small molecule ligand (57, 58). Large ligands have more opportunities to interact favorably, so ligand efficiency roughly normalizes for the size of the molecule by dividing the free energy of binding (1 M standard state) by the number of heavy atoms in the ligand. Most FDA-approved small molecule drugs have ligand efficiency around 0.3 kcal/(mol • heavy atom count) (58). The ligand efficiencies for the five proteins with Cou485 range from 0.31 to 0.35 kcal/(mol • heavy atom count) (table S1). This suggests that the quality of the interactions in the binding site is in the range of what is expected for designed small molecule interactions. To test this idea and address the question of whether FABLE had a well-defined, preorganized structure, we determined crystallographic structures of ligand-free FABLE in three different crystal forms (1.5–1.9 Å resolution) (see section 1.8 of SI). Each of the structures was in excellent agreement (< 1 Å Cα RMSD) with the designed model and the AlphaFold3-predicted structure. (Fig. 3F, figs. S9–10 and table S2).

## Experimental exploration of the chemical space to probe the specificity of FABLE

Having explored sequence space to enhance specificity for Cou485 over the original apixaban, we next explored chemical space to evaluate specificity for related, larger, fluorophores. We evaluated 37 fluorophores that shared molecular features such as hydrophobicity and aromaticity (Fig. 3C and table S3) (see section 1.5 of SI). Of these compounds, only two showed a four-fold or greater increase in fluorescence intensity in the presence of FABLE, compared to their fluorescence in the presence of ABLE (fig. S11). The compounds with a large increase in fluorescence intensity were derivatives of cou485 with slightly larger apolar groups at the 7-amino position of the coumarin core (Fig. 3D–E). Fluorescence titrations showed that the dissociation constants for Cou481 and Cou540 were 5.8 ± 0.7 μM and 2.0 ± 0.1 μM, respectively (Figs. 3D and 3E), and stable by size exclusion chromatography (fig. S12) (see section 1.6 of SI). The corresponding ligand efficiencies are 0.35 and 0.34, similar to that of the original target Cou485 (0.34) (table S1). Thus, the increases in affinity for Cou481 and Cou580 were primarily driven by a larger interaction surface. The maximal fluorescence enhancement at saturating concentrations of FABLE was 48-fold for Cou485, 123-fold for Cou481, and 117-fold for Cou540 (fig. S10), suggesting that similar *de novo* proteins might be useful for future imaging and sensing applications (8, 59) (fig. S12). Collectively, these results present a path for evolving new binding specificity and how related compounds can leverage an initial broad, low-affinity binding even when they were not part of the selection pressure or design calculation.

## Design of an efficient Kemp eliminase

**Fig. 4.**
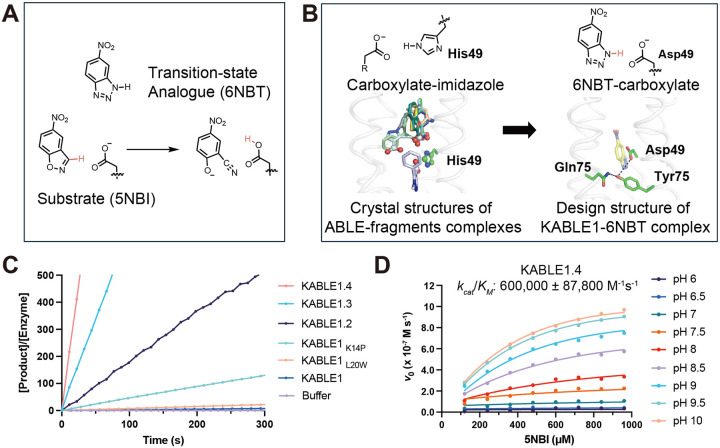
Design of an efficient Kemp eliminase ABLE (KABLE) and its directed evolution. (**A**) The Kemp elimination reaction. (**B**) Fragment-inspired design of KABLE. This carboxylate-to-imidazole interaction is analogous to the carboxylate-to-isoxazole interaction at the active site of a kemp eliminase, suggesting that an active catalyst might be designed by changing His49 into an Asp. Left: Nine of the fragments contain carboxylates that bind to His49. Right: The active site was introduced into the ABLE backbone by substituting the His49 sidechain with the most frequently observed rotamer of Asp (64). 6NBT (sticks with yellow carbons) was positioned in the saddle point geometry of the transition state for the carboxylate-catalyzed reaction as determined by quantum mechanical calculations (29). Sequence design was next carried out with LigandMPNN and Rosetta FastRelax (47, 54). The Asp triad (Asp49-Tyr9-Gln75) is shown in sticks. **(C)** Catalytic activity for the Kemp elimination reaction for the original sequence, designated KABLE1. Mutants arising from the combination of variants from site-saturation mutagenesis show a steady progression as more sites are included. The data are expressed as the number of turnovers (Y-axis) per unit time (X-axis). The mutations on KABLE1 are expressed at the subscript. KABLE1.2 is a KABLE1 double mutant carrying the two substitutions, Lys14Pro, and Leu20Trp. KABLE1.3 is a KABLE1 triple mutant carrying the three substitutions, Ile12Phe, Lys14Pro, and Leu20Trp. KABLE1.4 is a quadruple mutant that combines Ile12Phe, Lys14Pro, Leu20Trp, and Leu118Asn. The reaction was measured at pH 8 with a final substrate concentration of 240 μM. **(D)** Global fitting of kinetic parameters for KABLE1.4 in which the initial velocities at a series of substrate concentrations and pHs were used to fit a shared *k*_cat_, *K*_M_ and p*K*_a_. Error bars in D represent standard deviation of three independent measrements.

The ability of ABLE to bind small fragments suggested that its binding site might also accommodate similarly sized substrates for chemical reactions. We therefore explored whether ABLE might be redesigned to catalyze the Kemp elimination, which involves the concerted C–H deprotonation and ring-opening of 5-nitrobenzisoxazole (5NBI) to yield 2-hydroxybenzonitrile (Fig. 4A). This non-natural reaction is mechanistically similar to triosephosphate isomerase and ketosteroid isomerase (38, 39), which use a carboxylate to abstract a proton from a C–H bond of their substrates. Kemp eliminase activity has also been the target of extensive studies in which the active sites of natural enzymes were first re-designed then optimized by directed evolution (29–37, 60). The success rates and catalytic efficiency of such catalysts from the initial round of computational deisgn have been relatively low prior to rational redesign or directed evolution (0 – 200 M^−1^s^−1^) (29, 32, 33, 61, 61–63). Here we ask whether we might have a better success rate starting with a *de novo* scaffold and our knowledge of ABLE’s potential binding interactions.

To design a Kemp eliminase, we targeted a transition state analogue, 6-nitro-benzotriazole (6NBT), in which the C–H group abstracted during catalysis is substituted with a stable N–H group. Similar to previous work (32, 33), we searched for backbone positions of ABLE, in which a Glu or Asp (in a low-energy rotamer) could position 6NBT in a catalytically competent orientation in the pocket delineated in fragment complexes. Leu108Glu was one site chosen because a low-energy rotamer of Glu108 placed the transition state analog inside the aromatic box (Fig. 2C) of the **A** conformer (see section 1.11.1 of SI). We also evaluated His49 as an attractive position for substitution of an Asp residue, because histidine has a similar size, shape and polarity as aspartic acid. Importantly, His49 is located near the bottom of the binding site, where it is a hotspot for interacting with carboxylate-containing fragments (Fig. 4B and fig. S13). As described in section 1.11.2 of SI, Modeling an Asp sidechain at residue 49 reverses the interactions and allows a strong hydrogen bond with 6NBT, using the Asp carboxylate’s more acidic lone pairs of electrons (designated as anti) (65) (Fig. 4A) (see section 1.11.2 of SI). With these restraints in place, we used ligandMPNN and Rosetta FastRelax to optimize the sequence to bind 6NBT while stabilizing the conformation of either Glu108 or Asp49 (47, 54). To increase their basicity, we introduced no more than one H-bond donor to the Asp49 or Glu108 carboxylates, selecting from the three possible sites for H-bonding to the side chain (66). Computed models were filtered based on the RMSD between the design model and that predicted for the unliganded protein computed by RaptorX (67) and ESM2 (49)(to assess preorganization), as well as their pLDDT scores (fig. S14).

We selected five proteins from each strategy for experimental characterization, with pairwise sequence identity ranging from 26% to 71% compared to ABLE (fig. S6). All of the ten proteins expressed well in soluble form (fig. S15) (see section 1.14 of SI). One of the sequences from the first strategy (**A** site) had modest catalytic activity (*k*_cat_/*K*_M_ = 8 M^−1^ s^−1^ at pH 8), which is designated as KABLE0. (fig. S16) (see section 1.15 of SI) A second protein (designated KABLE1) with His49Asp as the base exhibited much greater activity in lysates, and the pH-rate profile of the purified protein had a sigmoidal shape typical of a single-site protonation (fig. S17). Analysis of the pH-rate data showed a maximal *k*_cat_/*K*_M_ of 6600 **±** 2100 M^−1^ s^−1^ and a *p*K_a_ 10.01 **±** 0.10 (*K*_*M*_ = 0.99 **±** 0.29 mM; Table 1, tables S4–5, figs. S17–18) (see section 1.15 of SI). An elevated p*K*_a_ for an Asp residue has previously been seen in a Kemp eliminase based on ketosteroid isomerase (60).

To test that KABLE1 functioned as designed, we first mutated Asp49 to Asn, which eliminated activity (fig. S19). This finding identifies Asp49 as an essential active site residue and also supports its elevated p*K*_a_ (table S4). These findings are consistent with its placement in a hydrophobic environment as designed. Second, we tested whether the hydrogen bonding around Asp49, designed by LigandMPNN, was important. This Asp-triad (comprised of Asp49, Tyr9, and Gln75) stabilizes the carboxylate base while also forming hydrogen bonds with the substrate in the ground state and its leaving group in the transition state (Fig. 4C). Replacement of either Tyr9 and Gln75 also greatly decreased the eliminase activity of KABLE1 (fig. S19). Collectively, these findings strongly support the principles used in the design of KABLE1.

We next used saturation mutagenesis to improve the catalytic efficiency of KABLE1 from a value of *k*_cat_/*K*_M_ from 6,600 M^−1^s^−1^ to 600,000 M^−1^s^−1^ (Table 1, tables S4, figs. S18 and S20). To explore the evolvability of KABLE1, we performed site-saturation mutagenesis of sidechains that line the binding site (within 5.0 Å of the substrate) (see section 1.12–1.14 of SI). In addition, we mutated Lys14, Pro117, and Leu118 because they were proximal to slight kinks in **helices 1** and **4** that might therefore affect the positioning of key groups. Individual substitutions at four positions: Ile12Phe, Lys14Pro, Leu20Trp, and Leu118Asn increased *k*_cat_ by 2- to 5-fold (Fig. 4D, table S4, fig. S18). These substitutions were functionally complementary when combined (Fig. 4D, table S4, fig. S18), increasing the catalytic efficiency by two orders of magnitude in the quadruple mutant, KABLE1.4 (Table 1, Fig 4E, table S4, figs. S18). The improvement in the catalytic efficiency was primarily a result of increase in *k*_cat_, with no significant change in *K*_M_. (table S4, figs. S18).

## Probing the structural and dynamic bases for KABLE’s catalytic performance

**Fig. 5.**
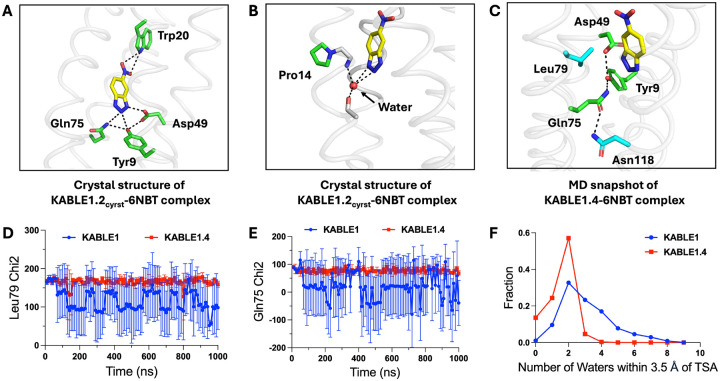
The origin of KABLE’s efficient catalysis probed by MD and X-ray crystallography. (**A**) Structure of the KABLE1.2cryst complex (PDB: 9N0J) showing the Asp-triad engaging the triazole and a Trp indole NH engaging the nitro group at opposite sides of the bound 6NBT. (**B**) Structure of the KABLE1.2cryst complex (PDB: 9N0J) showing Pro14 disrupts the helical geometry, thereby introducing a site to bind a water molecule, which, in turn, forms two additional interactions with the triazole. (**C**) Asp49-Tyr9-Gln75 Triad (green carbons) and key residues, Leu79 and Asn118 (blue carbons) The structure is a representative snapshot from the MD simulation of KABLE1.4 complex with 6NBT (yellow carbons). (**D**) The fluctuation of Gln75’s Chi2 angle versus time during MD simulation. (**E**) The fluctuation of Leu79 Chi2 angle during MD simulation. (**F**) The number of waters within 3.5 Å of OD1 or OD2 of Asp49. Error bars represent standard deviation of three independent measrements.

To determine how these substitutions enhanced catalytic efficiency, we determined two structures by X-ray crystallography (see section 1.16 of SI). Although the high water-solubility of KABLE variants inhibited crystallization, we were able to promote crystallization by introducing substitutions on the surface of the protein. The structures of the variant containing the Lys14Pro and Leu20Trp mutants (designated KABLE1.2_cryst_), which retained 50% of the enzymatic activity of the starting double mutant (fig. S21), in the absence and presence of 6NBT confirm the atomistic details of the designed interactions (< 1.1 Å Cα RMSD relative to the designed KABLE12, table S6). In particular, the hydrogen-bond network within the Asp triad is organized and Asp49 forms a strong (2.5 Å O – N distance) interaction with the transition state analog’s NH group. Gln75 is also in hydrogen-bonded distance to N2 of the triazole (Fig. 5A), and the indole of Leu20Trp formed a weak hydrogen bond (3.3 Å) with the nitro group of 6NBT. Furthermore, Lys14Pro causes a deviation from ideal helix geometry, allowing the mainchain amides to form a hydrogen bond to a water molecule that is well oriented for stabilizing the substrate and transition state complexes (Fig. 5B). Similar catalytically important waters have been observed in other Kemp eliminases (30).

To probe the mechanism for the increase in activity between KABLE1 and KABLE1.4, we performed three independent 1μs molecular dynamics simulations for both proteins in complex with 6NBT. These simulations confirmed that the Asp49 sidechain was well situated to hydrogen bond with the NH group of 6NBT in both complexes. However, three other substrate-contacting sidechains, Gln75, Leu79, and Asn118 had increased rigidity in KABLE1.4 that appeared to quench non-productive dynamics in KABLE1.4.(Figs. 5C–5E, fig. S22). Leu79 helps position the aromatic ring of 6NBT (Fig. 5D), Gln75 is a member of the catalytic triad (Fig. 5E), and the sidechain of the beneficial Leu118Asn mutant formed a hydrogen bond to Gln75. (Figs. 5C and S22). The MD simulations also confirmed the role of two other beneficial mutants seen in the crystal structure of KABLE1.2_cryst_, including the positioning of a water molecule near a helical kink induced by Lys14Pro (fig. S23) and the hydrogen bond involving Leu20Trp (fig. S23). Finally, the mean number of water molecules in contact with 6NBT decreased markedly between KABLE1 to KABLE1.4 (Fig. 5F), consistent with the fact that the rate of the carboxylate-catalyzed Kemp elimination is increased by dehydration of the substrate and the carboxylate (69–71).

## Experimental exploration of the chemical space to probe the specificity of KABLE1.4

**Fig. 6.**
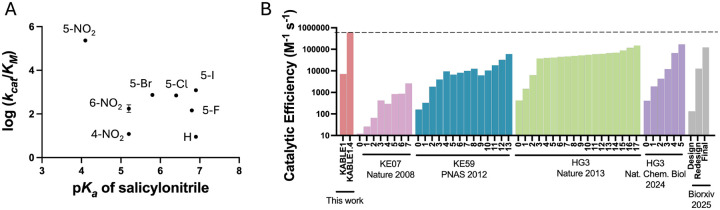
Exploration of sequence and chemical space of *de novo* protein-ligand interactions for designing new functions. (**A**) Bronsted plot for ring-opening of substituted benzisoxazoles points to the unique specialist feature of KABLE1.4. Error bars stand for standard deviations. Error bars represent standard deviation of three independent measrements. (**B**) Catalytic efficiency of designed and evolved Kemp eliminases during experimental optimization. For each protein, the first to the right reflects the activity of the initial designs, which the subsequent bars reflect the activities after: combining mutants from saturation mutagenesis (KABLE); combining favorable mutants in each round of directed evolution (KE07, KE59, HG3), or computational redesign (far right).

Finally, we again returned to chemical space to examine the specificity of KABLE1.4 for analogs of 5NBI. Investigations of previous Kemp eliminases showed that the value of *k*_cat_/*K*_M_ for analogs of 5NBI scaled with the electron-withdrawing character of the leaving group (and hence the overall chemical reactivity of the substrate), rather than the shape and other molecular characteristics of the substrate (60, 68, 72). To determine whether this trend was also applies to KABLE1.4, we evaluated whether the rate of KABLE1.4-catalyzed elimination followed a linear Bronsted relationship over a wide range of electron withdrawing and releasing groups (section 1.17.1–1.17.2 of SI). In contrast to earlier Kemp eliminases, we found no correlation between *k*_cat_/*K*_M_ vs. p*K*_a_ of the corresponding leaving groups for a set of eight substrates (Fig. 6A and figs. S24–26, table S7). This suggests that KABLE1.4 is a specialist because *k*_cat_/*K*_M_ is determined by molecular recognition and not only the intrinsic reactivity of the substrate.

## Discussion:

Inspired by previous evolutionary work (1, 5), we combined chemical screening and *de novo* protein design to introduce new functions into a simple four-helical bundle protein. In Nature, proteins gain new activities by first binding to novel substrates with low affinity, followed by stepwise mutations to increase binding affinity and specificity. The availability of broad, low-level generalist binding activity enables the evolution of multiple specialized activities starting from a single precursor protein. Taking inspiration from this process we used crystallography to characterize ABLE’s ability to weakly bind to a diverse set of fragments, followed by multi-site sequence design to convert ABLE into a specialist binding to turn-on fluorophores. While we used crystallography for screening, a number of other experimental and computational methods for fragment screening that are widely used in small molecule drug discovery (26, 73), could be employed in future applications of our pipeline for protein design.

Using larger moves in sequence space, we also converted ABLE into a Kemp eliminase KABLE1.4, which has 28 % sequence identity to ABLE. The catalytic efficiency of KABLE1.4 (*k*_cat_/*K*_M_ = 600,000 M^−1^ s^−1^, k_cat_ = 800 s^−1^) compares favorably to the median of natural enzymes (*k*_cat_/*K*_M_ = 125,000 M^−1^s^−1^, *k*_cat_ = 13.7 s^−1^) (74) and mechanistically similar enzymes such as triosephosphate isomerase (*k*_cat_/*K*_*M*_ = 440,000 M^−1^s^−1^) (39). It also compares favorably to both rationally designed (30, 31, 36, 60, 68) and computationally designed Kemp eliminases (29, 32, 33, 61–63) in terms of the success rate, the catalytic efficiency of the proteins after direct design and the ease of experimental optimization to high level (Fig. 6B, Table 1, and table S8).

While Kemp eliminases were elaborated from natural protein scaffolds, the combination of a simple *de novo* scaffold and the knowledge of ABLE’s binding proclivities proved to be helpful for design of a Kemp eliminase. In keeping with a first principles approach we considered a number of features hypothesized to be important for catalysis of this reaction (75–77), including the need to match the template to the reaction. The active site carboxylate of the KABLE1 series of proteins is placed within a deep cavity of the protein, which enhances the dehydration and increases the basicity of the carboxylate (69). This placement is made possible by the high stability of ABLE in two ways: First, following the entatic (78) concept of a trade-off between stability and function (79), we used the stability of the fold of KABLE1 to force Asp49 into an apolar environment. The kinetically-derived p*K*_a_ of Asp49 is four units higher than the unperturbed p*K*_a_ of Asp in water, corresponding to a free energy cost of 5.5 kcal/mol. KABLE1’s high thermodynamic stability also allowed the incorporation of four additional substitutions that were functionally beneficial but structurally destabilizing. Lys14Pro introduced a substitution that destabilizes alpha-helices by approximately 3 kcal/mol (80); Leu20Trp introduced a steric clash that required conformational rearrangements; and Leu118Asn replaced a hydrophobic interaction for an H-bonded interaction, which tends to be energetically unfavorable in analogous systems (80, 81). Despite these changes KABLE1.4 remained highly stable (fig. S27).

This work advances at once the *de novo* design of proteins and our understanding of how proteins evolve from generalists to specialists. Here, we used: 1) information from fragment screening; 2) a deep mechanistic understanding of the reaction catalyzed (29, 60, 76, 81), and 3) state-of-the-art computational tools (47, 48, 54) as full partners at each step of our work, from conception to realization of novel function. Thus, blending physical understanding with modern methods for computation design provides a powerful strategy for the design of sensors and catalysts.

## Supplementary Material

Supplement 1

1

## Figures and Tables

**Figure 1:**
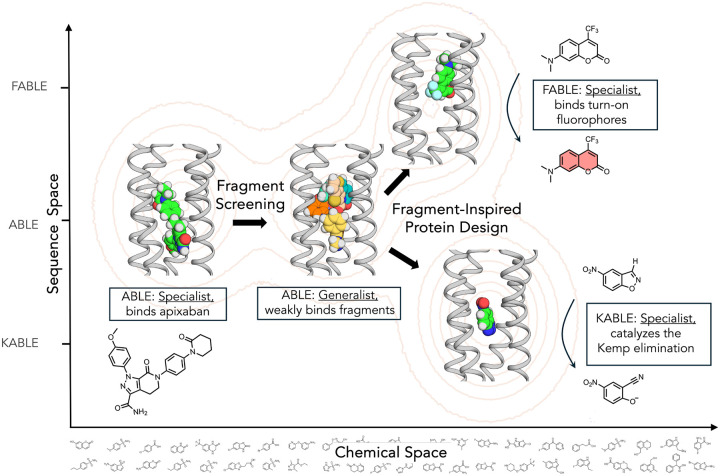
Design of novel functions through exploration of the chemical space and sequence space of protein-ligand interaction via crystallographic fragment screening. Sequence diversity and small molecule chemical diversity are conceptually represented on orthogonal axes, and the contours are meant to denote fitness for binding or catalyzing the Kemp elimination reaction. Starting with ABLE, a specialist for binding apixaban, crystallographic fragment screening revealed ABLE as a generalist for binding 43 chemical fragments. Although the fragments tend to have very low affinity, a set of aromatic fragments informed the design of a fluorescent derivative of ABLE (FABLE), which is a specialist for binding to a turn-on fluorophore. A second set of fragments informed the design of KABLE, an efficient catalyst for the Kemp elimination reaction (*k*_cat_/*K*_M_ = 600,000 M^−1^s^−1^).

**Table 1:** Activities of kemp eliminases for decomposition of 5NBI.

Direct computational design			Experimentally optimized			Reference
Enzyme	*k*_cat_/*K*_M_, M^−1^s^−1^	*k*_cat_, s^−1^	Enzyme	*k*_cat_/*K*_M_, M^−1^s^−1^	*k*_cat_, s^−1^	
KE59	163	0.29	KE59 R13-3/11H	60,400	9.53	(29, 68)
AlleyCat	5.8	ND	AlleyCat10	4,400	21.2	(33, 34)
HG3	425	0.68	HG3.17	150,000	604	(30, 32)
**KABLE1** ^a^	**6,600**	**5.6**	**KABLE1.4** ^a^	**600,000**	**800**	***This work***

a Error bars and methods are at table S4 and section 1.15 of SI. A more extensive table listing additional reported Kemp eliminases is in table S8.

## Data Availability

All data are available in the main text or the supplementary materials.
