# Supplementary material for: Emergence of specific binding and catalysis from a designed generalist binding protein
Source: bioRxiv. 2025 Jul 22:2025.01.30.635804. Originally published 2025 Jan 31. Preprint. [Version 4] doi: 10.1101/2025.01.30.635804 (PMC11838529; doi:10.1101/2025.01.30.635804)
Supplement: Supplement 1 [file media-1.pdf]

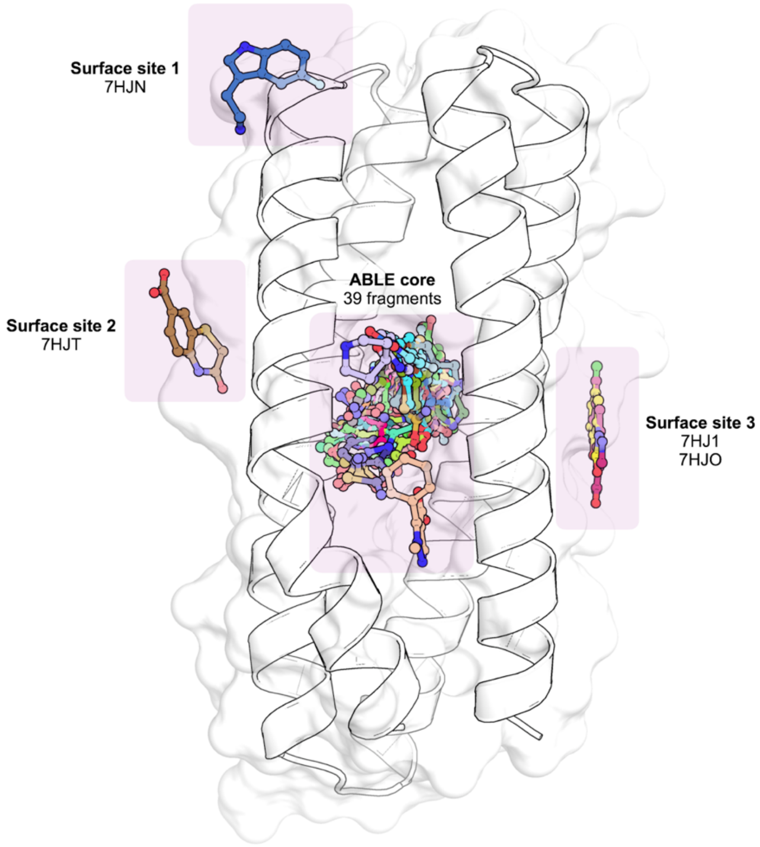

Extended Data Fig. 1 | X-ray crystal structure of ABLE showing binding sites for 43 fragments. The structure of apo ABLE (PDB: 9DW2) with white cartoon and transparent white surface.

ZINC000000156865 | 7HJA
1.59 Å | 0.47 | 94%

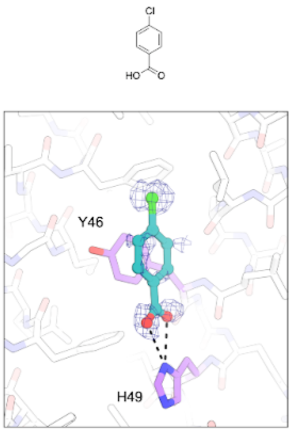

ZINC000000388063 | 7HJ2
1.55 Å | 0.34 | 40%

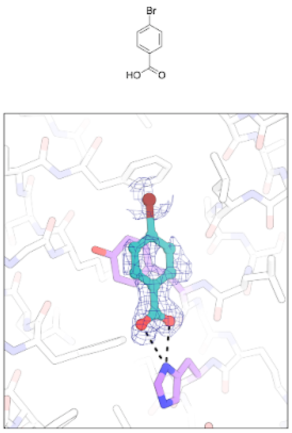

ZINC000000165667 | 7HJL
1.6 Å | 0.24 | 48%

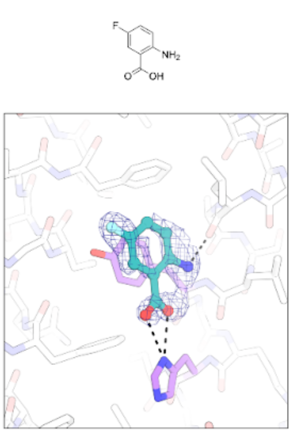

ZINC000000389796 | 7HJY
1.58 Å | 0.45 | 90%

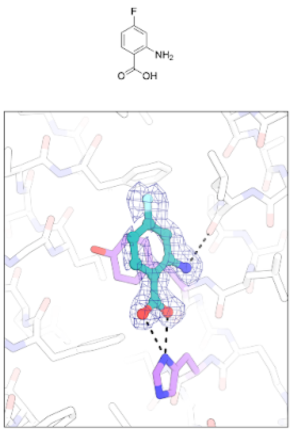

ZINC000000053963 | 7HK3
1.55 Å | 0.11 | 22%

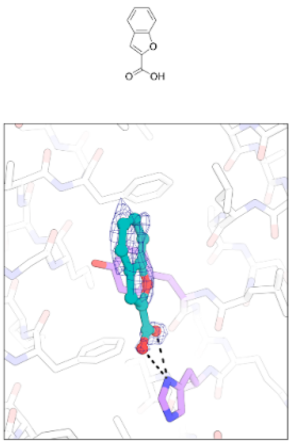

ZINC000000107891 | 7HK1
1.55 Å | 0.17 | 34%

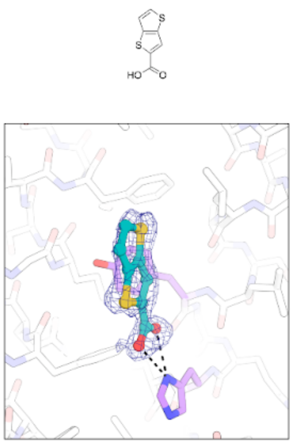

ZINC000000034687 | 7HK2
1.55 Å | 0.41 | 82%

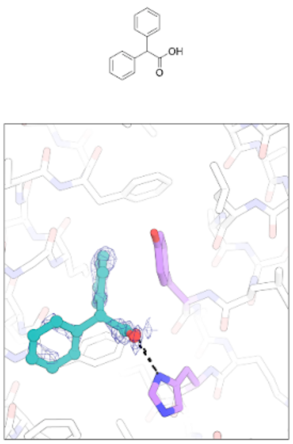

ZINC000000159056 | 7HJH
1.55 Å | 0.18 | 36%

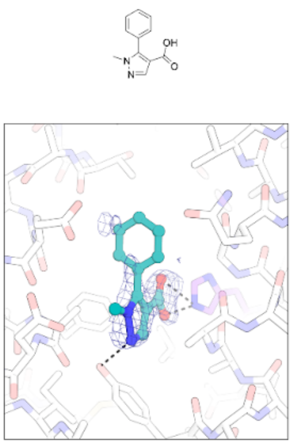

ZINC000000058111 | 7HIY
1.43 Å | 0.26 | 52%

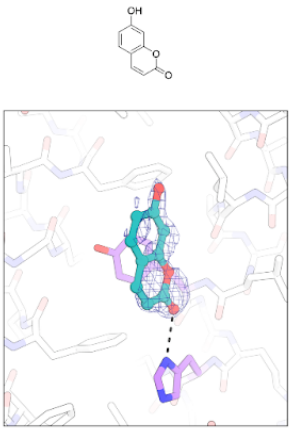

ZINC000008579298 | 7HIZ
1.3 Å | 0.21 | 42%

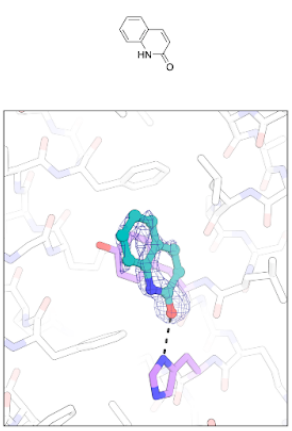

ZINC000000173360 | 7HJV
1.58 Å | 0.08 | 16%

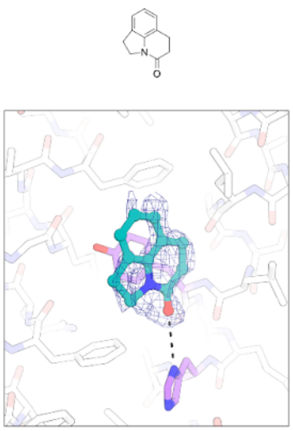

ZINC000004219237 | 7HJC
1.6 Å | 0.2 | 40%

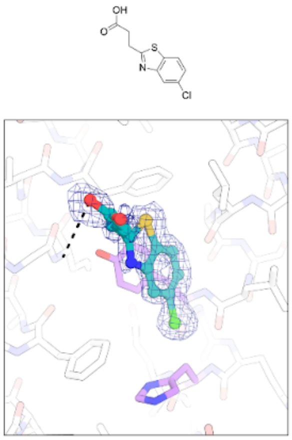

ZINC000000404314 | 7HJI
1.55 Å | 0.24 | 48%

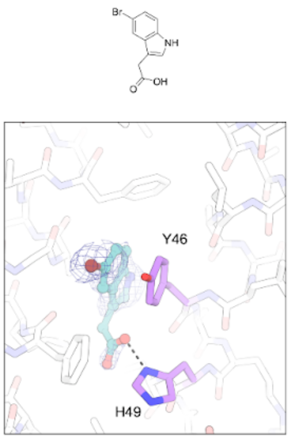

ZINC000006657973 | 7HJ0
1.39 Å | 0.37 | 74%

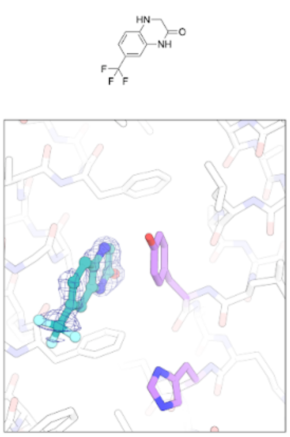

ZINC000017744334 | 7HJQ
1.58 Å | 0.19 | 38%

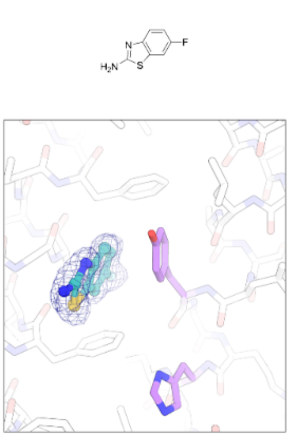

ZINC000000162015 | 7HJ4
1.57 Å | 0.31 | 62%

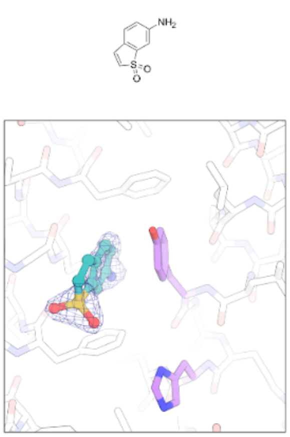

ZINC000001612349 | 7HJ5
1.55 Å | 0.21 | 42%

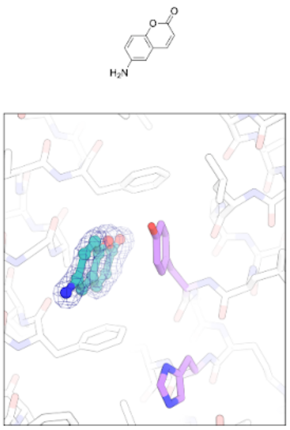

ZINC000016697555 | 7HJZ
1.6 Å | 0.35 | 70%

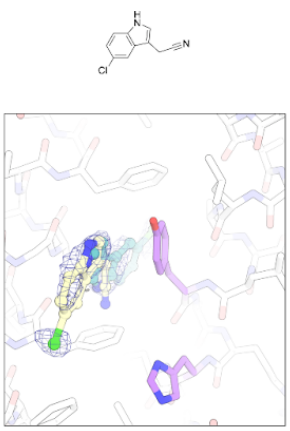

ZINC000004774482 | 7HK0
1.59 Å | 0.21 | 42%

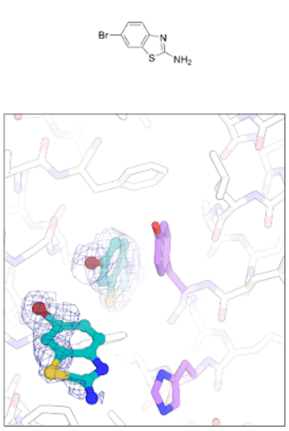

ZINC000038866729 | 7HK4
1.59 Å | 0.14 | 28%

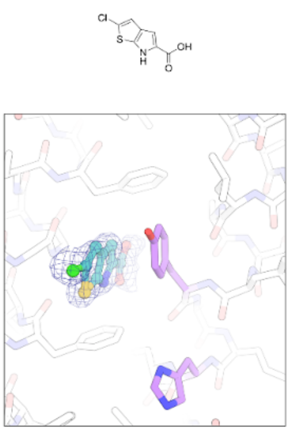

ZINC000001394131 | 7HJG
1.57 Å | 0.26 | 52%

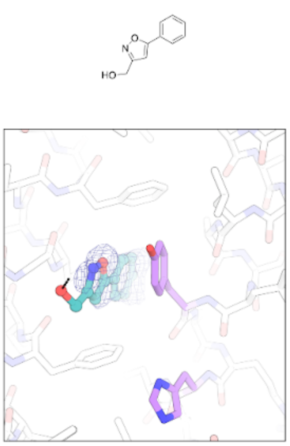

ZINC000000158815 | 7HJJ
1.59 Å | 0.43 | 86%

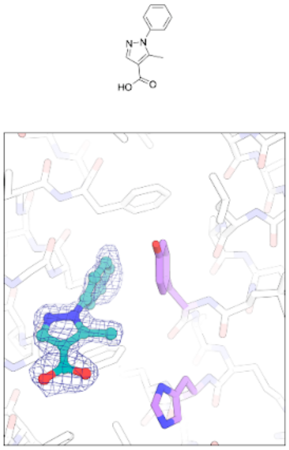

ZINC000000066090 | 7HJX
1.55 Å | 0.19 | 38%

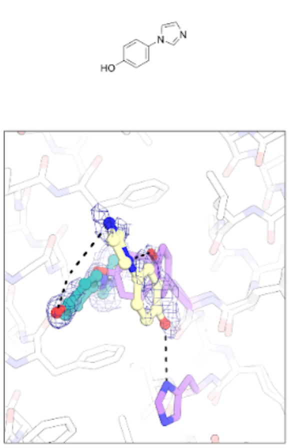

ZINC000012370416 | 7HJF
1.58 Å | 0.23 | 46%

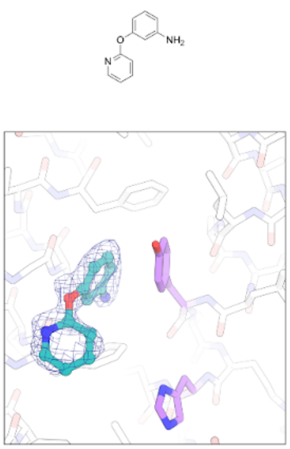

ZINC000002583439 | 7HJK
1.56 Å | 0.12 | 24%

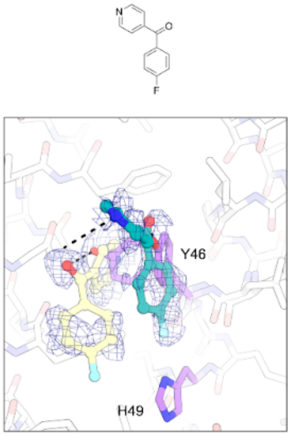

ZINC000002567980 | 7HJE
1.59 Å | 0.13 | 26%

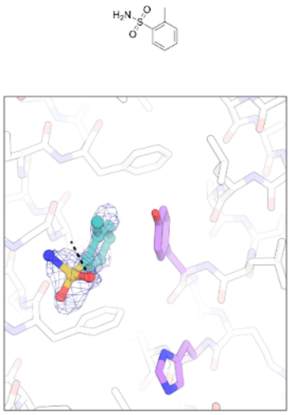

ZINC000000155614 | 7HJ9
1.56 Å | 0.16 | 32%

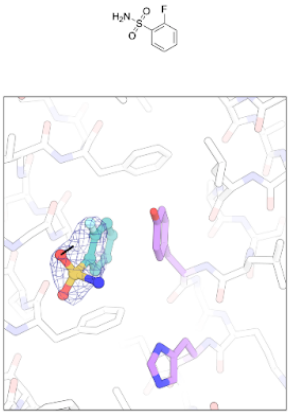

ZINC000002571408 | 7HJW
1.58 Å | 0.22 | 44%

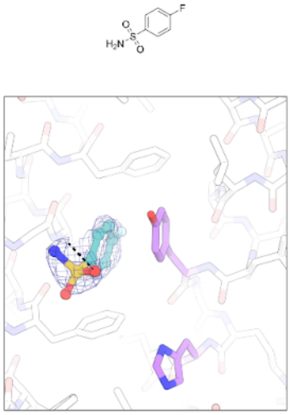

ZINC000000154564 | 7HJB
1.58 Å | 0.11 | 40%

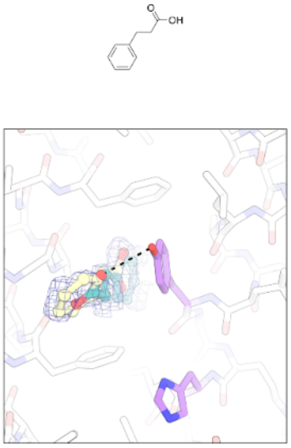

ZINC000000403990 | 7HJ7
1.6 Å | 0.31 | 62%

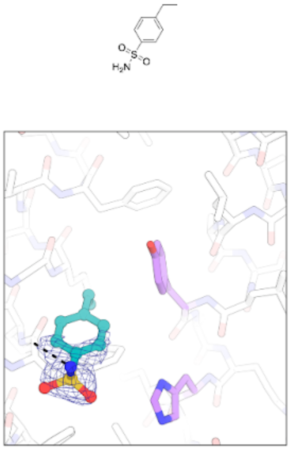

ZINC000000270975 | 7HJ8
1.59 Å | 0.42 | 84%

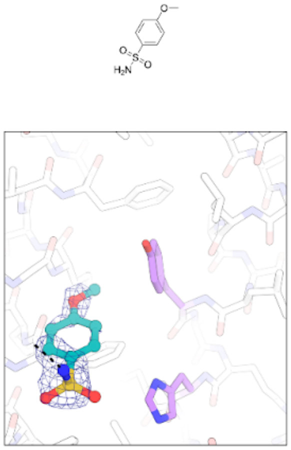

ZINC000002941706 | 7HJ3
1.54 Å | 0.38 | 76%

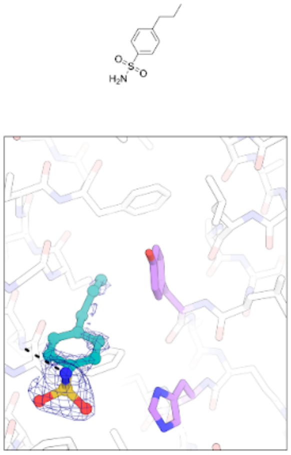

ZINC000004271832 | 7HJ6
1.58 Å | 0.36 | 72%

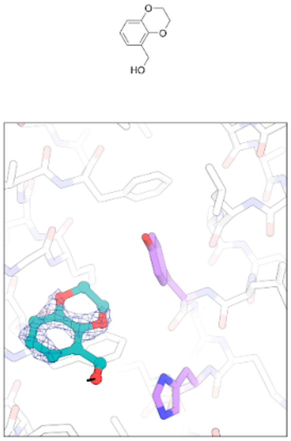

ZINC000000164504 | 7HJD
1.58 Å | 0.38 | 76%

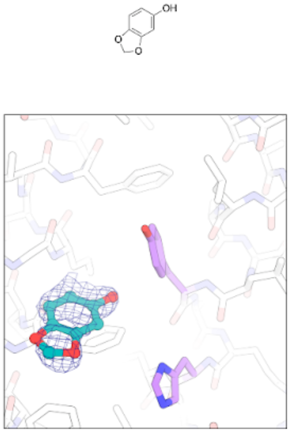

ZINC000000163774 | 7HJM
1.55 Å | 0.11 | 22%

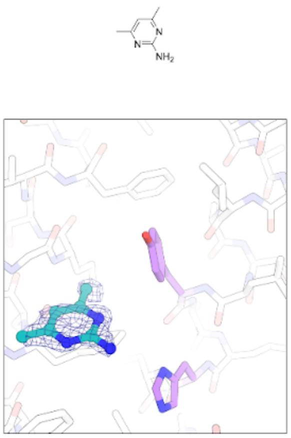

ZINC000000109930 | 7HJS
1.57 Å | 0.41 | 82%

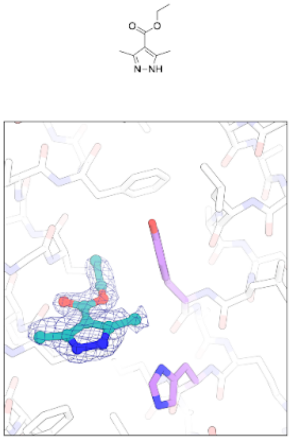

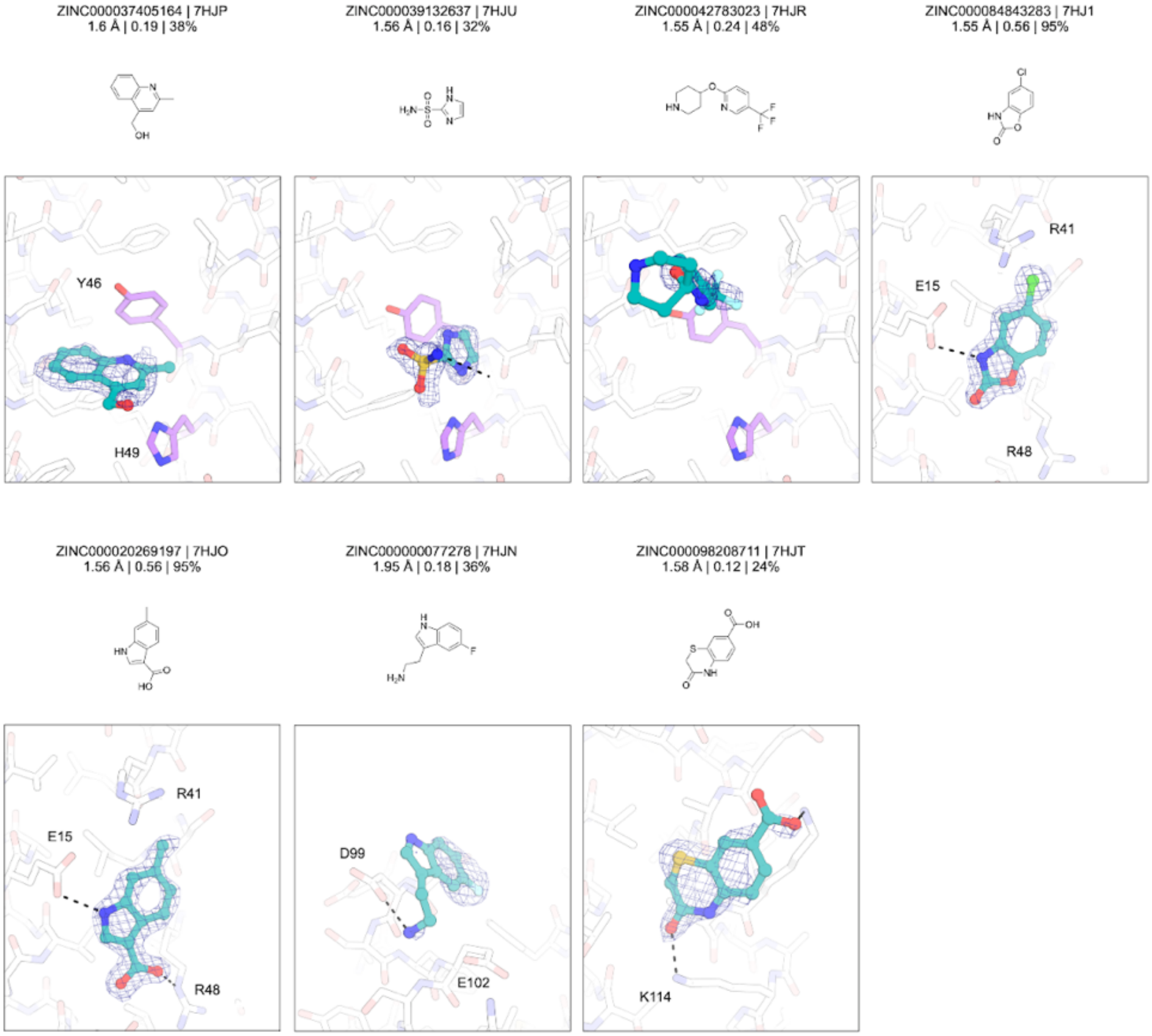

Extended Data Fig. 2 | Chemical structures and electron density maps for the 43 fragment structures reported in this work. PanDDA event maps (blue mesh, 2 σ) are contoured around fragments (teal/yellow sticks). The sidechains of residues Tyr46 and His49 are shown with purple sticks. Fragment names, PDB codes, resolution, 1-BDC value and refined occupancies are indicated. Hydrogen bonds are shown with dashed black lines. For clarity, residues 10-23 and 105-118 are hidden for fragments binding in the ABLE core. His49-mediated polar interaction could be found in the majority of fragments at site B.

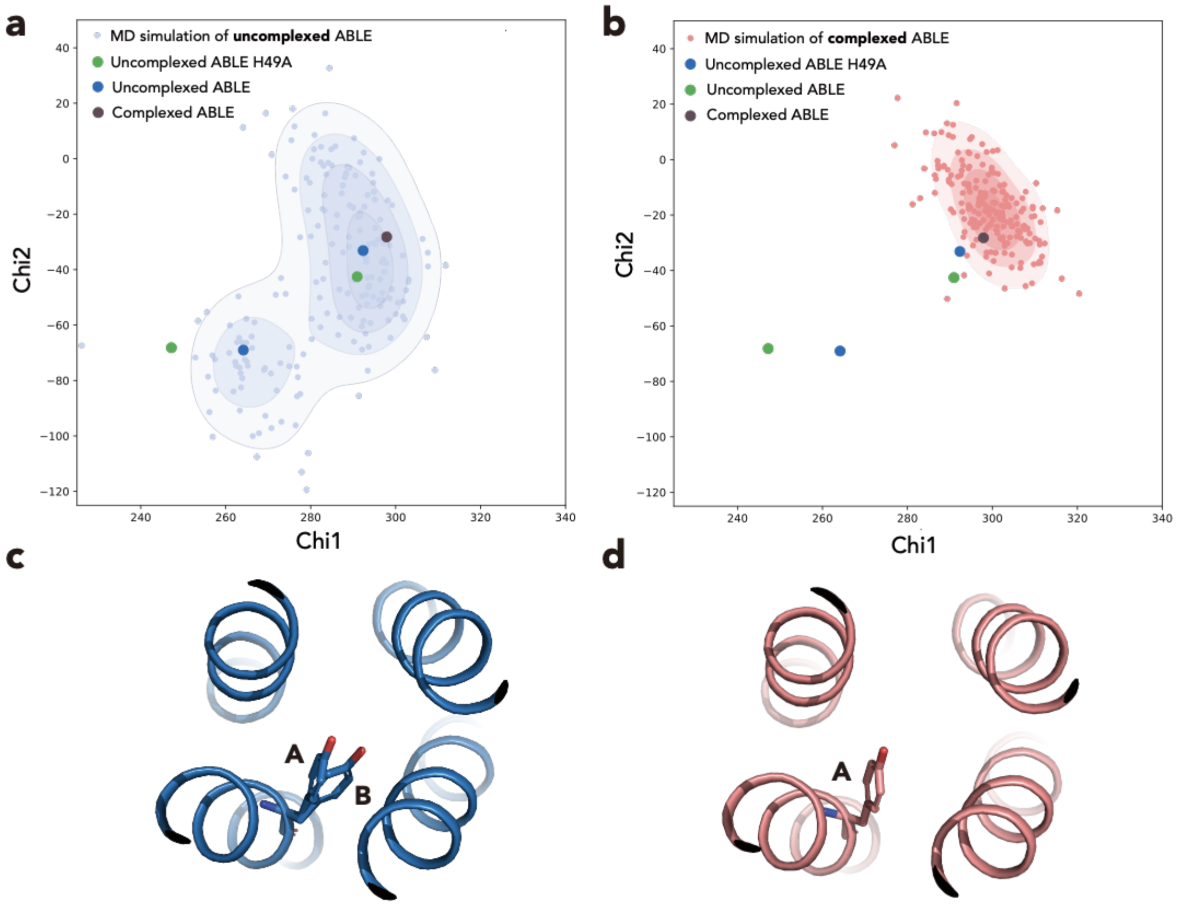

Extended Data Fig. 3 | Contour map of Tyr46 side chain conformation of ABLE from molecular dynamics simulation. (**a&b**) Dynamics of uncomplexed ABLE (PDB: 6W6X) and ABLE-apixaban (PDB: 6W70) were explored by doing molecular simulation at 278 K for 500 ns with Amber. Chi1 and Chi2 from each state of these simulations and reported crystal structures of ABLEs (6W6X: uncompleted ABLE; 6W70: ABLE-Apixaban Complex; 6X8N: uncomplexed ABLE His49Ala) were extracted for plotting. Contours were generated from the points of uncomplexed ABLE (**a**) and ABLE-apixaban complex contours (**b**) were generated using an in-house script that utilizes the Gaussian kernel density estimate methods from scipy.stats module of SciPy Python Packages. (**c&d**) **A** and **B** conformations of Tyr46 sidechain were present in the uncomplexed ABLE crystal structure (**c**, PDB: 6W6X) while only A conformation of Tyr46 sidechain conformations was present at ABLE-apixaban complex (**d**, PDB: 6W70).

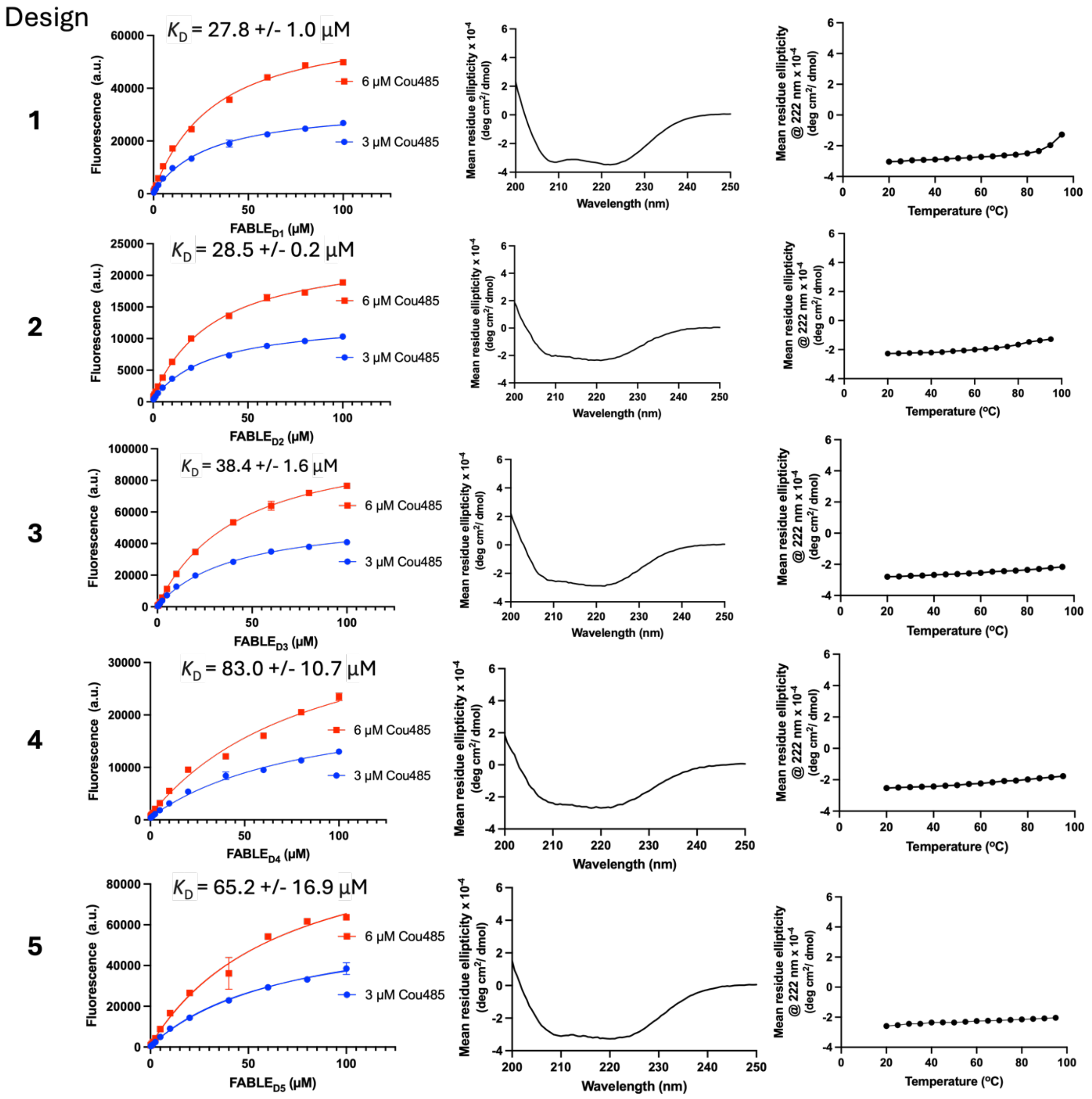

Extended Data Fig. 4 | Characterization of five FABLE Designs. Left column: Fluorescence of Cou485 at two fixed concentrations of 3 μM and 6 μM were measured in the presence of increasing amounts of each protein. The dissociation constant was obtained by globally fitting a single-site binding model to data, as described at Supplementary Methods. The error bars represent standard deviations of three independent measurements. Middle Column: circular dichroism spectra shows that all the designs are helical proteins. Right column: temperature-dependent circular dichroism signals measured at 222 nm show that all designs are thermostable. The design 1 was designated as FABLE.

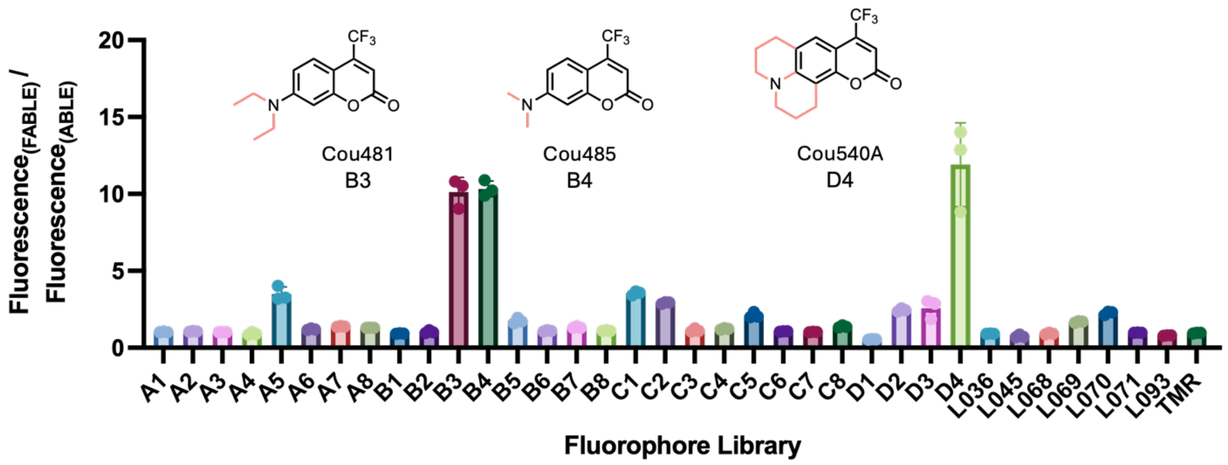

Extended Data Fig. 5 | Exploring the chemical space of FABLE. The excitation and emission of fluorophores are in Supplementary Table 3. The error bars represent standard deviations.

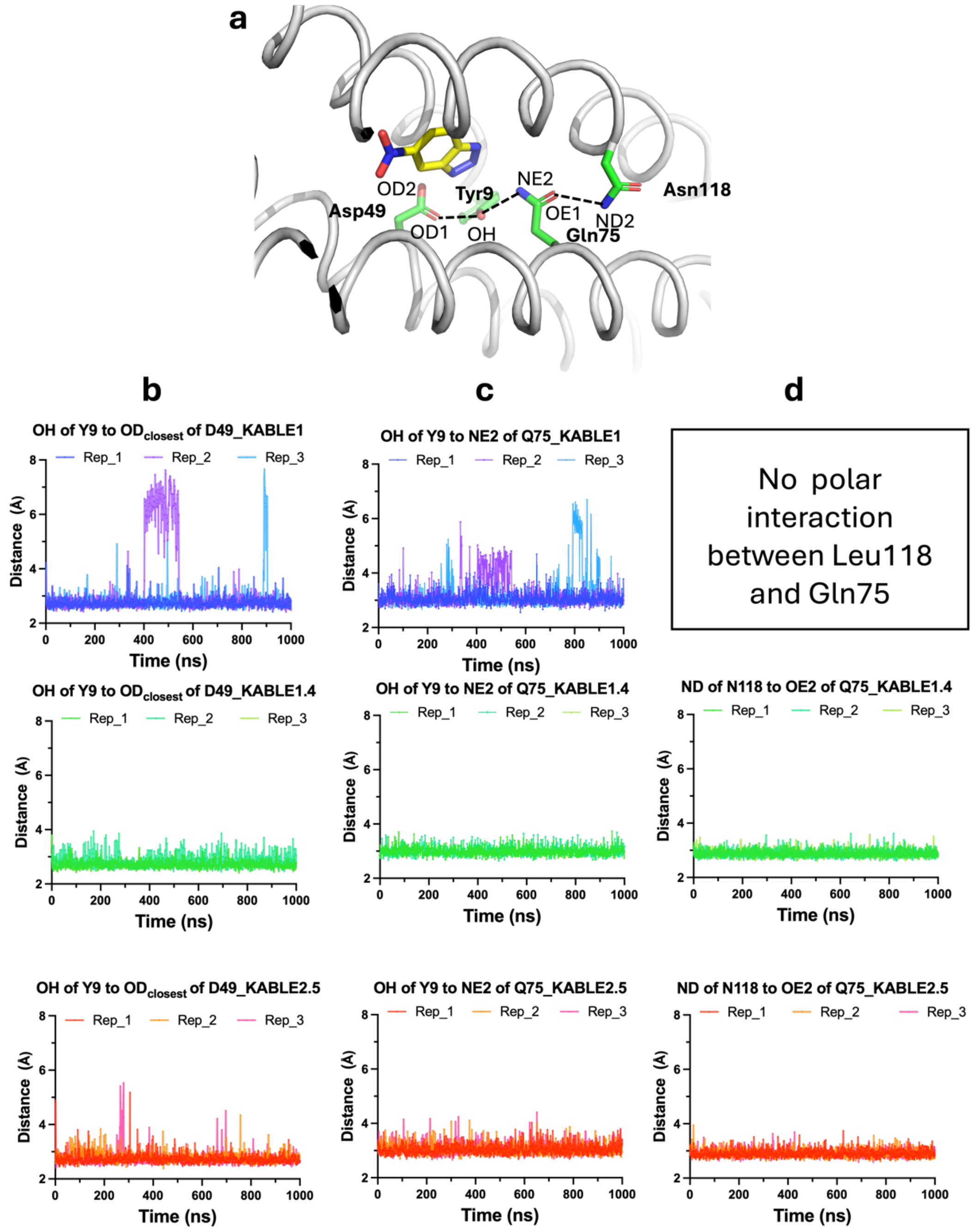

Extended Data Fig. 6 | Stabilization of triad by Leu118Asn.

a, A Representative snapshot of molecular dynamic simulation of KABLE1.4-6NBT complex showing the triad consisting of Asp49, Tyr9, Gln75 and its stabilization residue Asn118, whose sidechain are shown in green sticks. The hydrogen bonding between these 4 residues are annotated with dotted line and their distance are within 2.8-3.2 Å. **b-d,** The fluctuation of indicated distances between indicated atoms at the process of 1000 ns MD simulation of KABLE1.4-6NBT (green) complex, KABLE2.5-6NBT (red) complex, in comparison with KABLE1-6NBT (blue) complex. The distances are extracted using the *label bond* function of VMD. Three independent MD runs are show as three separate curves.

Extended Data Table 1 | pH-dependence of kinetic parameters for KABLEs[a].

Protein	pH	k_{cat}, s^{-1}	K_M, mM	(k_{cat}/K_M), $M^{-1}s^{-1}$
KABLE1	7.0	0.010 ± 0.0006	0.15 ± 0.04,	68 ± 17
	7.5	0.030 ± 0.0007	0.17 ± 0.02,	176 ± 17
	8.0	0.09 ± 0.001,	0.20 ± 0.01	460 ± 20
	8.5	0.11 ± 0.001	0.22 ± 0.01	480 ± 20
	9.0	0.39 ± 0.03	0.12 ± 0.05	3,300 ± 130
	9.5	1.3 ± 0.2	0.50 ± 0.1	2,600 ± 800
	10	1.3 ± 0.2	0.35 ± 0.16	3,700 ± 1,800
KABLE1.4	6.5	10 ± 0.7	0.18 ± 0.05	58,000 ± 16,000
	7.0	30 ± 1.6	0.37 ± 0.05	80,000 ± 12,000
	7.5	65 ± 5	0.38 ± 0.03	170,000 ± 20,000
	8.0	99 ± 5	0.43 ± 0.05	230,000 ± 32,000
	8.5	180 ± 6	0.49 ± 0.03	360,000 ± 28,000
	9.0	210 ± 4	0.40 ± 0.02	530,000 ± 26,000
	9.5	270 ± 3	0.44 ± 0.01	610,000 ± 16,000
	10	290 ± 5	0.49 ± 0.02	610,000 ± 28,000
KABLE2.5	6.5	36 ± 3	0.11 ± 0.04	330,000 ± 130,000
	7.0	140 ± 5	0.25 ± 0.03	510,000 ± 67,000
	7.5	210 ± 6	0.30 ± 0.03	640,000 ± 58,000
	8.0	230 ± 5	0.20 ± 0.02	1,100,000 ± 92,000
	8.5	340 ± 10	0.27 ± 0.03	1,300,000 ± 140,000
	9.0	470 ± 7	0.24 ± 0.01	2,000,000 ± 93,000
	9.5	544 ± 10	0.25 ± 0.01	2,200,000 ± 140,000
	10	570 ± 20	0.27 ± 0.02	2,100,000 ± 180,000

[a]Error bars represent the standard errors of the mean from three independent measurements.

Extended Data Table 2 | Analysis of pH-dependence of kinetic parameters for KABLEs[a]

**Protein**	pH range	k_{cat}, s^{-1}	(k_{cat}/K_M), $M^{-1}s^{-1}$	pK_a [b]
KABLE1	7.0-10	1.7 ± 0.2	3,800 ± 1,400	9.3 ± 0.2
KABLE1.4	6.5-10	290 ± 70	630,000 ± 19,000	8.4 ± 0.1
KABLE2.5	6.5-10	570 ± 20	2,200,000 ± 100,000	8.3 ± 0.1

[a]Error bars represent the standard errors of the mean from three independent measurements. Maximum values from fitting the pH-dependence of kinetic parameters were used.
[b]Values were from fitting pH-dependence of k_{cat}.

Extended Data Table 3 | Kinetic parameters[a] of KABLE2.5 and HG3.17.

**Protein**	k_{cat}, s^{-1}	K_M, mM	(k_{cat}/K_M), $M^{-1}s^{-1}$
HG3.17[b]	106 ± 17	0.51 ± 0.25	121,700 ± 40,500
KABLE2.5[b]	508 ± 23	0.51 ± 0.06	832,700 ± 86,300
HG3.17[c]	56.3 ± 9.0	0.91 ± 0.25	62,100 ± 19,700
KABLE2.5[c]	274 ± 14	0.37 ± 0.06	651,000 ± 105,600
HG3.17[d]	30.4 ± 2.7	0.42 ± 0.09	72,300 ± 15,500
KABLE2.5[d]	291 ± 13	0.38 ± 0.04	768,500 ± 95,700

[a]Error bars represent the standard errors of the mean from three independent measurements. Assay conditions are specified by the superscript.
[b-d]Assay conditions reported for measuring the activity of HG3.17[61].
[b]Assay conditions: 50 mM sodium phosphate, pH 7.0; 100 mM NaCl; 10% MeOH, 27 ºC.
[c]Assay conditions: 50 mM sodium phosphate, pH 7.5; 100 mM NaCl; 10% MeOH, 27 ºC.
[d]Assay conditions: 50 mM Bis-tris propane, pH 8.0; 100 mM NaCl; 10% MeOH, 27 ºC.

Extended Data Table 4 | Kinetic parameters of reported proteins catalyzing the Kemp elimination of 5-nitrobenzisoxazole with base-mediated mechanism.

Protein	Description	k_{cat}/K_M, $M^{-1}s^{-1}$	k_{cat}, s^{-1}	K_M, mM	Amino acid efficiency[a]	Ref
34E4	Catalytic antibody	5500	0.66	1.2	26	135
BSA	Natural lipid carrier	6500	6.02	ND	11	136
KE07	Computational redesign	12.2	0.018	1.4	0.05	60
KE59	Computational redesign	163	0.29	1.8	0.66	60
KE70	Computational redesign	78	0.16	2.1	0.31	60
KE07 R7 10/11G	7 round DE of KE07	2590	1.37	0.54	10	77
KE59 R13_3/11H	13 round DE of KE59	60,430	9.53	0.16	240	108
KE70 R8 15/11E	8 round DE of KE70	34,900	5.3	0.15	140	137
HG3	Computational redesign and rational engineering	425	0.68	1.6	1.4	63
HG3.17	17 round DE of HG3	150,000	604	3.5	500	61,62
HG3.R5	5 round DE of HG3	170,000	702	4.8	560	62
HG4	Ensemble-based redesign of HG3	103,000	ND	ND	340	66
HG649	Ensemble-based redesign of HG3	32,000	ND	ND	110	123
AlleyCat	Single mutation of Calmodulin	5.8	ND	ND	0.08	64
AlleyCat10	NMR-guided DE of AlleyCat7	4378	21.2	4.8	59	65
Lysozyme L99A/M102H	Double mutation of Lysozyme	1.8	ND	ND	0.01	138
GNCA4 W229D F290W	Double mutation of GNCA4	5497	10	ND	21	67
V4	DE of GNCA4 W229D F290W	200,000	635	3.14	710	139
KE15	Computational redesign	35	0.022	0.63	0.14	60
KE15 Tyr167Lys+ R4	Electrical field-guided design of KE15	403	0.31	0.77	1.6	73
tKSI	Natural enzyme	2.5	ND	ND	0.02	68

D38N tKSI	Single mutation of tKSI	17,000	ND	ND	140	[68]
Des27	Computational design	130	0.07	0.5	0.52	[71]
Des27.7	Computational redesign	12,700	2.85	0.21	51	[71]
F113L Des27.7	Single mutation on Des27.7	123,000	30	ND	490	[71]
**KABLE1**	De Novo Design	**3,800**	**1.7**	**0.45**	**33**	This work
**KABLE1.4**	Quadruple mutant of KABLE1	**630,000**	**290**	**0.46**	**5,400**	This work
**KABLE2.5**	NMR-guided DE of KABLE1.4	**2,200,000**	**570**	**0.26**	**19,000**	This work

[a]Amino acid catalytic efficiency, which is the quotient of catalytic efficiency (k_{cat}/K_M, $M^{-1}s^{-1}$), by the total number of amino acid for each protein.

Reference for the entire manuscript:

1. Eck, R. V. & Dayhoff, M. O. Evolution of the structure of ferredoxin based on living relics of primitive amino Acid sequences. *Science* **152**, 363–366 (1966).
2. Alva, V., Söding, J. & Lupas, A. N. A vocabulary of ancient peptides at the origin of folded proteins. *eLife* **4**, e09410 (2015).
3. Romero Romero, M. L., Rabin, A. & Tawfik, D. S. Functional Proteins from Short Peptides: Dayhoff's Hypothesis Turns 50. *Angew Chem Int Ed Engl* **55**, 15966–15971 (2016).
4. Söding, J. & Lupas, A. N. More than the sum of their parts: on the evolution of proteins from peptides. *Bioessays* **25**, 837–846 (2003).
5. Ohno, S. Ancient Linkage Groups and Frozen Accidents. *Nature* **244**, 259–262 (1973).
6. Tokuriki, N. & Tawfik, D. S. Protein dynamism and evolvability. *Science* **324**, 203–207 (2009).
7. Aharoni, A. *et al.* The 'evolvability' of promiscuous protein functions. *Nat Genet* **37**, 73–76 (2005).
8. Soskine, M. & Tawfik, D. S. Mutational effects and the evolution of new protein functions. *Nat Rev Genet* **11**, 572–582 (2010).
9. Eisenberg, D. *et al.* The design, synthesis, and crystallization of an alpha-helical peptide. *Proteins* **1**, 16–22 (1986).
10. Ho, S. P. & DeGrado, W. F. Design of a 4-helix bundle protein: synthesis of peptides which self-associate into a helical protein. *J. Am. Chem. Soc.* **109**, 6751–6758 (1987).
11. Regan, L. & DeGrado, W. F. Characterization of a Helical Protein Designed from First Principles. *Science* **241**, 976–978 (1988).
12. Kaplan, J. & DeGrado, W. F. De novo design of catalytic proteins. *Proceedings of the National Academy of Sciences* **101**, 11566–11570 (2004).
13. Faiella, M. *et al.* An artificial di-iron oxo-protein with phenol oxidase activity. *Nat Chem Biol* **5**, 882–884 (2009).
14. Farinas, E. & Regan, L. The de novo design of a rubredoxin-like fe site. *Protein Science* **7**, 1939–1946 (1998).
15. Nanda, V. *et al.* De Novo Design of a Redox-Active Minimal Rubredoxin Mimic. *J. Am. Chem. Soc.* **127**, 5804–5805 (2005).
16. Lombardi, A. *et al.* Retrostructural analysis of metalloproteins: Application to the design of a minimal model for diiron proteins. *Proceedings of the National Academy of Sciences* **97**, 6298–6305 (2000).
17. Nanda, V. & Koder, R. L. Designing artificial enzymes by intuition and computation. *Nature Chem* **2**, 15–24 (2010).
18. Huang, P.-S., Boyken, S. E. & Baker, D. The coming of age of de novo protein design. *Nature* **537**, 320–327 (2016).
19. Korendovych, I. V. & DeGrado, W. F. De novo protein design, a retrospective. *Quarterly Reviews of Biophysics* **53**, e3 (2020).
20. Kortemme, T. De novo protein design—From new structures to programmable functions. *Cell* **187**, 526–544 (2024).
21. Albanese, K. I., Barbe, S., Tagami, S., Woolfson, D. N. & Schiex, T. Computational protein design. *Nat Rev Methods Primers* **5**, 1–28 (2025).
22. Qing, R. *et al.* Protein Design: From the Aspect of Water Solubility and Stability. *Chem. Rev.* **122**, 14085–14179 (2022).
23. Joh, N. H. *et al.* De novo design of a transmembrane Zn2+-transporting four-helix bundle. *Science* **346**, 1520–1524 (2014).
24. Yeh, A. H.-W. *et al.* De novo design of luciferases using deep learning. *Nature* **614**, 774–780 (2023).
25. Lu, L. *et al.* De novo design of drug-binding proteins with predictable binding energy and specificity. *Science* **384**, 106–112 (2024).
26. Mravic, M. *et al.* De novo-designed transmembrane proteins bind and regulate a cytokine receptor. *Nat Chem Biol* **20**, 751–760 (2024).
27. Du, H. *et al.* Targeting peptide antigens using a multiallelic MHC I-binding system. *Nat Biotechnol* 1–11 (2024) doi:10.1038/s41587-024-02505-8.

28. Der, B. S., Edwards, D. R. & Kuhlman, B. Catalysis by a de novo zinc-mediated protein interface: implications for natural enzyme evolution and rational enzyme engineering. *Biochemistry* **51**, 3933–3940 (2012).
29. Studer, S. *et al.* Evolution of a highly active and enantiospecific metalloenzyme from short peptides. *Science* **362**, 1285–1288 (2018).
30. Basler, S. *et al.* Efficient Lewis acid catalysis of an abiological reaction in a de novo protein scaffold. *Nat. Chem.* **13**, 231–235 (2021).
31. Stenner, R., Steventon, J. W., Seddon, A. & Anderson, J. L. R. A de novo peroxidase is also a promiscuous yet stereoselective carbene transferase. *Proceedings of the National Academy of Sciences* **117**, 1419–1428 (2020).
32. Schnettler, J. D. *et al.* Selection of a promiscuous minimalist cAMP phosphodiesterase from a library of de novo designed proteins. *Nat. Chem.* **16**, 1200–1208 (2024).
33. Collins, P. M. *et al.* Achieving a Good Crystal System for Crystallographic X-Ray Fragment Screening. *Methods Enzymol* **610**, 251–264 (2018).
34. Schuller, M. *et al.* Fragment binding to the Nsp3 macrodomain of SARS-CoV-2 identified through crystallographic screening and computational docking. *Sci Adv* **7**, eabf8711 (2021).
35. Pearce, N. M. *et al.* A multi-crystal method for extracting obscured crystallographic states from conventionally uninterpretable electron density. *Nat Commun* **8**, 15123 (2017).
36. Polizzi, N. F. & DeGrado, W. F. A defined structural unit enables de novo design of small-molecule–binding proteins. *Science* **369**, 1227–1233 (2020).
37. Bar-Even, A. *et al.* The moderately efficient enzyme: evolutionary and physicochemical trends shaping enzyme parameters. *Biochemistry* **50**, 4402–4410 (2011).
38. Putman, S. J., Coulson, A. F., Farley, I. R., Riddleston, B. & Knowles, J. R. Specificity and kinetics of triose phosphate isomerase from chicken muscle. *Biochem J* **129**, 301–310 (1972).
39. Yin, H. *et al.* Computational Design of Peptides That Target Transmembrane Helices. *Science* **315**, 1817–1822 (2007).
40. Reig, A. J. *et al.* Alteration of the oxygen-dependent reactivity of de novo Due Ferri proteins. *Nature Chem* **4**, 900–906 (2012).
41. Rufo, C. M. *et al.* Short peptides self-assemble to produce catalytic amyloids. *Nature Chem* **6**, 303–309 (2014).
42. Mann, S. I., Nayak, A., Gassner, G. T., Therien, M. J. & DeGrado, W. F. De Novo Design, Solution Characterization, and Crystallographic Structure of an Abiological Mn-Porphyrin-Binding Protein Capable of Stabilizing a Mn(V) Species. *J Am Chem Soc* **143**, 252–259 (2021).
43. Rosenbaum, D. M., Rasmussen, S. G. F. & Kobilka, B. K. The structure and function of G-protein-coupled receptors. *Nature* **459**, 356–363 (2009).
44. Pillai, A. S. *et al.* Origin of complexity in haemoglobin evolution. *Nature* **581**, 480–485 (2020).
45. Huang, J., Pan, X. & Yan, N. Structural biology and molecular pharmacology of voltage-gated ion channels. *Nat Rev Mol Cell Biol* **25**, 904–925 (2024).
46. Nishi, T. & Forgac, M. The vacuolar (H+)-ATPases — nature's most versatile proton pumps. *Nat Rev Mol Cell Biol* **3**, 94–103 (2002).
47. Letts, J. A., Fiedorczuk, K. & Sazanov, L. A. The architecture of respiratory supercomplexes. *Nature* **537**, 644–648 (2016).
48. Nozawa, K. *et al.* Pyrrolysyl-tRNA synthetase–tRNAPyl structure reveals the molecular basis of orthogonality. *Nature* **457**, 1163–1167 (2009).
49. Williams, P. A. *et al.* Crystal structure of human cytochrome P450 2C9 with bound warfarin. *Nature* **424**, 464–468 (2003).
50. Gahbauer, S. *et al.* Iterative computational design and crystallographic screening identifies potent inhibitors targeting the Nsp3 macrodomain of SARS-CoV-2. *Proc Natl Acad Sci U S A* **120**, e2212931120 (2023).
51. Aschenbrenner, J. C. *et al.* Identifying novel chemical matter against the Chikungunya virus nsP3 macrodomain through crystallographic fragment screening. *bioRxiv* 2024.08.23.609196 (2024) doi:10.1101/2024.08.23.609196.
52. Khersonsky, O. & Tawfik, D. S. Enzyme promiscuity: a mechanistic and evolutionary perspective. *Annu Rev Biochem* **79**, 471–505 (2010).
53. Nad, S. & Pal, H. Unusual Photophysical Properties of Coumarin-151. *J. Phys. Chem. A* **105**, 1097–1106 (2001).
54. Polizzi, N. F. *et al.* De novo design of a hyperstable non-natural protein–ligand complex with sub-Å accuracy. *Nature Chem* **9**, 1157–1164 (2017).
55. Das, R. & Baker, D. Macromolecular Modeling with Rosetta. *Annual Review of Biochemistry* **77**, 363–382 (2008).

56. Wu, R. *et al.* High-resolution de novo structure prediction from primary sequence. 2022.07.21.500999 Preprint at https://doi.org/10.1101/2022.07.21.500999 (2022).
57. Solvent and Environmental Effects. in *Principles of Fluorescence Spectroscopy* (ed. Lakowicz, J. R.) 205–235 (Springer US, Boston, MA, 2006). doi:10.1007/978-0-387-46312-4_6.
58. Kuntz, I. D., Chen, K., Sharp, K. A. & Kollman, P. A. The maximal affinity of ligands. *Proceedings of the National Academy of Sciences* **96**, 9997–10002 (1999).
59. Hopkins, A. L., Keserü, G. M., Leeson, P. D., Rees, D. C. & Reynolds, C. H. The role of ligand efficiency metrics in drug discovery. *Nat Rev Drug Discov* **13**, 105–121 (2014).
60. Röthlisberger, D. *et al.* Kemp elimination catalysts by computational enzyme design. *Nature* **453**, 190–195 (2008).
61. Blomberg, R. *et al.* Precision is essential for efficient catalysis in an evolved Kemp eliminase. *Nature* **503**, 418–421 (2013).
62. Patsch, D. *et al.* Enriching productive mutational paths accelerates enzyme evolution. *Nat Chem Biol* **20**, 1662–1669 (2024).
63. Privett, H. K. *et al.* Iterative approach to computational enzyme design. *Proceedings of the National Academy of Sciences* **109**, 3790–3795 (2012).
64. Korendovych, I. V. *et al.* Design of a switchable eliminase. *Proceedings of the National Academy of Sciences* **108**, 6823–6827 (2011).
65. Bhattacharya, S. *et al.* NMR-guided directed evolution. *Nature* **610**, 389–393 (2022).
66. Broom, A. *et al.* Ensemble-based enzyme design can recapitulate the effects of laboratory directed evolution in silico. *Nat Commun* **11**, 4808 (2020).
67. Risso, V. A. *et al.* De novo active sites for resurrected Precambrian enzymes. *Nat Commun* **8**, 16113 (2017).
68. Lamba, V. *et al.* Kemp Eliminase Activity of Ketosteroid Isomerase. *Biochemistry* **56**, 582–591 (2017).
69. Bunzel, H. A. *et al.* Evolution of dynamical networks enhances catalysis in a designer enzyme. *Nat. Chem.* **13**, 1017–1022 (2021).
70. Schwans, J. P., Kraut, D. A. & Herschlag, D. Determining the catalytic role of remote substrate binding interactions in ketosteroid isomerase. *Proc Natl Acad Sci U S A* **106**, 14271–14275 (2009).
71. Listov, D. *et al.* Complete computational design of high-efficiency Kemp elimination enzymes. *Nature* 1–7 (2025)
72. Bhowmick, A., Sharma, S. C. & Head-Gordon, T. The Importance of the Scaffold for de Novo Enzymes: A Case Study with Kemp Eliminase. *J. Am. Chem. Soc.* **139**, 5793–5800 (2017).
73. Vaissier, V., Sharma, S. C., Schaettle, K., Zhang, T. & Head-Gordon, T. Computational Optimization of Electric Fields for Improving Catalysis of a Designed Kemp Eliminase. *ACS Catal.* **8**, 219–227 (2018).
74. Donald, J. E., Kulp, D. W. & DeGrado, W. F. Salt bridges: geometrically specific, designable interactions. *Proteins* **79**, 898–915 (2011).
75. Robinson, A. C., Castañeda, C. A., Schlessman, J. L. & García-Moreno, E. B. Structural and thermodynamic consequences of burial of an artificial ion pair in the hydrophobic interior of a protein. *Proc Natl Acad Sci U S A* **111**, 11685–11690 (2014).
76. Dauparas, J. *et al.* Atomic context-conditioned protein sequence design using LigandMPNN. *Nat Methods* **22**, 717–723 (2025).
77. Khersonsky, O. *et al.* Evolutionary optimization of computationally designed enzymes: Kemp eliminases of the KE07 series. *J Mol Biol* **396**, 1025–1042 (2010).
78. Jing, X., Wu, F., Luo, X. & Xu, J. RaptorX-Single: single-sequence protein structure prediction by integrating protein language models. 2023.04.24.538081 Preprint at https://doi.org/10.1101/2023.04.24.538081 (2023).
79. Lin, Z. *et al.* Evolutionary-scale prediction of atomic-level protein structure with a language model. *Science* **379**, 1123–1130 (2023).
80. Merlicek, L. P. *et al.* AI.zymes: A Modular Platform for Evolutionary Enzyme Design. *Angewandte Chemie International Edition* **n/a**, e202507031.
81. Gutierrez-Rus, L. I. *et al.* Enzyme Enhancement Through Computational Stability Design Targeting NMR-Determined Catalytic Hotspots. *J. Am. Chem. Soc.* **147**, 14978–14996 (2025).
82. Casey, M. L., Kemp, D. S., Paul, K. G. & Cox, D. D. Physical organic chemistry of benzisoxazoles. I. Mechanism of the base-catalyzed decomposition of benzisoxazoles. *J. Org. Chem.* **38**, 2294–2301 (1973).
83. Frushicheva, M. P., Cao, J., Chu, Z. T. & Warshel, A. Exploring challenges in rational enzyme design by simulating the catalysis in artificial kemp eliminase. *Proceedings of the National Academy of Sciences* **107**, 16869–16874 (2010).

84. Wang, P., Zhang, J., Zhang, S., Lu, D. & Zhu, Y. Using High-Throughput Molecular Dynamics Simulation to Enhance the Computational Design of Kemp Elimination Enzymes. *J. Chem. Inf. Model.* **63**, 1323–1337 (2023).
85. Romero, P. A. & Arnold, F. H. Exploring protein fitness landscapes by directed evolution. *Nat Rev Mol Cell Biol* **10**, 866–876 (2009).
86. Packer, M. S. & Liu, D. R. Methods for the directed evolution of proteins. *Nat Rev Genet* **16**, 379–394 (2015).
87. Erlanson, D. A., Fesik, S. W., Hubbard, R. E., Jahnke, W. & Jhoti, H. Twenty years on: the impact of fragments on drug discovery. *Nat Rev Drug Discov* **15**, 605–619 (2016).
88. Chen, Y. & Shoichet, B. K. Molecular docking and ligand specificity in fragment-based inhibitor discovery. *Nat Chem Biol* **5**, 358–364 (2009).
89. Bedard, P. L., Hyman, D. M., Davids, M. S. & Siu, L. L. Small molecules, big impact: 20 years of targeted therapy in oncology. *The Lancet* **395**, 1078–1088 (2020).
90. An, L. *et al.* Binding and sensing diverse small molecules using shape-complementary pseudocycles. *Science* **385**, 276–282 (2024).
91. Yang, W. & Lai, L. Computational design of ligand-binding proteins. *Current Opinion in Structural Biology* **45**, 67–73 (2017).
92. Tinberg, C. E. *et al.* Computational design of ligand-binding proteins with high affinity and selectivity. *Nature* **501**, 212–216 (2013).
93. Dou, J. *et al.* De novo design of a fluorescence-activating β-barrel. *Nature* **561**, 485–491 (2018).
94. Tantillo, D. J., Chen, J. & Houk, K. N. Theozymes and compuzymes: theoretical models for biological catalysis. *Curr Opin Chem Biol* **2**, 743–750 (1998).
95. Frushicheva, M. P. *et al.* Computer aided enzyme design and catalytic concepts. *Current Opinion in Chemical Biology* **21**, 56–62 (2014).
96. Acosta-Silva, C., Bertran, J., Branchadell, V. & Oliva, A. Kemp Elimination Reaction Catalyzed by Electric Fields. *ChemPhysChem* **21**, 295–306 (2020).
97. Huang, P.-S. *et al.* High thermodynamic stability of parametrically designed helical bundles. *Science* **346**, 481–485 (2014).
98. Nick Pace, C. & Martin Scholtz, J. A Helix Propensity Scale Based on Experimental Studies of Peptides and Proteins. *Biophysical Journal* **75**, 422–427 (1998).
99. Bhowmick, A. *et al.* Structural evidence for intermediates during O2 formation in photosystem II. *Nature* **617**, 629–636 (2023).
100. Cao, D. *et al.* Structure-based discovery of nonhallucinogenic psychedelic analogs. *Science* **375**, 403–411 (2022).
101. Yin, J. *et al.* Structure of a D2 dopamine receptor-G-protein complex in a lipid membrane. *Nature* **584**, 125–129 (2020).
102. Cherezov, V. *et al.* High-resolution crystal structure of an engineered human beta2-adrenergic G protein-coupled receptor. *Science* **318**, 1258–1265 (2007).
103. Choma, C. T. *et al.* Design of a heme-binding four-helix bundle. *J. Am. Chem. Soc.* **116**, 856–865 (1994).
104. Hutchins, G. H. *et al.* An expandable, modular de novo protein platform for precision redox engineering. *Proc Natl Acad Sci U S A* **120**, e2306046120 (2023).
105. Ennist, N. M., Stayrook, S. E., Dutton, P. L. & Moser, C. C. Rational design of photosynthetic reaction center protein maquettes. *Front Mol Biosci* **9**, 997295 (2022).
106. Mann, S. I. *et al.* De Novo Design of Proteins That Bind Naphthalenediimides, Powerful Photooxidants with Tunable Photophysical Properties. *J. Am. Chem. Soc.* **147**, 7849–7858 (2025).
107. Dunbrack, R. L. Rotamer libraries in the 21st century. *Curr Opin Struct Biol* **12**, 431–440 (2002).
108. Khersonsky, O. *et al.* Bridging the gaps in design methodologies by evolutionary optimization of the stability and proficiency of designed Kemp eliminase KE59. *Proceedings of the National Academy of Sciences* **109**, 10358–10363 (2012).
109. Collins, P. M. *et al.* Gentle, fast and effective crystal soaking by acoustic dispensing. *Acta Crystallogr D Struct Biol* **73**, 246–255 (2017).
110. Kabsch, W. XDS. *Acta Crystallogr D Biol Crystallogr* **66**, 125–132 (2010).
111. Evans, P. R. & Murshudov, G. N. How good are my data and what is the resolution? *Acta Crystallogr D Biol Crystallogr* **69**, 1204–1214 (2013).
112. McCoy, A. J. *et al.* Phaser crystallographic software. *J Appl Crystallogr* **40**, 658–674 (2007).
113. Liebschner, D. *et al.* Macromolecular structure determination using X-rays, neutrons and electrons: recent developments in Phenix. *Acta Crystallogr D Struct Biol* **75**, 861–877 (2019).

114. Emsley, P., Lohkamp, B., Scott, W. G. & Cowtan, K. Features and development of Coot. *Acta Crystallogr D Biol Crystallogr* **66**, 486–501 (2010).
115. Keegan, R., Wojdyr, M., Winter, G. & Ashton, A. *DIMPLE* : a difference map pipeline for the rapid screening of crystals on the beamline. *Acta Crystallogr A Found Adv* **71**, s18–s18 (2015).
116. Correy, G. J. *et al.* Extensive exploration of structure activity relationships for the SARS-CoV-2 macrodomain from shape-based fragment merging and active learning. 2024.08.25.609621 Preprint at https://doi.org/10.1101/2024.08.25.609621 (2024).
117. Winn, M. D. *et al.* Overview of the CCP4 suite and current developments. *Acta Crystallogr D Biol Crystallogr* **67**, 235–242 (2011).
118. Moriarty, N. W., Grosse-Kunstleve, R. W. & Adams, P. D. electronic Ligand Builder and Optimization Workbench (eLBOW): a tool for ligand coordinate and restraint generation. *Acta Crystallogr D Biol Crystallogr* **65**, 1074–1080 (2009).
119. Pearce, N. M., Krojer, T. & von Delft, F. Proper modelling of ligand binding requires an ensemble of bound and unbound states. *Acta Crystallogr D Struct Biol* **73**, 256–266 (2017).
120. Abramson, J. *et al.* Accurate structure prediction of biomolecular interactions with AlphaFold 3. *Nature* **630**, 493–500 (2024).
121. Alexandrova, A. N., Röthlisberger, D., Baker, D. & Jorgensen, W. L. Catalytic Mechanism and Performance of Computationally Designed Enzymes for Kemp Elimination. *J. Am. Chem. Soc.* **130**, 15907–15915 (2008).
122. Świderek, K., Tuñón, I., Moliner, V. & Bertran, J. Protein Flexibility and Preorganization in the Design of Enzymes. The Kemp Elimination Catalyzed by HG3.17. *ACS Catal.* **5**, 2587–2595 (2015).
123. Rakotoharisoa, R. V. *et al.* Design of Efficient Artificial Enzymes Using Crystallographically Enhanced Conformational Sampling. *J. Am. Chem. Soc.* **146**, 10001–10013 (2024).
124. Wang, S., Li, W., Liu, S. & Xu, J. RaptorX-Property: a web server for protein structure property prediction. *Nucleic Acids Res* **44**, W430–W435 (2016).
125. Case, D. A. *et al.* AmberTools. *J. Chem. Inf. Model.* **63**, 6183–6191 (2023).
126. Gaussian 09, Revision A.02 – ScienceOpen. https://www.scienceopen.com/document?vid=6be7271f-f651-464b-aee6-ef20b0743b6b.
127. Singh, U. C. & Kollman, P. A. An approach to computing electrostatic charges for molecules. *Journal of Computational Chemistry* **5**, 129–145 (1984).
128. Besler, B. H., Merz Jr., K. M. & Kollman, P. A. Atomic charges derived from semiempirical methods. *Journal of Computational Chemistry* **11**, 431–439 (1990).
129. Wang, J., Wang, W., Kollman, P. A. & Case, D. A. Automatic atom type and bond type perception in molecular mechanical calculations. *Journal of Molecular Graphics and Modelling* **25**, 247–260 (2006).
130. Wang, J., Wolf, R. M., Caldwell, J. W., Kollman, P. A. & Case, D. A. Development and testing of a general amber force field. *Journal of Computational Chemistry* **25**, 1157–1174 (2004).
131. Izadi, S., Anandakrishnan, R. & Onufriev, A. V. Building Water Models: A Different Approach. *J. Phys. Chem. Lett.* **5**, 3863–3871 (2014).
132. Settle: An analytical version of the SHAKE and RATTLE algorithm for rigid water models - Miyamoto - 1992 - Journal of Computational Chemistry - Wiley Online Library. https://onlinelibrary.wiley.com/doi/10.1002/jcc.540130805.
133. Darden, T., York, D. & Pedersen, L. Particle mesh Ewald: An N·log(N) method for Ewald sums in large systems. *The Journal of Chemical Physics* **98**, 10089–10092 (1993).
134. Gowers, R. J. *et al. MDAnalysis: A Python Package for the Rapid Analysis of Molecular Dynamics Simulations*. https://www.osti.gov/biblio/1565806 (2019) doi:10.25080/Majora-629e541a-00e.
135. Thorn, S. N., Daniels, R. G., Auditor, M. T. & Hilvert, D. Large rate accelerations in antibody catalysis by strategic use of haptenic charge. *Nature* **373**, 228–230 (1995).
136. Hollfelder, F., Kirby, A. J. & Tawfik, D. S. Off-the-shelf proteins that rival tailor-made antibodies as catalysts. *Nature* **383**, 60–63 (1996).
137. Khersonsky, O. *et al.* Optimization of the in-silico-designed kemp eliminase KE70 by computational design and directed evolution. *J Mol Biol* **407**, 391–412 (2011).
138. Merski, M. & Shoichet, B. K. Engineering a model protein cavity to catalyze the Kemp elimination. *Proceedings of the National Academy of Sciences* **109**, 16179–16183 (2012).
139. Gutierrez-Rus, L. I., Alcalde, M., Risso, V. A. & Sanchez-Ruiz, J. M. Efficient Base-Catalyzed Kemp Elimination in an Engineered Ancestral Enzyme. *Int J Mol Sci* **23**, 8934 (2022).
