# Supplementary material for: Emergence of binding and catalysis from a designed generalist binding protein
Source: bioRxiv. 2025 Jan 31:2025.01.30.635804. Preprint. [Version 1] doi: 10.1101/2025.01.30.635804 (PMC11838529; doi:10.1101/2025.01.30.635804)
Supplement: Supplementary file 1 [file NIHPP2025.01.30.635804v1-supplement-1.pdf]

bioRxiv preprint doi: https://doi.org/10.1101/2025.01.30.635804; this version posted January 31, 2025. The copyright holder for this preprint (which was not certified by peer review) is the author/funder, who has granted bioRxiv a license to display the preprint in perpetuity. It is made available under aCC-BY 4.0 International license.

1. Materials and Methods

1.1 ABLE expression and purification

Plasmid DNA encoding His_6-tagged ABLE with a Tobacco Etch Virus (TEV) protease cleavage site for tag removal (pET11a) was transformed into BL21 (DE3) *Escherichia coli* (*13*). After overnight growth at 37°C (LB-agar, 100 µg/ml carbenicillin), single colonies were used to inoculate small-scale cultures (10 ml LB, 100 µg/ml carbenicillin). Following overnight growth at 37°C, large-scale cultures (1 L LB, 100 µg/ml carbenicillin) were inoculated and grown at 37°C until an OD600 = 0.7, at which point protein expression was induced with 0.2 mM IPTG for 4 hours at 37°C. Cells were harvested by centrifugation (4000 g, 20 min, 4°C) and cell pellets were briefly washed with phosphate-buffered saline before being re-centrifuged (4000 g, 20 min, 4°C). The cell pellets were frozen at -80°C until further purification. Thawed cell pellets were resuspended in 25 ml lysis buffer (50 mM sodium phosphate pH 7.4, 150 mM NaCl, 20 mM imidazole) and 1/2 tablet of protease inhibitor (EDTA-free complete, Roche). Cells were lysed by sonication (2 min) and cell debris was collected by centrifugation (30,000 g, 30 min, 4°C). The supernatant was filtered (0.45 µm cellulose acetate) and incubated with Ni-NTA beads (pre-washed with lysis buffer) for 4 hrs at 4°C with gentle rolling. Beads were thoroughly washed with lysis buffer before protein was eluted from the beads with elution buffer (50 mM Tris pH 8.0, 100 mM NaCl, 0.5 mM EDTA, 1 mM DTT, 300 mM imidazole). Eluted protein was supplemented with TEV protease (mass ratio: 1:10 = TEV: ABLE) and dialyzed against TEV reaction buffer (50 mM Tris pH 8.0, 100 mM NaCl, 0.5 mM EDTA, 1 mM DTT) overnight at 4°C. After tag cleavage, the protein was incubated with Ni-NTA beads (pre-washed with TEV reaction buffer) for 2 hrs and the cleaved ABLE was eluted with TEV reaction buffer. The eluted fractions were concentrated to 4 ml and further purified via HPLC (C4 reverse phase column, 30-70% acetonitrile in water). Fractions containing protein were collected and lyophilized for 72 hrs. Lyophilized ABLE was stored at -80°C.

1.2 ABLE crystallization and fragment Screening

Lyophilized ABLE was dissolved in water to a concentration of 18 mg/ml. Crystals were grown using sitting drop vapor diffusion in SwissCI 3-well plates (HR3-125, Hampton) with 200 nl protein and 200 nl reservoir (220 mM sodium malonate pH 5, 20% PEG 3350 and 3% DMSO). Needle-shaped crystals grew overnight at 19°C. We screened the 320 compound Enamine Essential fragment library against ABLE. Fragment solutions (40 nl of 100 mM stocks prepared in DMSO) were added to crystal drops using an acoustic liquid handler (Echo 650, Beckman Coulter) (*82*). Crystals were incubated with fragments for 2-4 hours before being looped and vitrified in liquid nitrogen. X-ray diffraction data were collected at beamline 8.3.1 of the Advanced Light Source (ALS) and beamlines 12-1 and 12-2 of the Stanford Synchrotron Radiation Lightsource (SSRL). Data collection strategies are summarized in **Supplementary File 1**.

Diffraction images were indexed, integrated, and scaled with XDS and merged with Aimless(*83*, *84*). In total we mounted 447 crystals soaked with fragments and were able to obtain high quality datasets from 321 crystals (resolution limit <2.2 Å based on $CC_{1/2}$ < 0.3, R_{free} of initial model <35%). Replicate soaks were performed for some fragments: the total number of unique fragments with high-quality datasets was 242 (**Supplementary File 1**). We initially refined a model of ABLE against a dataset collected from a crystal soaked only in 10% DMSO. Phases were obtained by molecular replacement using Phaser (*85*) and chain A of the previously published ABLE X-ray crystal structure as the search model (PDB code 6W6X) (*13*). The initial model was improved by

bioRxiv preprint doi: https://doi.org/10.1101/2025.01.30.635804; this version posted January 31, 2025. The copyright holder for this preprint (which was not certified by peer review) is the author/funder, who has granted bioRxiv a license to display the preprint in perpetuity. It is made available under aCC-BY 4.0 International license.

iterative cycles of refinement with *phenix.refine* (version 1.21.1-5286) (*86*) and manual model building with COOT (*87*). Waters were placed automatically in early-stage refinement using *phenix.refine* into peaks in the F_O-F_C difference map >3.5 σ. In the later stages of refinement, waters were manually added/deleted, and hydrogens were refined with parameters constrained by those of the non-hydrogen atom (the riding hydrogen model). Data collection and refinement statistics are reported in **Supplementary File 1**. Coordinates and structure factor amplitudes were deposited in the PDB with accession code 9DW2. Refinement of the fragment-soaked datasets was performed using a pipeline based on Dimple as described previously using the DMSO-soaked ABLE model (PDB code 9DW2).(*88*, *89*) The pipeline consisted of initial rigid body refinement with refinement followed by two cycles of restrained refinement in Refmac: the first with harmonic distance restraints (jelly-body restraints, four cycles) and the second with restrained refinement (eight cycles).

Fragment binding was detected using the PanDDA algorithm (*90*) packaged in CCP4 version 7.0 (*91*). The background electron density map was calculated from 30 datasets (**Supplementary File 1**): six datasets were from crystals soaked in 10% DMSO and the remaining datasets came from fragment-soaked crystals where no fragment was detected. We collected multiple datasets for selected fragments. If fragments were detected in multiple datasets, we modeled the fragment with the highest occupancy based on the PanDDA event map and 1-BDC (Background Density Correction) value. Fragments were modeled into PanDDA event maps (**Supplementary File 1**) using COOT (version 0.8.9.2) (*87*) and changes in protein conformation and solvent near the fragments were modeled. Fragment restraint files were generated with *phenix.elbow* (*92*) from 3D coordinates generated by LigPrep (Schrödinger, version 2022-1) or Grade 2 (Global Phasing Ltd.). Structure refinement was performed with *phenix.refine* (version 1.20.1-4487) starting from the ABLE DMSO reference structure (PDB code 9DW2). Alternative conformations were modeled for residues when the RMSD exceeded 0.15 Å from the apo structure. The apo conformation was assigned alternative location (altloc) A and the fragment-bound conformation was assigned altloc B. Two fragments bound with alternative conformations that overlapped with the Tyr46 sidechain (PDB code 7HJK and 7HJX). For these fragments, residues 44-48 were modeled with a second conformation (altloc C). The multi-state models were initially refined with five *phenix.refine* macrocycles without hydrogens, followed by 10 macrocycles with riding hydrogens. Occupancy of the fragment- and apo-states was refined at either 2*(1-BDC) (*90*, *93*) or at fragment occupancy values from 10-90% at 10% increments. Fragment occupancy was determined by inspection of mF_O-DF_C difference map peaks after refinement. The occupancy at 2*(1-BDC) was appropriate for 41/43 fragments (**Supplementary File 1**). Coordinates and structure factor amplitudes have been deposited in the PDB with accession codes 7HIY, 7HIZ, 7HJ0, 7HJ1, 7HJ2, 7HJ3, 7HJ4, 7HJ5, 7HJ6, 7HJ7, 7HJ8, 7HJ9, 7HJA, 7HJB, 7HJC, 7HJD, 7HJE, 7HJF, 7HJG, 7HJH, 7HJI, 7HJJ, 7HJK, 7HJL, 7HJM, 7HJN, 7HJO, 7HJP, 7HJQ, 7HJR, 7HJS, 7HJT, 7HJU, 7HJV, 7HJW, 7HJX, 7HJY, 7HJZ, 7HK0, 7HK1, 7HK2, 7HK3 and 7HK4. Structure factor intensities (unmerged, merged, and merged/scaled), PanDDA input and output files including Z-map and event maps in CCP4 format, and refined models including the fragment-bound state extracted from multi-state models were uploaded to Zenodo (DOI: 10.5281/zenodo.13913848).

1.3 Computational design of fluorescent ABLE (FABLE)

With (7-hydroxylcoumarin)-ABLE structure (PDB: 7HIY) available from fragment-screening, we superimposed Cou485 to 7-hydroxycoumarin fragment based on shared heavy atoms to obtain the

bioRxiv preprint doi: https://doi.org/10.1101/2025.01.30.635804; this version posted January 31, 2025. The copyright holder for this preprint (which was not certified by peer review) is the author/funder, who has granted bioRxiv a license to display the preprint in perpetuity. It is made available under aCC-BY 4.0 International license.

initial pose, which contains the Tyr46 packing with 2H-1-benzopyran ring of coumarin and hydrogen bond between His49 and ketone of coumarin. This initial pose was used for Rosetta flexible backbone sequence design, using a previously reported script in our lab. (*44*) (Supplement text, Computational design of FABLE) During the sequence design, the side chain conformation of His49 and Tyr46 were fixed and the geometry constraint for the His49 interaction with ketone of Cou485 was applied. Out of the total 126 residues, 43 residues in the vicinity of apixaban and 7-hydroxylcoumarin binding site were chosen to be designed to reshape the binding pocket towards Cou485 while keeping the portion of the packing core of ABLE that is not involved in binding ligands unchanged. We selected the 100 best-scoring designs (lowest Rosetta energy score, using the Rosetta Ref2015 energy function) from 1000 design outputs. We next carried out structure prediction using OmegaFold (*55*) and ESMFold (*49*) to further filter out the designs whose predicted structures do not agree with the designed structures. Sequences with a less than 1.0 Å RMSD difference between the design model and prediction as well as a plDDT score over 90 were picked for detailed structural evaluation. Final sequences were selected by additional criteria: 1) No more than 2 unsatisfied side-chain hydrogen bonds in the interior of design; 2) Pre-organized binding pocket from the prediction, especially the side-chain conformation of Tyr46 and His49, which is quantified by the sub-Å RMSD of CA, CB, CG between the designed model and AF3 prediction. AF3 server (https://alphafoldserver.com) was used to predict the structures for selected designs, as indicated, although the program was not available at the time that the sequences were designed.(*48*)

1.4 Expression and purification for designed FABLE proteins

The designed FABLE proteins, with additional N-terminal His tag and TEV cleavage site, were subjected to codon optimization for *E. Coli.* using Integrated DNA Technologies (IDT) codon optimization tool. The optimized genes with additional sequences of (GTTTAACTTTAAGAAGGAGATATACAT) at 5' and (CTCGAGTGAGATCCGGCTGCTAAC) at 3' of gene were ordered as "clonal genes" from Twist Biosciences, which were assembled into pET29b vector digested with Nde1 and Xho1 using Gibbson assembly (*94*).

The plasmids were introduced into *Escherichia coli* BL21(DE3) cells (C2527H, New England Biolabs) via chemical transformation. The transformed cells were cultured in a 5 mL starter culture of Luria-Bertani (LB) medium supplemented with 50 μg ml^{-1} kanamycin at a temperature of 37°C. The starter culture was used for protein expression at 1 L LB supplemented with 50 μg ml^{-1} kanamycin. Protein expression was induced with 1 mM isopropyl β-D-1-thiogalactopyranoside (IPTG) (Apex Bioresearch Products) when the optical density at 600 nm (A_{600}) reached 0.8. After overnight expression at 30°C, the pellets were harvested by centrifugation, and their pellets were stored at -80°C.

For protein extraction, the cell pellets were resuspended in PBS buffer (119-069-13, Quality Biological) and lysed via sonication. The resulting cell lysates were clarified by centrifugation at 14,000 g for 30 min. The supernatants after centrifugation were purified with nickel-nitrilotriacetic acid (Ni-NTA) resin (Invitrogen), following the manufacturer's instruction.

After buffer exchange to PBS using PD-10 column (17085101, Cytiva), proteins were analyzed using sodium dodecyl-sulfate polyacrylamide gel electrophoresis (SDS-PAGE) and fast protein liquid chromatography (FPLC).

1.5 Screening fluorophore library with FABLE

bioRxiv preprint doi: https://doi.org/10.1101/2025.01.30.635804; this version posted January 31, 2025. The copyright holder for this preprint (which was not certified by peer review) is the author/funder, who has granted bioRxiv a license to display the preprint in perpetuity. It is made available under aCC-BY 4.0 International license.

Fluorophores dissolved in DMSO were added to FABLE in PBS to a final concentration of 38 µM FABLE and 50 µM coumarin fluorophores, including A1 to D4, whose individual structures are summarized in **Table S3**. Besides fluorophores with coumarin scaffolds, we also evaluated the interaction of FABLE with other environment-sensitive fluorophores, including L036 to TMR. Those fluorophores dissolved in DMSO were added to FABLE in PBS to a final concentration of 38 µM FABLE and 1 µM fluorophore. The fluorescence of each fluorephore was recorded with BioTek Synergy Neo2 Reader after incubating at room temperature for 30 minutes. The exact wavelength of excitation and emission for these fluorephores are in **Table S3**.

1.6 FPLC analysis

To confirm the coumarin dyes were firmly bound to the FABLE, we prepared the FABLE-coumain complexes and showed that the complexes were stable during chromatographic separation. To prepare the ligand-FABLE complex, 100 µM FABLE was mixed with 200 µM of Cou485, Cou481, and Cou540a, respectively. After incubation at room temperature for 30 min, the resulting complex was injected to the Fast Protein Liquid Chromatography (FPLC) equipped with a Superdex 75 Increase 5/150 analytical column. The ligand was monitored by their absorbance indicated in the plot (**fig. S12**).

1.7 Determining binding constants

We used fluorescence titration experiments to determine the binding dissociation constants of FABLE for Cou485, Cou481, and Cou540a with BioTek Synergy Neo2 Reader.
The excitation and emission wavelength with the largest shift upon FABLE interaction was used to quantify the the bound ligand, which is 395 nm (excitation) and 470 nm (emission) for Cou485, 410 nm (excitation) and 470 nm (emission) for Cou481, 440 nm (excitation) and 510 nm (emission) for Cou540a. For each ligand, FABLE in PBS was serially diluted while fixing the concentration of ligand at indicated values. Global fitting with 2 fixed ligand concentrations was used to fit K_D following the single-site binding model (Equation 1, N=1, L=indicated concentration, shared K_D) using GraphPad Prism. b_{free} was obtained by measuring the fluorescence of the ligand in PBS after serial dilution.

Equation 1

$$Fluorescence = b_{bound}[Ligand_{bound}] + b_{free}[Ligand_{free}]$$

- $Ligand_{total:}$ total concentration of ligand
- $Ligand_{free:}$ free ligand
- $Ligand_{bound}$: ligand binding to protein
- $Ligand_{free} = ligand_{total} - ligand_{bound}$
- b_{free} = brightness (fluorescence) of free ligand per µM
- b_{bound} = brightness (fluorescence) of bound ligand per µM

Equation 2

bound ligand =

$$\frac{1}{2}\left[K_D[Ligand_{total}] \frac{[Protein_{total}]}{N} \sqrt{\left(K_D + [Ligand_{total}] + \frac{[Protein_{total}]}{N}\right)^2 - 4\,[Ligand_{total}]\frac{[Protein_{total}]}{N}}\;\right]$$

- $Protein_{total}$: total protein concentration
- N: binding stoichiometry

bioRxiv preprint doi: https://doi.org/10.1101/2025.01.30.635804; this version posted January 31, 2025. The copyright holder for this preprint (which was not certified by peer review) is the author/funder, who has granted bioRxiv a license to display the preprint in perpetuity. It is made available under aCC-BY 4.0 International license.

- K_D: binding constant of protein-ligand interation

1.8 FABLE crystallization and structure determination

FABLE crystals were grown by sitting drop vapor diffusion using SwissCI 3-well crystallization plates with reservoir solutions containing: i) 100 mM Tris pH 8.0, 20% (v/v) MPD, ii) 200 mM sodium chloride, 100 mM CHES pH 9.5, 50% (v/v) PEG 400 or iii) 100 mM CHES pH 9.5, 30% (v/v) PEG 600. Crystal drops were set up with 200 nl reservoir and 200 nl protein. Crystals were looped and vitrified in liquid nitrogen without further cryoprotection. X-ray diffraction data were collected at SSRL beamline 12-2 (MPD condition) or ALS beamline 8.3.1 (PEG 400 and PEG 600 condition) with the strategy and data collection statistics summarized in **Supplementary File 1**. Data were reduced and models refined using the same procedure as described for ABLE, except phases were obtained for the MPD structure using molecular replacement and the previously published ABLE structure (PDB code 6W6X), while phases for the PEG 400 and PEG 600 structures were obtained using the structure from the MPD condition (PDB code 9DWA). Refinement statistics are reported in Table S1 and coordinates and structure factor amplitudes have been deposited in the PDB with accession codes 9DWA (MPD condition), 9DWB (PEG 400 condition) and 9DWC (PEG 600 condition).

1.9 Molecular dynamics (MD) simulation

1.9.1 General MD procedure

Proteins and ligands were parameterized using Gaussian09 and AmberTools's Antechamber program in Amber22(*95*, *96*). Ligand Partial charges for ligand were determined by first optimizing its geometries at the B3LYP/6-31G* level (*96*), and then calculating its electrostatic potentials using the Merz-Singh-Kollman method in Gaussian09 (*96*). Charge fitting was then performed using Antechamber's RESP program within AmberTools (*97*, *98*). All other small molecule parameters were assigned by Antechamber based on the GAFF2 database (*99*, *100*). The starting structures for the MD simulations are described in the following section. The simulation box was built by solvating protein with OPC-modeled waters (*101*) in a box with 8 Å padding from the protein, and sodium and chloride ions were added to reach a charge-neutral 137 mM NaCl concentration to match experimental conditions. Simulations began with 1,000 restrained steepest-descent minimization steps before switching to a maximum of 6,000 steps in conjugate gradient steps. The system was then heated up to 278 K over 50 ps in the NVT ensemble with Langevin thermostat control of temperature and 1 fs timestep. The simulation was then switched to the NPT ensemble, and pressure was maintained at 1 atm using the Monte Carlo barostat (*102*). Throughout equilibration steps, protein and ligand heavy atoms were initially restrained with harmonic potentials at 10 kcal/(mol·Å2) and ramped down to 0 kcal/(mol·Å2) over 9 equilibration steps, totaling 1 nanosecond. Each simulation was then carried out for an unrestrained production run under periodic boundary conditions with 2 fs timesteps. The SHAKE algorithm (*103*, *104*) was used to restrain hydrogens, short-range non-bonded electrostatic and Lennard-Jones interactions were cut off at 10 Å, and the Particle Mesh Ewald method (Ref*X13*) was used for long-range electrostatics.

1.9.2 MD for ABLE

The starting structures for the simulations were derived from the crystal structures of ABLE complexed with apixaban (PDB 6W70, chain B), and apo ABLE (PDB 6W6X, chain A) (*13*). Both

bioRxiv preprint doi: https://doi.org/10.1101/2025.01.30.635804; this version posted January 31, 2025. The copyright holder for this preprint (which was not certified by peer review) is the author/funder, who has granted bioRxiv a license to display the preprint in perpetuity. It is made available under aCC-BY 4.0 International license.

systems were prepared using the epsilon nitrogen-protonated state of H49 ("HIE"). The simulation was carried out at 278K for 2 independent runs with each run of 500 ns. The final trajectory was extracted with a 5 ns interval, which resulted in 100 states for each run of 500 ns. Chi1 and Chi2 angles of Tyr46 were extracted with an in-house script (**extract_chi1_chi2.py, supplemental text**) and manual inspection was also done to verify the calculation of Chi2 angle due to the symmetry of tyrosine sidechain. Chi1 and Chi2 of His49 were extracted with another script (**extract _chi1_chi2.py, supplemental text**). Contours were generated from the chi1-chi2 angles seen at the simulation using an in-house script (**generate_contour_from_tyr_chi1and2.py, supplemental text**) which utilizes the Gaussian kernel density estimate methods from scipy.stats module of SciPy Python Packages.

(1) MD for KABLE

The starting structures for the KABLE1simulations were derived from the design structures of KABLE1, in complex of 6NBT. For the simulation of KABLE1.4 with four mutations, Rosetta FastRelax was used to incorporate these mutations and the relaxed structures were used for MD simulation. The simulation was carried out at 300 K for 3 independent runs with Berendsen barostat and each run lasted for 1000 ns. The final trajectory was extracted with 1 ns intervals, which resulted in 1000 states for each run of 1000 ns. Chi1 and Chi2 of Gln75 and Leu79 were extracted using the script. (extract _chi1_chi2.py) The number of waters within 3.5 Å of 6NBT was calculated using the script (**count_water.py, supplemental text**)

1.10 Thermal stability

Proteins were prepared in 10 μM concentrations in PBS buffer. CD spectra were collected on a Jasco J-810 CD spectrometer in a 0.1 cm path-length quartz cuvette. The parameters for full spectra collections were set up as follows: the bandwidth was set to 2 nm, the scanning speed was set to 50 nm/min, and the average of accumulations was set to 3. Temperature-dependent data were collected at 222 nm from 20 to 95 °C with an interval of 5 °C and an increase rate of 2 °C/minute.

1.11 Computational design of KABLE (Kemp Eliminase)

Our goal was to use the information from the fragment complexes to inform the design of the active site. As the substrate was a bicyclic aromatic compound, it was logical to position it into the aromatic boxes of the A site, hosting 8 bicyclic fragments. This was followed by searching for a Glu or Asp sidechain that was well positioned to abstract a proton from the substrate to catalyze the elimination reaction (Strategy 1). The second strategy centered instead on using the information from fragment complexes to first identify a catalytically favorable position for a Glu/Asp transition state complex, followed by redesign of ABLE's binding site to stabilize the bicyclic ring, primarily through hydrophobic, aromatic interactions and van der Waals interactions.

In each case we considered theoretical suggestions that emphasize the location of a binding site relative bulk solvent. As early as 1967 Perutz stated: "The non-polar interior of enzymes provides the living cell with the equivalent of the organic solvents used by the chemists. The substrate may be drawn into a medium of low dielectric constant in which strong electric interactions between it and specific polar groups of the enzyme can occur." Thus, it is important to consider placement of the base in a pre-organized, solvent-inaccessible location well below the surface of the protein. This principle has been supported by a body of theoretical and computational studies, as well as through careful examination of evolved Kemp eliminases.(*30*, *32*, *33*, *70*) We satisfied Perutz's this principle by choosing designs in which the carboxylate base and the substrate were fully

bioRxiv preprint doi: https://doi.org/10.1101/2025.01.30.635804; this version posted January 31, 2025. The copyright holder for this preprint (which was not certified by peer review) is the author/funder, who has granted bioRxiv a license to display the preprint in perpetuity. It is made available under aCC-BY 4.0 International license.

inaccessible to a water-sized probe, the surrounding sidechains should be primarily hydrophobic (*32*, *61*) and the geometry of the carboxylate should be close to the saddle point for the computed transition state of the reaction.

1.11.1 Strategy 1: Aromatic box

Inspired by the aromatic boxes of conformer A, we used a similar procedure for design of a Kemp Eliminase. For the aromatic box in conformer A, we used 7HJQ as the starting structure. The plane of the ring of 6NBT was oriented as in 7HK4. The next step was to identify a position for incorporation of the active site Asp or Glu to satisfy the following criteria: **1)** When in a low-energy rotamer, its carboxylate should form a hydrogen bond to N3 of 6-nitrobenzotriazole (6NBT) in a geometry computed from previous quantum calculations (*29*, *105*, *106*) **2)** The benzisoxazole ring of the substrate should be fully buried (to increase the basicity of the carboxylate). Thus, the Asp/Glu carboxylate should be solvent-inaccessible. We also filtered out any examples where the C-alpha of the acidic residue is located at a surface position, but the sidechain reaches into the protein interior, because the burial of the carboxylate would need to compete with solvation. **3)** The introduced sidechain should not clash with the backbone or sidechains of the residues comprising the aromatic box. This left 3 positions to explore, namely 108, 112 and 115. After searching through low-energy sidechain rotamers of Asp and Glu at these locations, Glu at position 108 was found to uniquely fulfill these criteria. The resulting complex was used as an input structure for sequence design with either an 1) an iterative round of LigandMPNN (*47*) and Rosetta FastRelax (*54*) or a 2) classical flexible backbone Rosetta sequence design (*54*).

1) For iterative rounds of LigandMPNN and Rosetta FastRelax route, residue 46 was fixed to either Y or W and residue 86 was fixed to F to maintain the aromatic packing interactions discovered in fragment screening. In the Rosetta FastRelax step, geometric constraints from the reported crystallographic conformation sampling were applied (**supplementary text, constrain for active site 1 of kemp eliminase**).(*107*)
2) The second sequence design of the flexible backbone Rosetta sequence design proceeded with the same constraint applied (**supplementary text, constrain for active site 1 of kemp eliminase**) following the reported protocol from our lab (*44*).

Structure prediction with RaptorX was carried out for all designed sequences (*67*, *108*), to ensure that the designed structures would be preorganized to adopt the same active site geometry of the design model. Of 2000 sequences evaluated on 1), we chose designs (1, 4 and 5). Of 2000 sequences evaluated on 2), we chose designs 2 and 3. The selection criteria were the sub-Å backbone RMSD between designed complex and the predicted unliganded structures and also agreement of sidechain conformation of active site (108, 46, 79, 86) between design and prediction.

1.11.2 Strategy 2: Hotspot residue 49

In strategy 2 we focused on identifying a site that was well-positioned to accommodate a Glu/Asp as the general base. His49 was selected as a potentially ideal position, because it was situated at a central location near the bottom of the binding site where it donated a hydrogen bond to a carbonyl of apixaban in ABLE. His49 also formed hydrogen bonds to 35% fragments binding at the pocket of ABLE, and it formed imidazole-carboxylate salt bridges in 9 of the complexes, as well as with acetate in 6W6X (**fig. S13**). We reasoned that it should be possible to reverse the polarity of the interaction by replacing the His with an Asp, which is nearly isosteric to histidine, as this would place the sidechain in a good environment to form a strong hydrogen bond. 6NBT was docked to

bioRxiv preprint doi: https://doi.org/10.1101/2025.01.30.635804; this version posted January 31, 2025. The copyright holder for this preprint (which was not certified by peer review) is the author/funder, who has granted bioRxiv a license to display the preprint in perpetuity. It is made available under aCC-BY 4.0 International license.

the Asp49 of ABLE-fragment crystal structures (PDB: 7HJV) based on previously reported geometric constraints from DFT calculations, considering the more basic (trans) lone pairs of electrons on either OD1 or OD2 for proton abstraction(*29*).
All 3 resulting poses (**fig. S13**) were used as input structures for sequence design with 3 iterative rounds of LigandMPNN and Rosetta FastRelax, resulting in 1000 designs per input pose. In the Rosetta FastRelax step, geometric constraints from the reported crystallographic conformation sampling were applied (**supplementary text, constrain for versatile residue 49 of kemp eliminase**) (*107*). Structure prediction with RaptorX was carried out for all designed sequences (*67*, *108*). Five designs were picked based on the goodness of fit between the predicted model of the uncomplexed structure versus the design model for the complex as described in the section above.

1.12 Library design

The plasmid encoding the KABLE1 gene was used as the template for site-specific saturation mutagenesis, which was achieved using megaprimer PCR protocol with primer sets (Integrated DNA Technologies) that overlapped the 5' position of the randomized position, together with a flanking primer (T7 reverse). Saturation mutagenesis was performed using NNK codons (where N can be any base and K represents a mixture of G and T nucleotides) that cover the 20 genetically encoded amino acids (primer sequences were shown in **table S9**). There are 20 libraries with NNK codon at indicated residue (**table S9**). The size of the PCR product was checked using agarose gel (1%) electrophoresis. Product DNA was digested with DpnI (New England Biolabs) at 25 °C for 12-14 hours to eliminate the parental clone. The sample was transformed into *E. coli* NEB5α cells (C2987H, New England Biolabs) and plated on an LB agar plate containing kanamycin (50 μg ml^{-1}). Colonies obtained from the plate upon incubation at 37 °C for 12-14 hours were grown in LB media with kanamycin at 37 °C for 2-3 hours. Cells were collected by centrifugation at 6,500*g* for 10 min. Plasmids were extracted using a DNA plasmid extraction kit following the manufacturer's protocol (Monarch, New England Biolabs). Library quality was confirmed by Sanger sequencing (Azenta Life Sciences).

1.13 Library screening

Around 160 randomly picked independent colonies were screened for each library. The plasmid encoding the NNK library was transformed into *E. coli* BL21 cells and plated on LB agar containing kanamycin. Individual colonies were inoculated in LB media (200 μl) containing kanamycin in a 96-well plate. Cultures were incubated at 37 °C, 220 rpm for 5-6 hours. A replica deep 96-well plate (Costar) was generated, where cultures (20 μl) were inoculated in LB media (400 μl) containing kanamycin and IPTG (0.5 mM). Cultures were then allowed to grow at 30 °C, 220 rpm for 20 h. Cells were collected by centrifugation at 4 °C, 2,000*g*. Pellets were resuspended in buffer A (25 mM Tris, pH 8.0, 20 mM imidazole, 300 mM NaCl), centrifuged again, and the supernatant was discarded. Cells were lysed using a buffer containing 25 mM Tris, pH 8.0, 100 mM NaCl, 0.5% triton X and centrifuged to separate the supernatant. Kemp eliminase activity of the clones was tested using a 96-well plate in the buffer (20 mM Tris, pH 8.0, 100 mM NaCl) containing 1.5% acetonitrile. Reactions were initiated upon mixing cell lysate with 100 μM substrate in a 1:1 ratio (final substrate concentration of 50 μM). Product formation was observed by measuring absorbance at 380 nm at 25 °C on a plate reader for 10 min. The activities of the clones showing notable improvement in activity over the initial design were confirmed by rescreening them in triplicate. Plasmids extracted from the colonies demonstrating enhanced

bioRxiv preprint doi: https://doi.org/10.1101/2025.01.30.635804; this version posted January 31, 2025. The copyright holder for this preprint (which was not certified by peer review) is the author/funder, who has granted bioRxiv a license to display the preprint in perpetuity. It is made available under aCC-BY 4.0 International license.

activity were sequenced (Azenta Life Sciences) to determine the identities of beneficial mutations. Sequences of the design (KABLE1) as well as the final quadruple mutant (KABLE1.4) are provided in **table S10**.

1.14 Expression and purification of KABLEs

The plasmids encoding KABLE0, KABLE1, and its mutants, cloned into pET29b(+) vector with NdeI and XhoI restriction sites, were transformed into *E. coli* BL21(DE3) (C2527H, New England Biolabs) and plated on LB agar plate with 50 µg ml^{-1} kanamycin (Thermo Scientific). This same kanamycin concentration was used throughout the experiment. Single colonies were inoculated into 15 mL LB medium containing kanamycin and grown at 37 °C, 220 rpm for 5-6 hours. 10 ml of starter culture was then diluted into 1 L of LB with kanamycin and allowed to grow at 37 °C until A_{600} reached 0.6-0.8. The culture was induced upon the addition of 0.5 mM isopropyl-ß-D-1-thiogalactopyranoside (IPTG) (Apex Bioresearch Products) and grown at 30 °C for 20 hours. Cells were harvested by centrifugation (Sorvall Legend RT+, Thermo Scientific) at 4 °C, 4, 3500 rpm for 15 min., flash frozen in liquid nitrogen, and stored at -80 °C.

For protein purification, 200 mL cells were resuspended in 40 mL buffer A (25 mM Tris, pH 8.0, 20 mM imidazole, 300 mM NaCl). Cell lysis was done upon sonication (Sonic Dismembrator Model 500, Fisher Scientific) for 10 min. (20 s pulse, 20 s rest) at an amplitude of 30%. To isolate the soluble fraction, centrifugation was performed at 4 °C, 20,000*g* for 30 min. The lysate was loaded onto a Ni-NTA gravity column (Hispur, Thermo Scientific) pre-equilibrated with buffer A, washed, and eluted using buffer B (25 mM Tris, pH 8.0, 250 mM imidazole, 300 mM NaCl). Protein fractions identified by Bradford assay were combined, concentrated to 3 ml using 10K MWCO spin concentrator (Amicon Ultra, Millipore Sigma), and exchanged into TEV cleavage buffer (50 mM Tris, pH 8.0, 75 mM NaCl) using desalting column (BioRad, 10 DG). TEV protease (at protein-to-protease absorbance (A_{280}) ratio of 100:1) along with EDTA (0.5 mM) and DTT (1 mM) was added to cleave the N-terminal His_6-tag. Protein was incubated at 37 °C for 12-14 hours and exchanged into buffer C (20 mM HEPES, pH 7.0, 100 mM NaCl). Protein fractions were then passed through Ni-NTA column to remove the protease and any un-cleaved KABLE protein. Final purification was performed on GE Healthcare AKTA FPLC system with Superdex 75 Increase 10/300 column in buffer C. Protein concentrations were determined by measuring the absorbance at 280 nm using extinction coefficient determined by Expasy ProtParam tool (https://web.expasy.org/protparam/).

1.15 Kinetic characterization

The initial rates for Kemp elimination for all the kinetic assays were corrected for the background rates in the appropriate buffers without the enzyme. Product (2-hydroxy-5-nitro-benzonitrile) concentration was determined using an extinction coefficient of 15,800 $M^{-1}cm^{-1}$ at 380 nm. The individual kinetic parameters (k_{cat}, K_M, and pK_a) were determined by globally fitting the dependence of initial rates on both substrate concentration and assay pH. Substrate 5-nitrobenzisoxazole (5NBI, AK Scientific) concentration ranging from 100 µM to 1 mM and assay pH ranging from pH 6 to pH 10 were used. The substrate 5NBI was prepared in acetonitrile as 100 mM stock, which was further diluted in buffer to prepare working solutions of various substrate concentrations at each tested pH. To maintain the pH at different values, different buffers were used at 20 mM in the presence of 100 mM NaCl (MES at pH 6.0 and 6.5, HEPES at pH 7.0 and 7.5, Tris at pH 8.0 and 8.5, CHES at pH 9.0 and 9.5, and CAPS at pH 10.0). The reactions were initiated by mixing the enzyme in buffer and solutions of 5NBI in a 1:3 ratio in a 96-well plate

bioRxiv preprint doi: https://doi.org/10.1101/2025.01.30.635804; this version posted January 31, 2025. The copyright holder for this preprint (which was not certified by peer review) is the author/funder, who has granted bioRxiv a license to display the preprint in perpetuity. It is made available under aCC-BY 4.0 International license.

(Genesee Scientific). The final reaction mixtures contained enzyme (5-1000 nM, depending on the activity of the mutant) in buffer, 1.5% acetonitrile (to maintain the constant final concentrations of the substrate), and variable concentrations of substrate at its pH-specific buffer. Product formation was monitored on a plate reader (BioTek Synergy neo2, Serial: 22052011) at 25 °C for 10 min. with the minimum kinetic interval determined by manufacturer provided software (Gen 5, version 3.12).

For KABLE family of proteins, its k_{cat}, K_M, and pK_a were fitted based on equation 3 where a pH-titratable catalytic base was considered in the reaction process. For a given protein, its initial velocities from a series of substrate concentrations and assay pHs were used to fit a shared set of k_{cat}, K_M, and pK_a value for all these data using GraphPad Prism 9.3.1. A pH range covering the pKa $\pm$ 2 was used for each KABLE variant. No pH higher than pH 10 was used because of the increased background decomposition of 5NBI. To capture the initial velocity of KABLEs, less enzyme concentration was used for ones at higher pH and more active ones as summarized in **Table S5**. The data conform well to equation 3, and the least squares fit returned unique values of k_{cat}, K_M, and pK_a for all tested proteins, except for KABLE1$_{K14P}$, KABLE1.2, KABLE1.3, and KABLE1.4. Although the values of k_{cat}/K_M were well-defined for these proteins, there was some covariation of the values for k_{cat} and K_M. Therefore, a single parameter (k_{cat}) was held constant, allowing the value of the pK_a and K_M to vary. A range of fixed k_{cat} was tested to find the k_{cat} allowing the maximal R-square > 0.99). As in **fig. S19**, the reported k_{cat} enables the best fit (maximal R-square) across a wide range of tested k_{cat}, and the R-square decreases as the fixed k_{cat} moves away from the reported value. The value of K_M and pK_a were directly taken from the fitting with the reported k_{cat} held a constant based on the R-square analysis. For this set of KABLEs, its standard error of mean of k_{cat} was obtained by taking the highest ratio of standard error of mean to mean of the fitted k_{cat} among all the tested enzymes, considering that there may be more error for the values of the of k_{cat} from R-square analysis.

Besides globally fitting kinetic paramters and pK_a, we also fit the kinetic parametrs and pK_a using the individual kinetic parameters fitted at each pH as in reference (*60*) for KABLE1 and its most active variant, KABLE1.4. Specifically, for each pH, the initial rate, under a series of substrate concentrations, was used to fit the k_{cat} and K_M using the Michaelis-Menton equation in GraphPad Prism. The dependence of k_{cat}/K_M and k_{cat} on pH was fitted using the equation $k_{cat}/K_M = (k_{cat}/K_M)_{EH} \times (1\text{-}f_{E\text{-}}) + (k_{cat}/K_M)_{E\text{-}} \times f_{E\text{-}}$, in which $f_{E\text{-}}$ is the fraction of the protein in the anionic state and is related to pH by: $f_{E\text{-}}=1/(1+10^{pKa\text{-}pH})$. Similarly, $k_{cat} = (k_{cat})_{EH} \times (1\text{-}f_{E\text{-}}) + (k_{cat})_{E\text{-}} \times f_{E\text{-}}$.

The values of $(k_{cat}/K_M)_{E\text{-}}$, $(k_{cat})_{E\text{-}}$ and pK_a are indicated in **fig. S17**. The values of $(k_{cat}/K_M)_{E\text{-}}$ and pK_a from this treatment agree with the k_{cat}/K_M from global fitting of all the variable substrate and pH data. The value of $(k_{cat})_{E\text{-}}$ is aound 3 times lower than the value obtained from global fitting (the values of K_M covaried, to keep k_{cat}/K_M constant). Considering the co-varibility of k_{cat} and K_M at individual pH, we used global fitting as our primary method to report kinetics parameters and pK_a.

Reaction process of Kemp elimination:

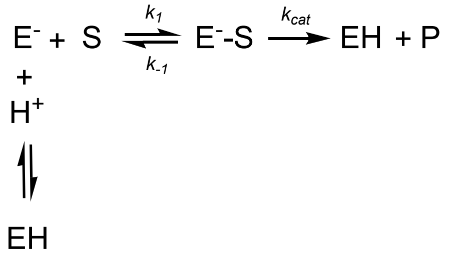

bioRxiv preprint doi: https://doi.org/10.1101/2025.01.30.635804; this version posted January 31, 2025. The copyright holder for this preprint (which was not certified by peer review) is the author/funder, who has granted bioRxiv a license to display the preprint in perpetuity. It is made available under aCC-BY 4.0 International license.

From Classical Michaelis-Menten Equation: $K_M = \frac{k_{-1}+k_{cat}}{k_1}$ $V_0 = \frac{k_{cat} \cdot [E^-] \cdot S}{K_M+S}$

Incorporation of pH-titratable base: $K_a = \frac{[E^-][H^+]}{EH}$ $E_t = E^- + E^-\text{-}S + EH$

Equation 3

Final Expression of V_0:

$$V_o = \frac{k_{cat} \cdot E_t \cdot S}{(K_M+S)(1+\frac{S}{K_M}+10^{(pKa-pH)})} + c$$

- Et is the total concentration of enzyme.
- pK_a is the pK_a of the titratable catalytic base.
- c is the baseline correction.

1.16 KABLE crystallization and structure determination

LigandMPNN chose very polar and flexible surface residues for KABLE, which impeded crystallization. To improve the crystallization of KABLE1.2, we introduced 23 surface mutations to make KABLE12$_{cryst}$. Because ABLE crystallizes easily, we transplanted its surface residues to KABLE12$_{cryst}$ as indicated in **fig. S21**. Following the previous expression and purification procedures, both the histag-cleaved version and histag-uncleaved version at PBS buffer were used to screen crystals and prepare the complex with 6NBT. 100 mM 6NBT dissolved in DMSO was added to 2.5 mM KABLE12$_{cryst}$ for the final 6NBT concentration of 10 mM.
Apo crystals of KABLE12$_{cryst}$ with an intact His-tag were grown by sitting-drop vapor diffusion using SwissCI 2-well crystallization plates with reservoir solution containing 0.2 M sodium bromide, 0.1 M Bis-Tris propane pH 6.5, 20% PEG 3350. Crystals of KABLE12$_{cryst}$ in complex with 6NBT grew using His-tag cleaved protein from a reservoir containing 0.2 M zinc acetate, 0.1 M sodium cacodylate pH 6.5, 18% PEG 8000. Crystals were looped and vitrified in liquid nitrogen without further cryoprotection. X-ray diffraction data were collected at ALS beamline 8.3.1 with the strategy and data collection statistics summarized in **Supplementary File 1**. Data were reduced and models refined using the same procedure as described for ABLE. For the structure in complex with 6NBT, ligand restraints were generated with *phenix.elbow* (for 6NBT) and Grade2 (for cacodylate) (*92*). In addition to the 6NBT molecule present in the KABLE core, two additional 6NBT molecules were identified in complex with three zinc molecules and a cacodylate ion at a crystal packing interface (**fig. S28**). The metal identity was supported by the calculation of an anomalous difference density map showing large peaks for the zinc atoms and no peak for the cacodylate arsenic (**fig. S28**). B-factors for the zinc atoms were refined anisotropically to account for the observed disorder (**fig. S28**).

1.17 Specificity study of KABLE1.4 with different benzisoxazoles

1.17.1 pK_a determination

2-Hydroxy-5-iodo-benzonitrile and 2-Hydroxy-5-amino-benzonitrile substrates were obtained from A2B Chem. 100 mM stock solution of 2-hydroxybenzonitrile derivative was prepared in acetonitrile, which was subsequently diluted to 1 mM. 100 mM buffer solutions at various pH ranging from pH 3.0-10.0.0 were prepared: citrate (pH 3.0-5.8); MES (pH 6.0-6.5); HEPES (pH 6.8-7.8); TRIS (pH 8.0-9.0); CHES (pH 9.3-10.0) in presence of 100 mM NaCl. Substituted 2-

bioRxiv preprint doi: https://doi.org/10.1101/2025.01.30.635804; this version posted January 31, 2025. The copyright holder for this preprint (which was not certified by peer review) is the author/funder, who has granted bioRxiv a license to display the preprint in perpetuity. It is made available under aCC-BY 4.0 International license.

hydroxybenzonitrile (1 mM) was further diluted by 10-fold with buffer solutions and absorbance was measured using UV-Vis spectrophotometer (Agilent 8453). Absorbance at the wavelength maxima (λ_{max}) was plotted versus pH using Kaleidagraph (Synergy Software, v 5.0.6) to obtain the pKa of the corresponding 2-hydroxybenzonitrile. All measurements were performed at 22 ºC.

1.17.2 Kinetic characterization of KABLE1.4 for decomposition of different benzisoxazoles

The initial rates for Kemp elimination for all the kinetic assays were corrected for the background rates in buffer without the enzyme. Product concentration for the substituted benzisoxazoles was calculated using experimentally determined extinction coefficient at the respective wavelength maximum as mentioned in **table S7**. Catalytic efficiency (k_{cat}/K_M) as well as individual kinetic parameters (k_{cat} and K_M, where applicable) were determined by fitting the dependence of initial rates on substrate concentration ranging from 100 μM to 1 mM to the Michaelis-Menten equation $v_0 = k_{cat}[E][S]/(K_M+[S])$, where v_0 is the initial velocity, [E] and [S] are enzyme and substrate concentrations, respectively. Enzymes with high K_M values, where substrate saturation could not be achieved due to substrate solubility limits, catalytic efficiency was determined by fitting data to $v_0 = k_{cat}/K_M$ [E][S]. All the substrates were prepared in acetonitrile as 100 mM stock, which was further diluted in buffer (20 mM HEPES, pH 7.0, 100 mM NaCl) to prepare working solutions of various substrate concentrations. The reactions were initiated by mixing enzyme in buffer and substrate solutions in 1:3 ratio in 96-well plate (Genesee Scientific). The final reaction mixtures contained 1 μM enzyme in buffer, 1.5% acetonitrile (to maintain the constant final concentrations of the substrate), and variable concentrations of substrate. Product formation was monitored on a plate reader (BioTek Synergy neo2, Serial: 22052011) at 25 °C for 15 min. with the minimum kinetic interval determined by manufacturer provided software (Gen 5, version 3.12).

bioRxiv preprint doi: https://doi.org/10.1101/2025.01.30.635804; this version posted January 31, 2025. The copyright holder for this preprint (which was not certified by peer review) is the author/funder, who has granted bioRxiv a license to display the preprint in perpetuity. It is made available under aCC-BY 4.0 International license.

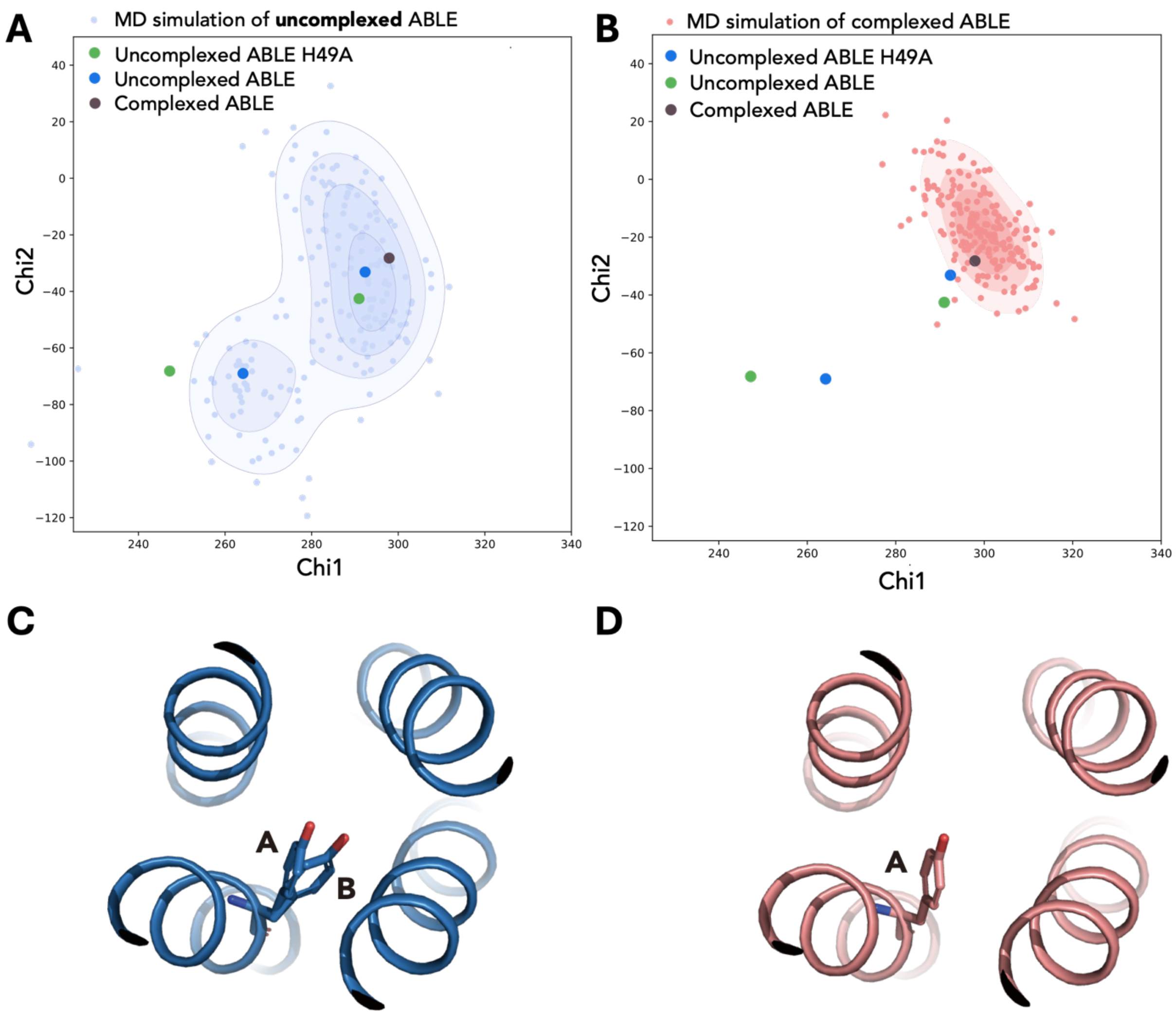

Fig. S1. Contour map of Tyr46 side chain conformation of ABLE from molecular dynamics simulation. (**A&B**) Dynamics of uncomplexed ABLE (PDB: 6W6X) and ABLE-apixaban (PDB: 6W70) were explored by doing molecular simulation at 278 K for 500 ns with amber. Chi1 and Chi2 from each state of these simulations and reported crystal structures of ABLEs (6W6X: uncompleted ABLE; 6W70: ABLE-Apixaban Complex; 6X8N: uncomplexed ABLE His49Ala) were extracted for plotting. Contours were generated from the points of uncomplexed ABLE (**A**) and ABLE-apixaban complex contours were generated using an in-house script that utilizes the Gaussian kernel density estimate methods from scipy.stats module of SciPy Python Packages. (**C&D**) **A** and **B** conformations of Tyr46 sidechain were present in the uncomplexed ABLE crystal structure (PDB: 6W6X) while only A conformation of Tyr46 sidechain conformations was present at ABLE-apixaban complex(PDB: 6W70).

bioRxiv preprint doi: https://doi.org/10.1101/2025.01.30.635804; this version posted January 31, 2025. The copyright holder for this preprint (which was not certified by peer review) is the author/funder, who has granted bioRxiv a license to display the preprint in perpetuity. It is made available under aCC-BY 4.0 International license.

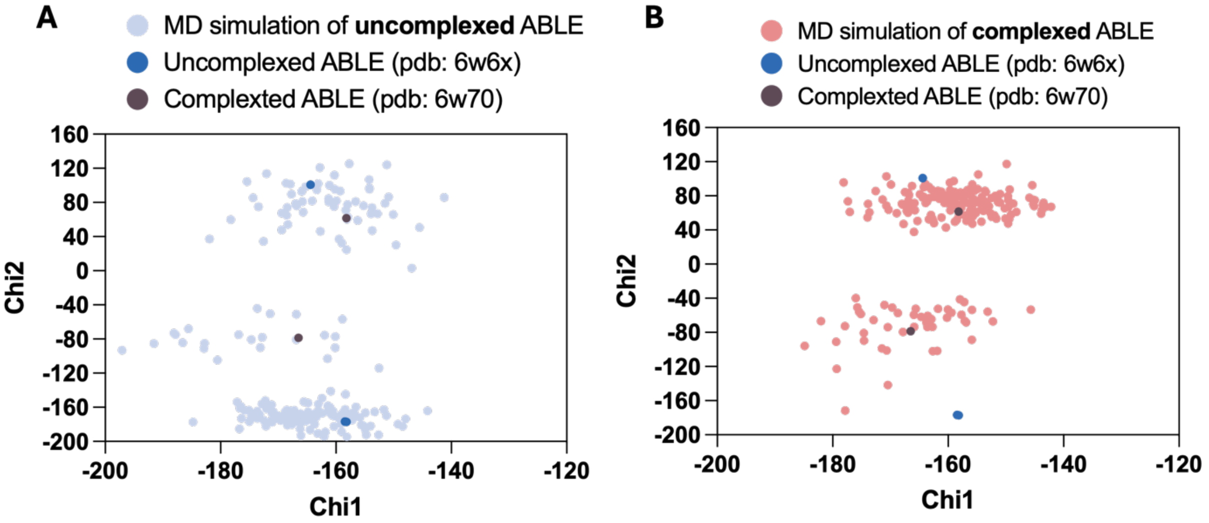

Fig. S2. Contour map of His49 side chain conformation of ABLE from molecular dynamics simulation. (**A-B**) Dynamics of uncomplexed ABLE (PDB: 6W6X, **A**) and complexed ABLE (PDB: 6W70, **B**) were explored by doing molecular simulation at 278 K for 500 ns with amber. Chi1 and Chi2 of His49 from each state of these simulations and reported crystal structures of ABLE were extracted for plotting.

bioRxiv preprint doi: https://doi.org/10.1101/2025.01.30.635804; this version posted January 31, 2025. The copyright holder for this preprint (which was not certified by peer review) is the author/funder, who has granted bioRxiv a license to display the preprint in perpetuity. It is made available under aCC-BY 4.0 International license.

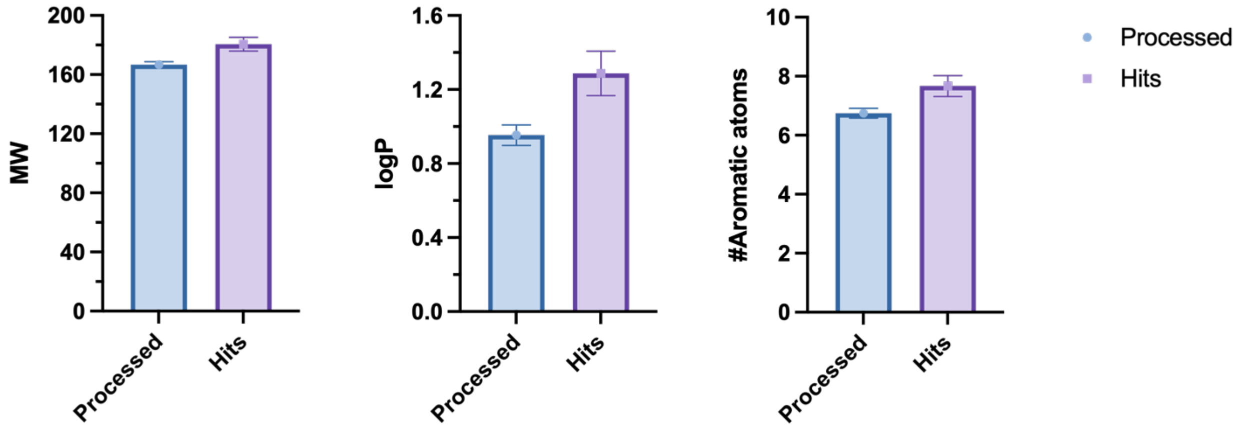

Figure S3: Chemical properties of fragments in the processed diffraction dataset and hits binding at apixaban-binding pocket of ABLE. For each chemical, its MW, logP and the number of aromatic atoms were generated using “calculate properties” function of DataWarrior. The error bars represent standard error of mean. Processed: n=242; Hits: n=39. Z scores are all over 3.

bioRxiv preprint doi: https://doi.org/10.1101/2025.01.30.635804; this version posted January 31, 2025. The copyright holder for this preprint (which was not certified by peer review) is the author/funder, who has granted bioRxiv a license to display the preprint in perpetuity. It is made available under aCC-BY 4.0 International license.

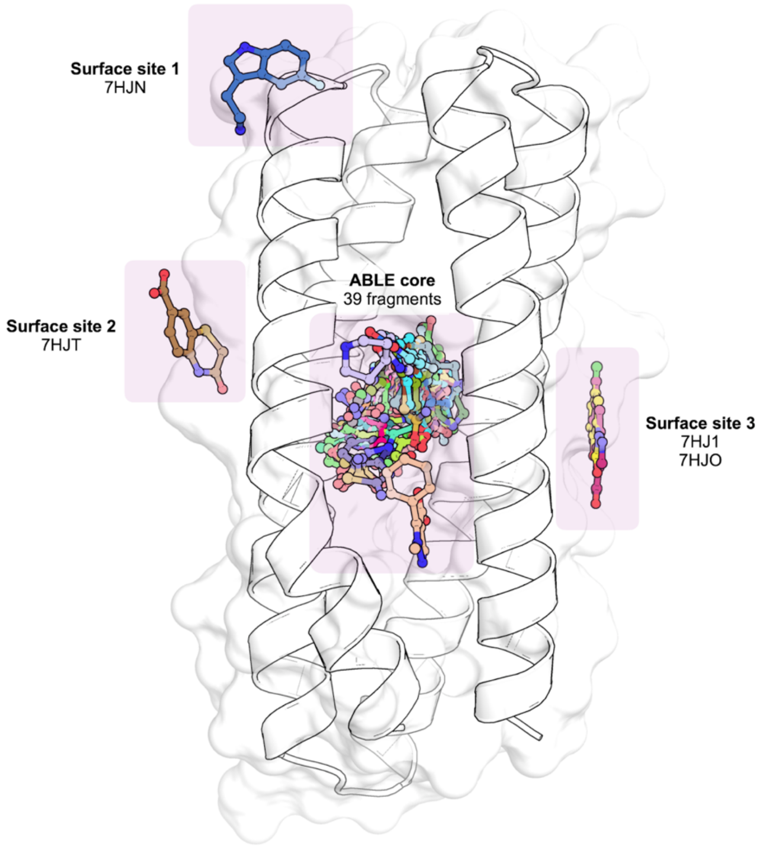

Figure S4: X-ray crystal structure of ABLE showing binding sites for 43 fragments. The structure of apo ABLE (PDB: 9DW2) with white cartoon and transparent white surface.

bioRxiv preprint doi: https://doi.org/10.1101/2025.01.30.635804; this version posted January 31, 2025. The copyright holder for this preprint (which was not certified by peer review) is the author/funder, who has granted bioRxiv a license to display the preprint in perpetuity. It is made available under aCC-BY 4.0 International license.

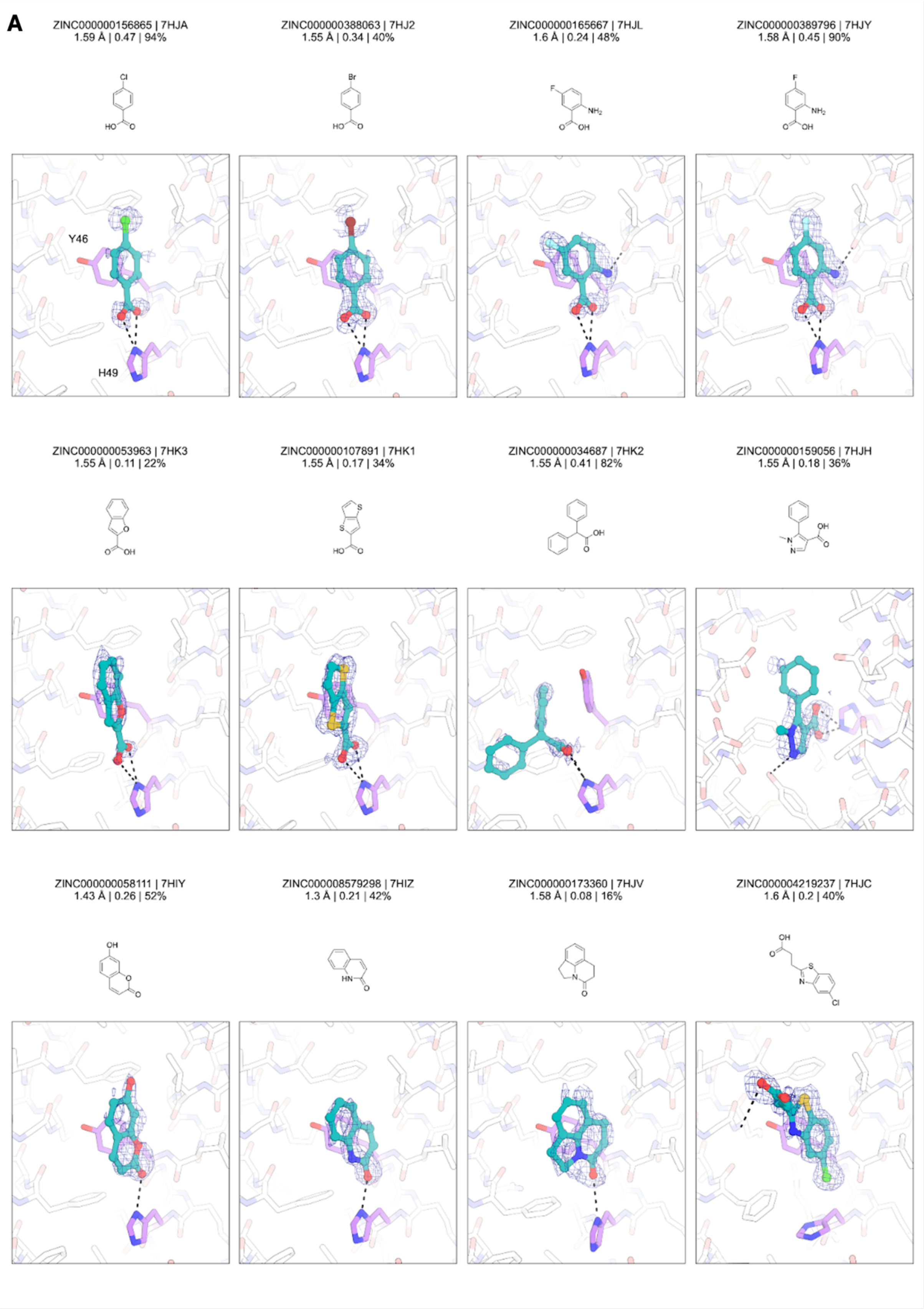

bioRxiv preprint doi: https://doi.org/10.1101/2025.01.30.635804; this version posted January 31, 2025. The copyright holder for this preprint (which was not certified by peer review) is the author/funder, who has granted bioRxiv a license to display the preprint in perpetuity. It is made available under aCC-BY 4.0 International license.

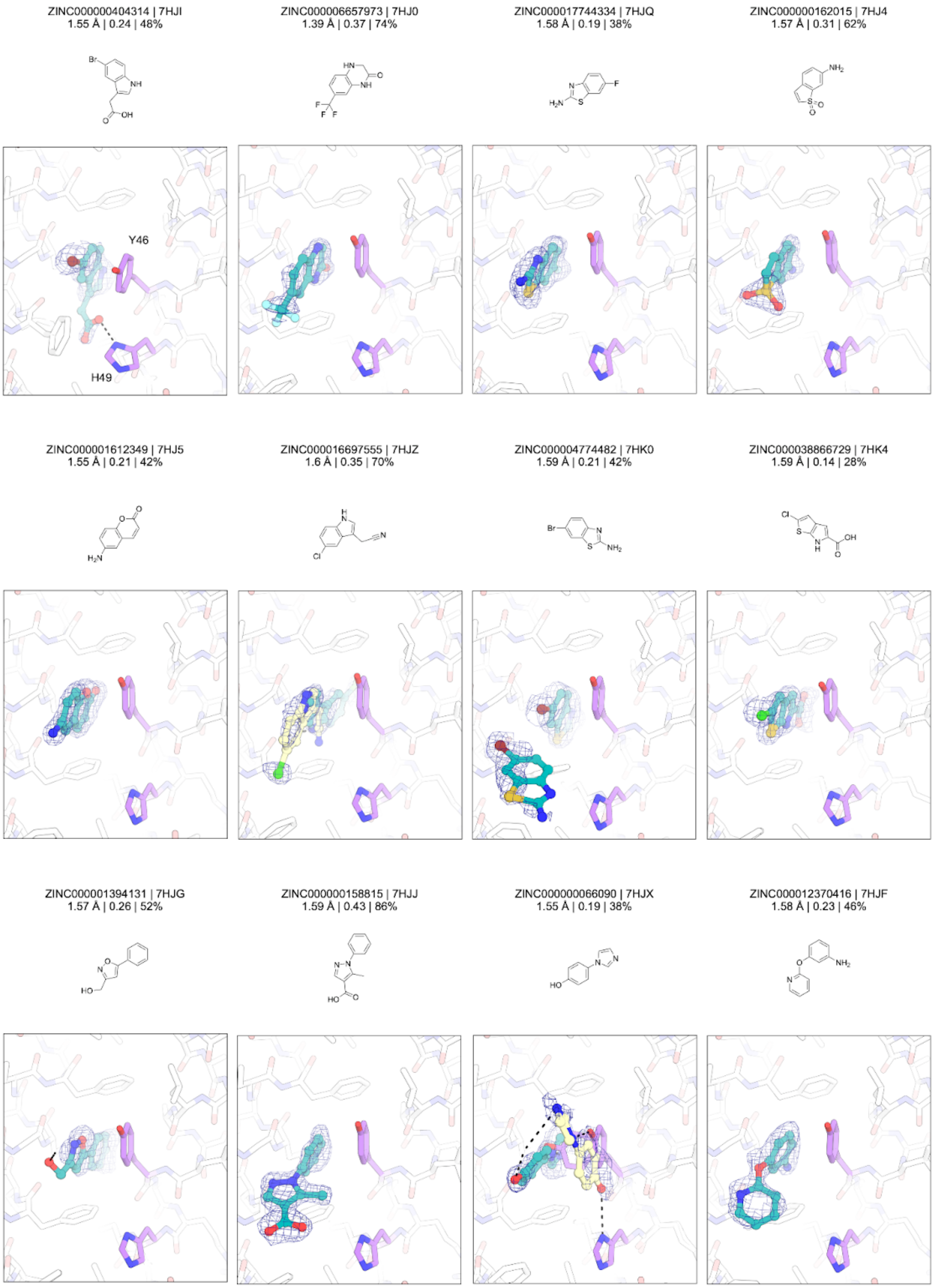

bioRxiv preprint doi: https://doi.org/10.1101/2025.01.30.635804; this version posted January 31, 2025. The copyright holder for this preprint (which was not certified by peer review) is the author/funder, who has granted bioRxiv a license to display the preprint in perpetuity. It is made available under aCC-BY 4.0 International license.

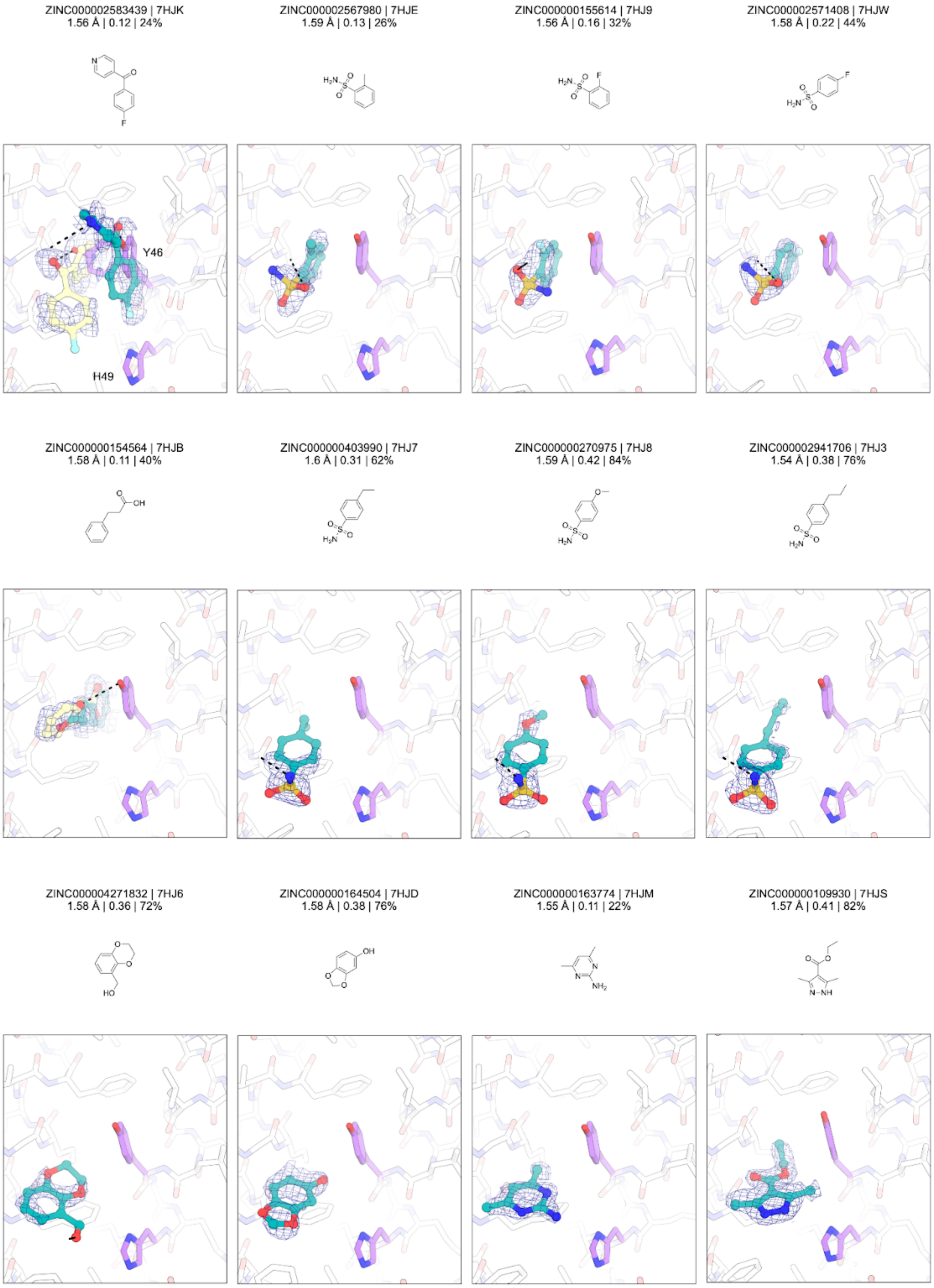

bioRxiv preprint doi: https://doi.org/10.1101/2025.01.30.635804; this version posted January 31, 2025. The copyright holder for this preprint (which was not certified by peer review) is the author/funder, who has granted bioRxiv a license to display the preprint in perpetuity. It is made available under aCC-BY 4.0 International license.

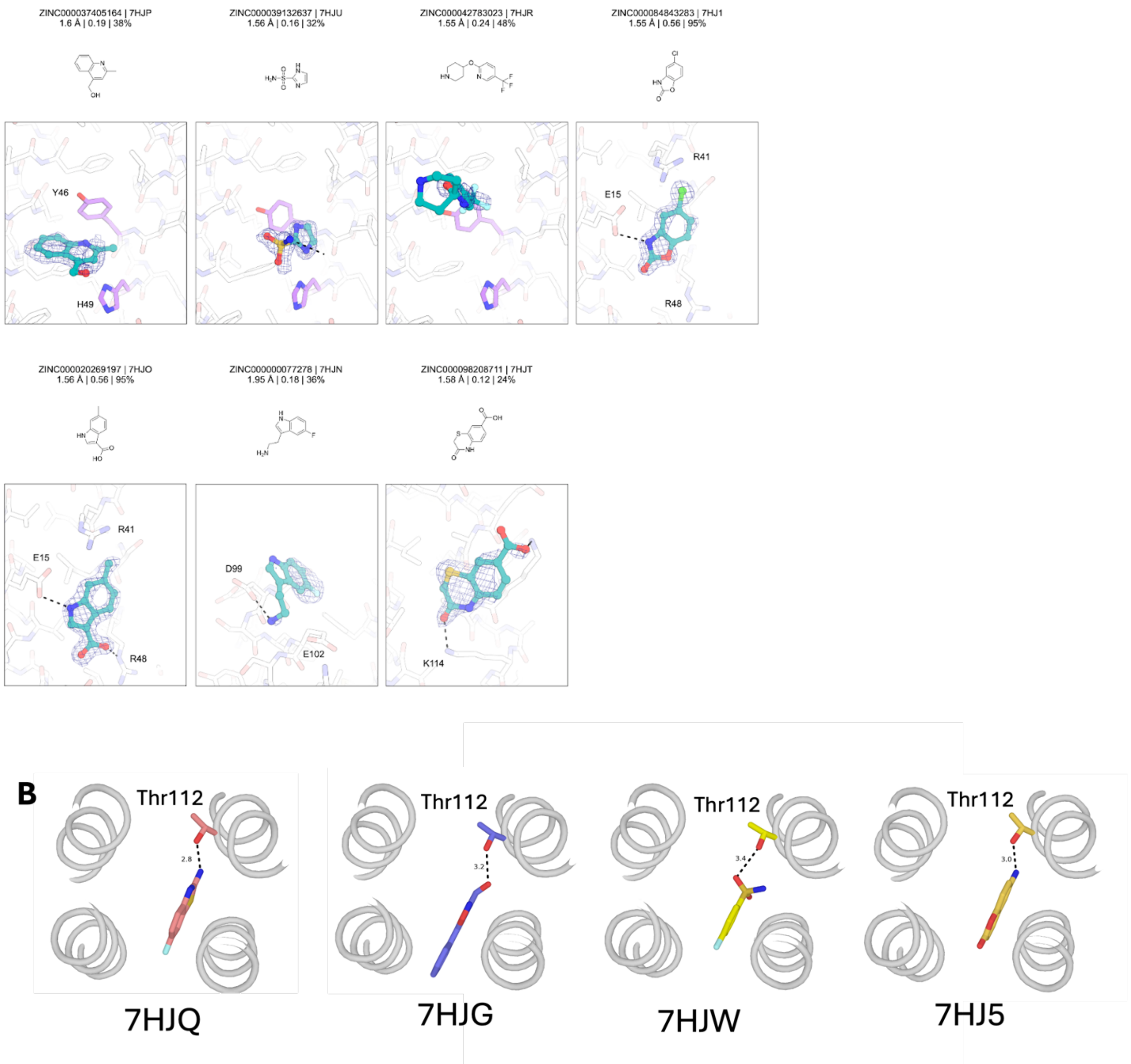

Figure S5: Chemical structures and electron density maps for the 43 fragment structures reported in this work. (**A**) PanDDA event maps (blue mesh, 2 σ) are contoured around fragments (teal/yellow sticks). The sidechains of residues Tyr46 and His49 are shown with purple sticks. Fragment names, PDB codes, resolution, 1-BDC value and refined occupancies are indicated. Hydrogen bonds are shown with dashed black lines. For clarity, residues 10-23 and 105-118 are hidden for fragments binding in the ABLE core. His49-mediated polar interaction could be found in majority of fragments at site B. (**B**) Another view to show the Thr112-mediated polar interactions in many fragments at site A, whose PDB codes are labelled at the bottom.

bioRxiv preprint doi: https://doi.org/10.1101/2025.01.30.635804; this version posted January 31, 2025. The copyright holder for this preprint (which was not certified by peer review) is the author/funder, who has granted bioRxiv a license to display the preprint in perpetuity. It is made available under aCC-BY 4.0 International license.

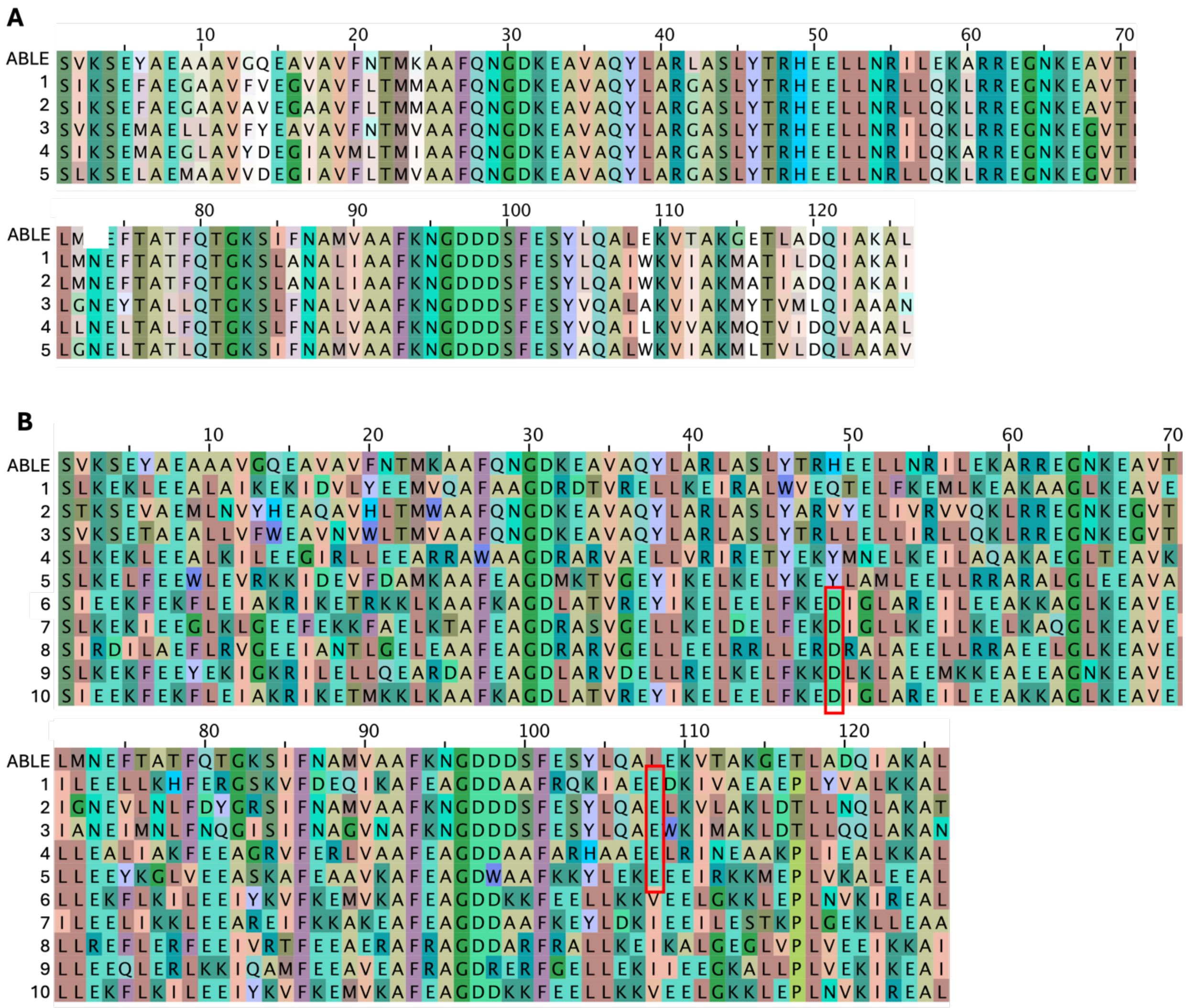

Figure S6. Sequence comparison between ABLE and FABLEs/KABLEs. (A) Amino acid sequence of all 5 FABLE designs (FABLE$_{D1}$ to FABLE$_{D5}$), with ABLE shown on the top. (**B**) Amino acid sequence of all 10 KABLE designs (KABLE$_{D1}$ to KABLE$_{D10}$), with ABLE shown on the top. The designed base Glu108 in the first strategy (KABLE$_{D1}$ to KABLE$_{D5}$) and Asp49 in the second strategy (KABLE$_{D6}$ to KABLE$_{D10}$) are highlighted in the red box.

bioRxiv preprint doi: https://doi.org/10.1101/2025.01.30.635804; this version posted January 31, 2025. The copyright holder for this preprint (which was not certified by peer review) is the author/funder, who has granted bioRxiv a license to display the preprint in perpetuity. It is made available under aCC-BY 4.0 International license.

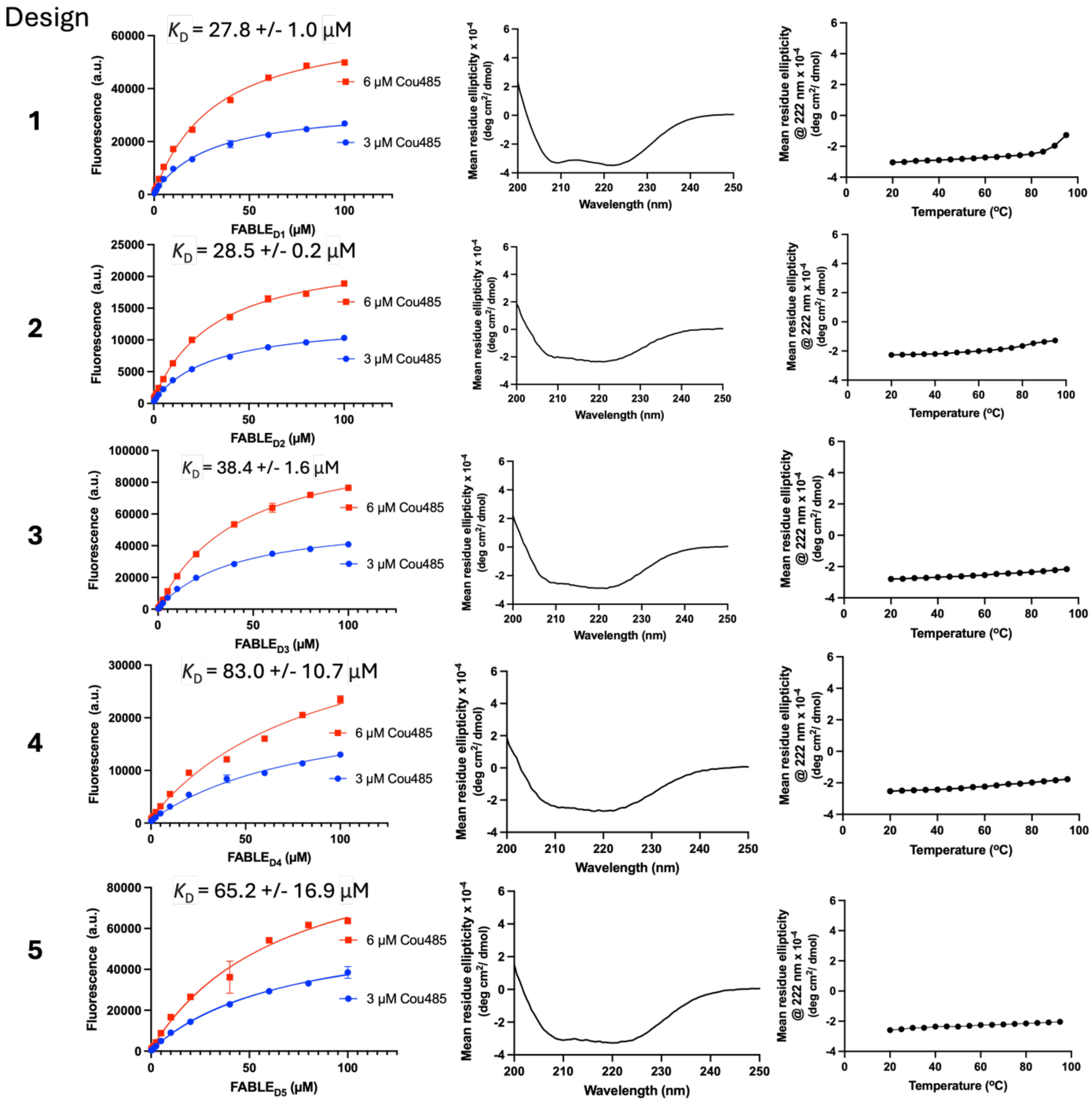

Figure S7. Characterization of five FABLE Designs. Left column: Fluorescence of Cou485 at 2 fixed concentrations of 3 μM and 6 μM were measured in the presence of increasing amounts of each protein. The binding constants were obtained by fitting with the equation in the section 1.7 of SI. The error bars represent standard deviations. Middle Column: circular dichroism spectra shows that all the designs are helical proteins. Right column: temperature-dependent circular dichroism signals measured at 222 nm show that all designs are thermostable. The design 1 was designated as FABLE.

bioRxiv preprint doi: https://doi.org/10.1101/2025.01.30.635804; this version posted January 31, 2025. The copyright holder for this preprint (which was not certified by peer review) is the author/funder, who has granted bioRxiv a license to display the preprint in perpetuity. It is made available under aCC-BY 4.0 International license.

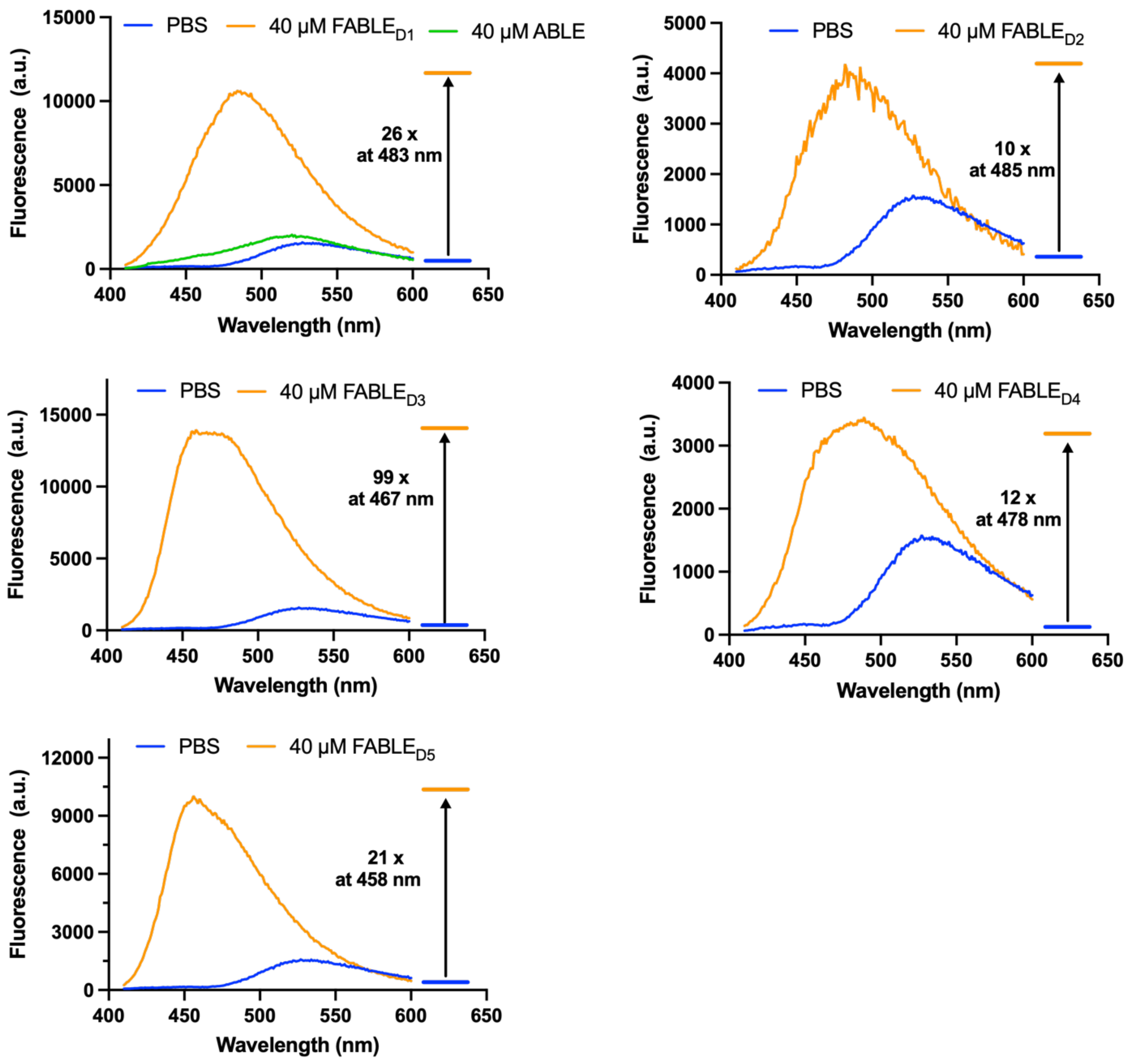

Figure S8. Emission Spectra of Cou485 in the presence of FABLE protein derivatives. The fluorescence emission spectrum of 6 μM Cou485 was recorded using BioTek Synergy Neo2 Reader with an excitation of 395 nm. The indicated FABLEs or ABLE (control) were present at 40 μM.

bioRxiv preprint doi: https://doi.org/10.1101/2025.01.30.635804; this version posted January 31, 2025. The copyright holder for this preprint (which was not certified by peer review) is the author/funder, who has granted bioRxiv a license to display the preprint in perpetuity. It is made available under aCC-BY 4.0 International license.

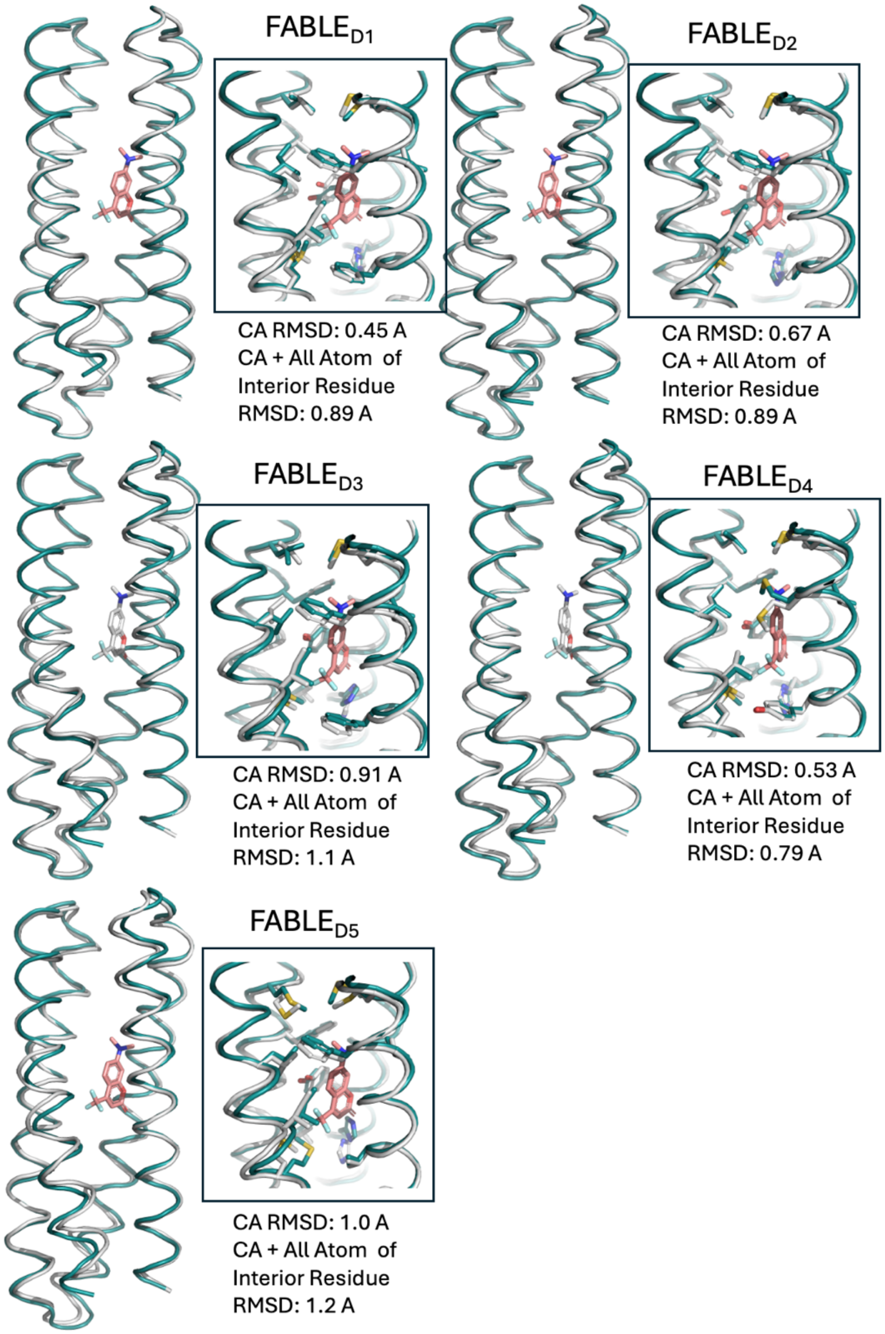

Fig. 9: AF3 prediction agrees with all 5 FABLE designs. Left: Designed structure (protein in teal cartoon and ligand in pink stick) and predicted structure (protein in grey cartoon). Right: Zoom in view of active site with carbon in designed structures shown as the teal stick and carbon in predicted structures shown as a grey stick.

bioRxiv preprint doi: https://doi.org/10.1101/2025.01.30.635804; this version posted January 31, 2025. The copyright holder for this preprint (which was not certified by peer review) is the author/funder, who has granted bioRxiv a license to display the preprint in perpetuity. It is made available under aCC-BY 4.0 International license.

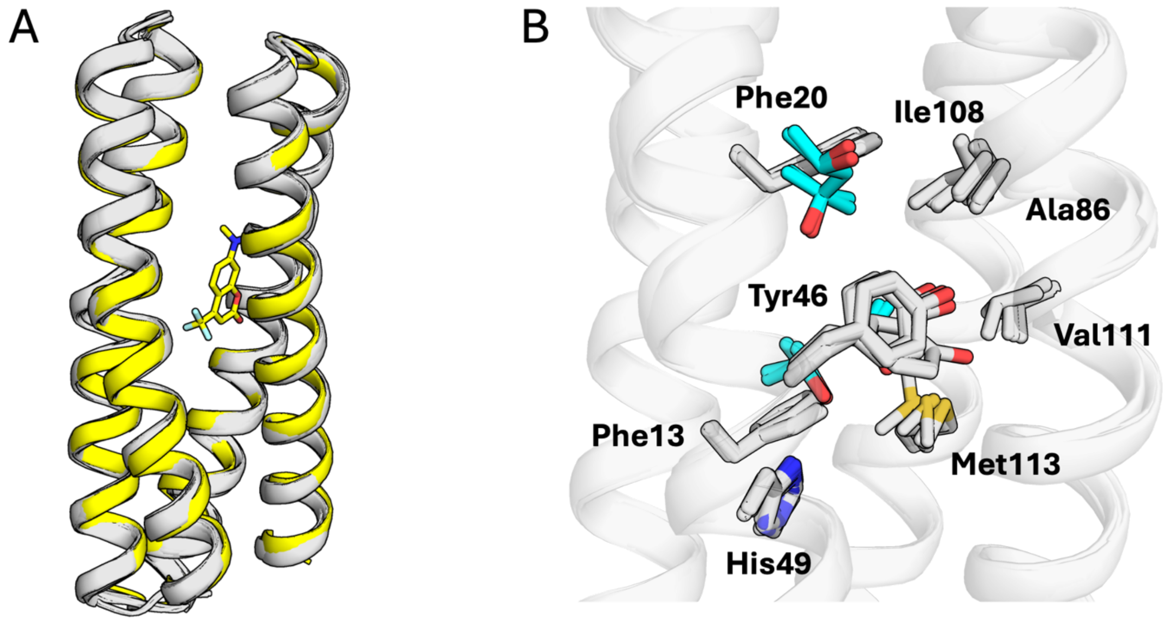

Fig. 10: The crystal structure of apo FABLE agrees with the designed structure. (A) Overlay of designed structure (yellow carbon) with structures from the individual asymmetric unit in the three crystal structures (9DWA, 9DWB, 9DWC) of FABLE (grey carbon). The Cou485 in the designed structure are shown in yellow sticks. The low solubility of Cou485 under crystallization conditions limited our ability to obtain a co-crystal structure. However, the un-complexed structures are in excellent agreement with the model of the complex (with sub-Å of Cα RMSD, table S1). (B) Active site view of structures from the individual asymmetric units in three crystal structures (9DWA, 9DWB, 9DWC) of FABLE. The sidechains of active site residues are shown in sticks. Two methylpentanediol molecules from the crystallization buffer, which occupy the binding site in 9DWA, are shown in blue carbons. Most residues are very well organized, including Met23. Tyr46 shows some variability in the different crystal environments

bioRxiv preprint doi: https://doi.org/10.1101/2025.01.30.635804; this version posted January 31, 2025. The copyright holder for this preprint (which was not certified by peer review) is the author/funder, who has granted bioRxiv a license to display the preprint in perpetuity. It is made available under aCC-BY 4.0 International license.

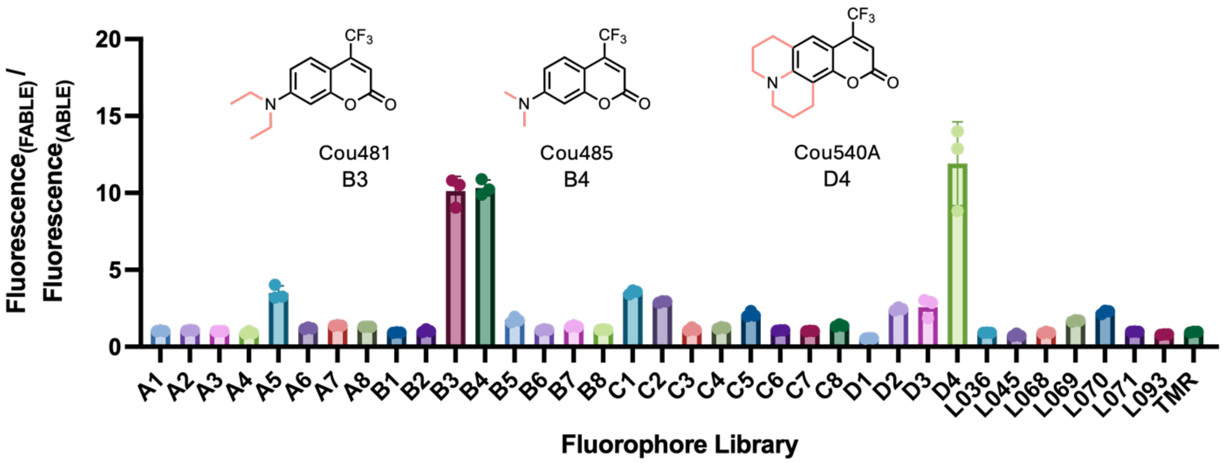

Figure S11: Exploring the chemical space of FABLE. The excitation and emission of fluorophores are in **Table S3**. The error bars represent standard deviations. (see section 1.5 of SI for experimental details)

bioRxiv preprint doi: https://doi.org/10.1101/2025.01.30.635804; this version posted January 31, 2025. The copyright holder for this preprint (which was not certified by peer review) is the author/funder, who has granted bioRxiv a license to display the preprint in perpetuity. It is made available under aCC-BY 4.0 International license.

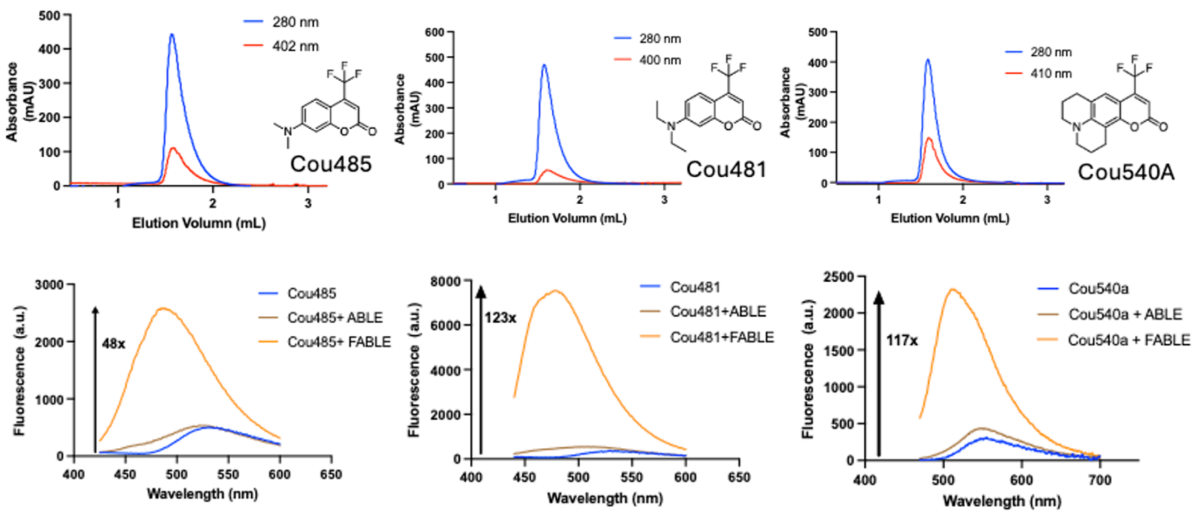

Fig. S12: FABLE interacts with Cou485, Cou481 and Cou540a as evidenced by SEC and an increase in fluorescence intensity by 48-123 fold. SEC analysis of 100 µM FABLE mixed with 200 µM indicated coumarin revealed a monomeric peak for FABLE, with the protein coeluting with the coumarin at the same time. Incubation of 1.5 µM Cou485 and Cou481 with 40 µM FABLE increased fluorescence intensity at 470 nm by 48 and 122 fold, respectively. Similarly, incubation of 0.5 µM Cou540a with 6 µM FABLE resulted in a 117-fold increase in fluorescence at 510 nm.

bioRxiv preprint doi: https://doi.org/10.1101/2025.01.30.635804; this version posted January 31, 2025. The copyright holder for this preprint (which was not certified by peer review) is the author/funder, who has granted bioRxiv a license to display the preprint in perpetuity. It is made available under aCC-BY 4.0 International license.

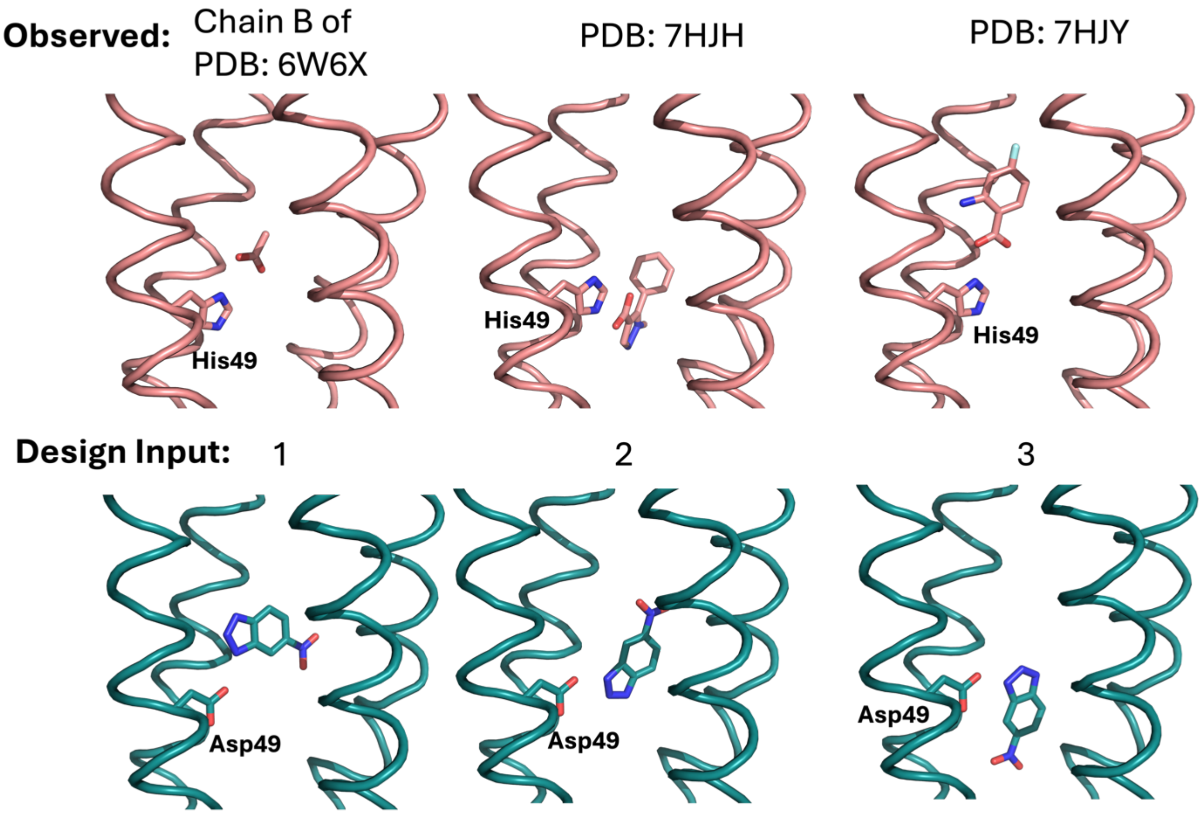

Figure S13. Observed His49-carboxylic interactions in ABLE-fragment complexes and the Asp49-6NBI interactions used as starting points to design KABLE1. Top: Examples of observed ABLE-fragment interactions with indicated PDB code, carbon of His49 and fragments are shown in pink sticks. Bottom: three initial input structure for designing KABLE1 with each utilizing one distinctive lone pair of electron on either OD1 or OD2 of Asp49 (see see section 1.11.2 of SI). Carbon of Asp49 and fragments are shown in teal sticks. Similar observed orientations of residue 49 and fragment are used as input to design KABLE1.

bioRxiv preprint doi: https://doi.org/10.1101/2025.01.30.635804; this version posted January 31, 2025. The copyright holder for this preprint (which was not certified by peer review) is the author/funder, who has granted bioRxiv a license to display the preprint in perpetuity. It is made available under aCC-BY 4.0 International license.

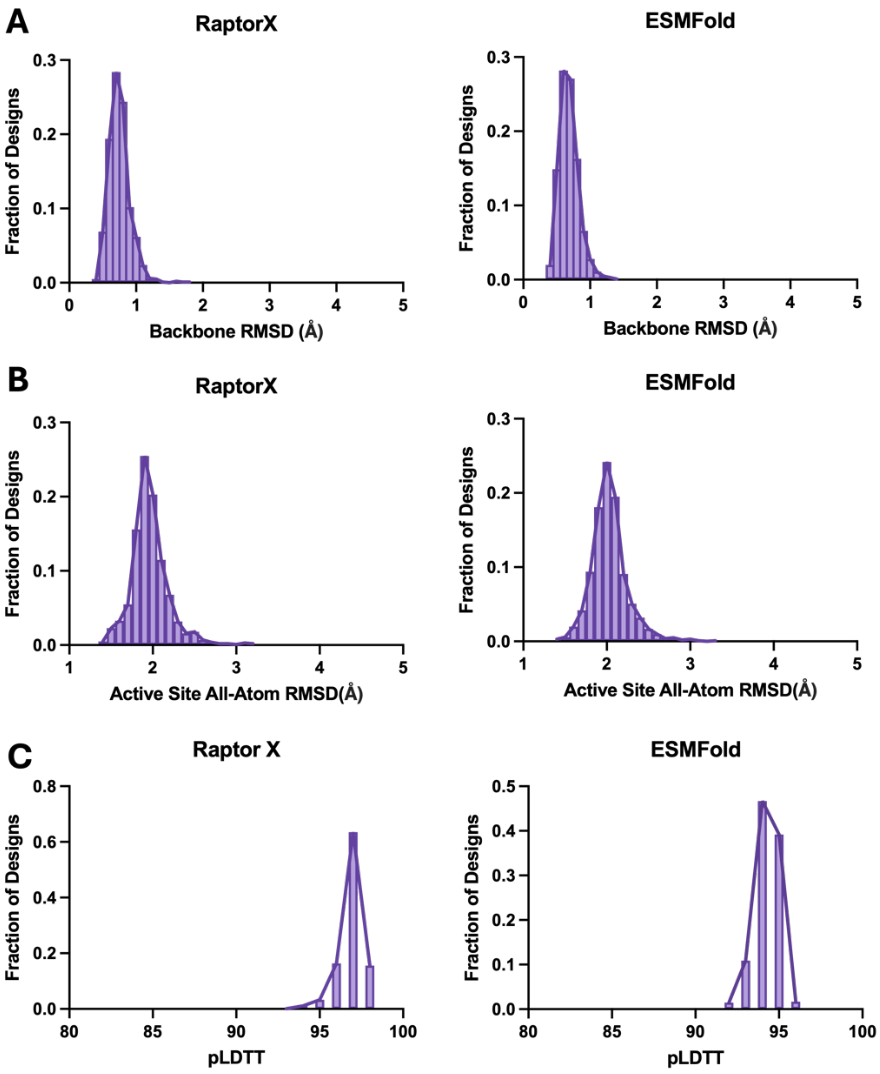

Figure S14: Structure prediction metrics of KABLE designs bearing Asp49. All 1000 sequences were subjected to structure prediction with both RaptorX and ESMFold. (**A**) Backbone RMSD between either prediction and design shows most of designs are within 1 Å backbone RMSD. (**B**) All-atom RMSD of active site residues (< 3.5 Å of TSA) between either prediction and design shows the median of active site RMSD at 2 Å. (**C**) pLDTT scores from both prediction methods show all the designs are with pLDTT score over 90.

bioRxiv preprint doi: https://doi.org/10.1101/2025.01.30.635804; this version posted January 31, 2025. The copyright holder for this preprint (which was not certified by peer review) is the author/funder, who has granted bioRxiv a license to display the preprint in perpetuity. It is made available under aCC-BY 4.0 International license.

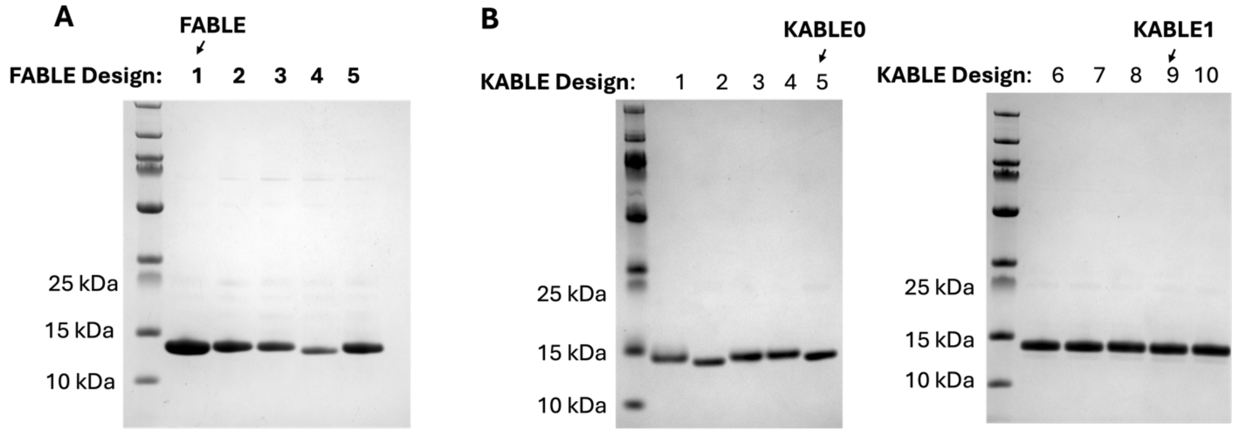

Figure S15. SDS-PAGE Characterization of all designed proteins. **(A)** SDS-PAGE analysis of the purified form of all 5 FABLE designs ($FABLE_{D1}$-$FABLE_{D5}$), with the $FABLE_{D1}$ designated as FABLE. **(B)** SDS-PAGE analysis of all 10 KABLE designs from the first design strategy of aromatic box ($KABLE_{D1}$-$KABLE_{D5}$) and the second design strategy of versatile residue 49 ($KABLE_{D6}$-$KABLE_{D10}$), with the $KABLE_{D5}$ highlighted as KABLE0 and $KABLE_{D9}$ highlighted as KABLE1.

bioRxiv preprint doi: https://doi.org/10.1101/2025.01.30.635804; this version posted January 31, 2025. The copyright holder for this preprint (which was not certified by peer review) is the author/funder, who has granted bioRxiv a license to display the preprint in perpetuity. It is made available under aCC-BY 4.0 International license.

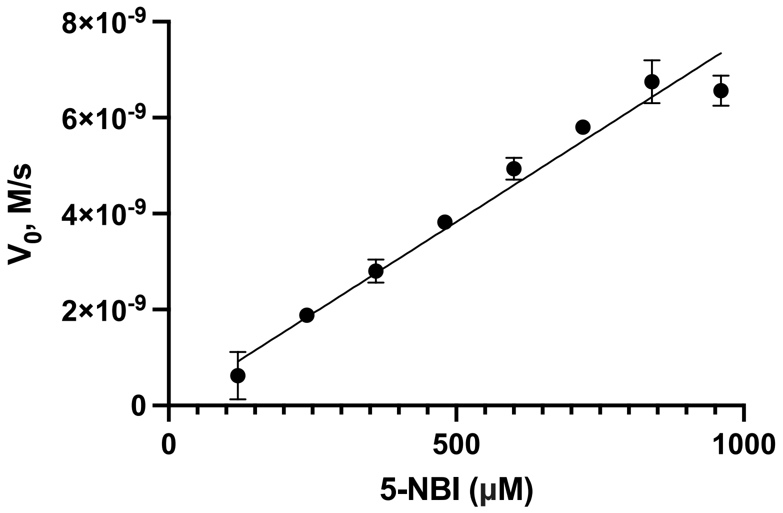

Figure S16: Substrate concentration dependence versus rate data for KABLE0. The reaction was measured with 1 μM KABLE0 in pH 8 buffer. Background reaction at the same condition without enzyme was subtracted and data were analyzed by least squares analysis to the linear portion of the Michaelis-Menten model ($v_0 = (k_{cat}/K_M)[E_0][S]$), and k_{cat}/K_M was deduced from the slope. Error bar stands for standard deviations.

bioRxiv preprint doi: https://doi.org/10.1101/2025.01.30.635804; this version posted January 31, 2025. The copyright holder for this preprint (which was not certified by peer review) is the author/funder, who has granted bioRxiv a license to display the preprint in perpetuity. It is made available under aCC-BY 4.0 International license.

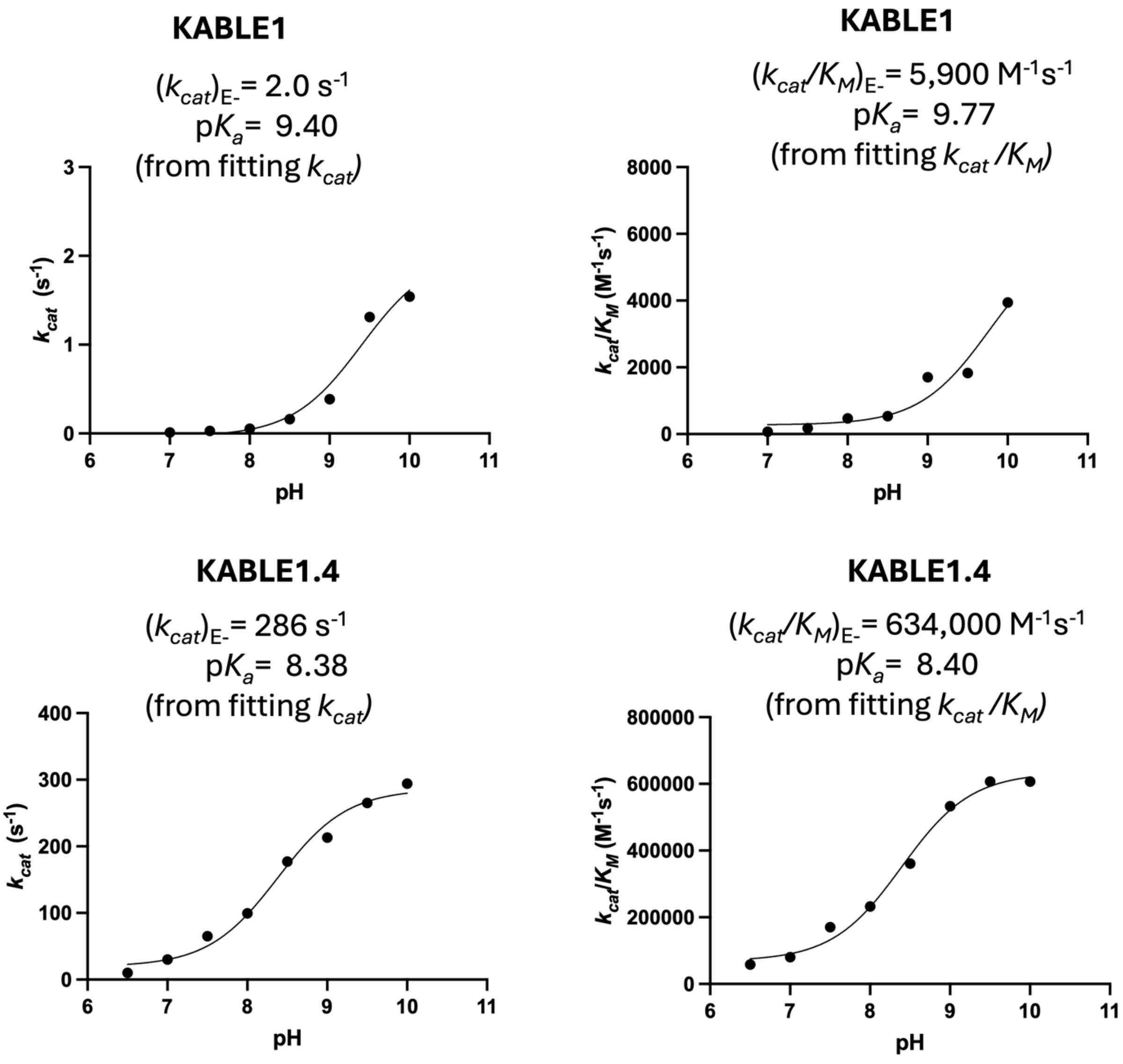

Figure S17: Dependence of k_{cat}/K_M and k_{cat} on pH. For each pH, its initials rate under a series of substrate concentration was used to fit the k_{cat}/K_M and k_{cat} for each pH using Michaelis-Menton equation at GraphPad Prism. The dependence of k_{cat}/K_M and k_{cat} on pH was fitted using the equation $k_{cat}/K_M = (k_{cat}/K_M)_{EH} \times (1-f_{E-}) + (k_{cat}/K_M)_{E-} \times f_{E-}$ or $k_{cat} = (k_{cat})_{EH} \times (1-f_{E-}) + (k_{cat})_{E-} \times f_{E-}$, where f_{E-} is the fraction of enzyme in deprotonated form. See **section 1.7** of SI for details

bioRxiv preprint doi: https://doi.org/10.1101/2025.01.30.635804; this version posted January 31, 2025. The copyright holder for this preprint (which was not certified by peer review) is the author/funder, who has granted bioRxiv a license to display the preprint in perpetuity. It is made available under aCC-BY 4.0 International license.

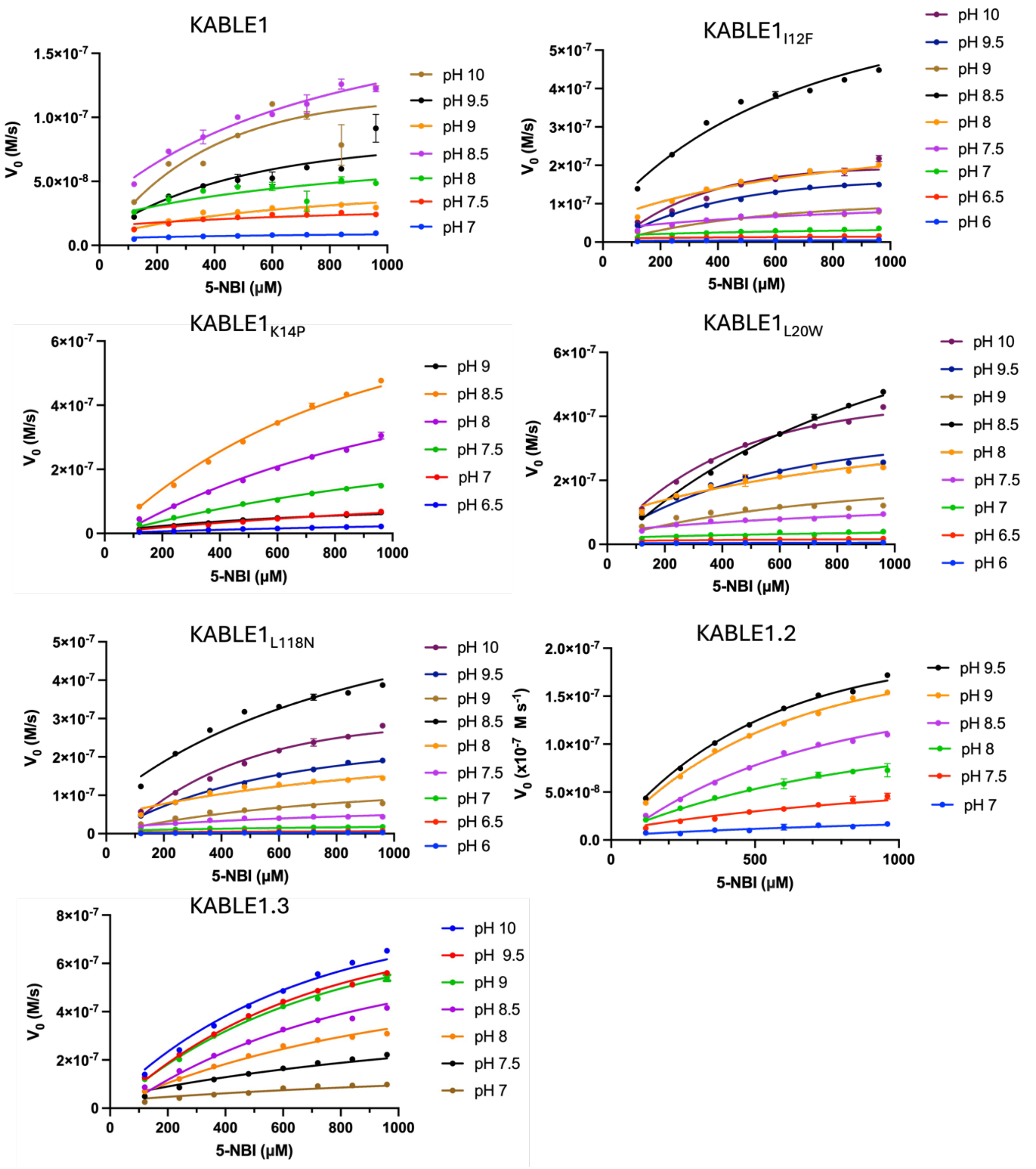

Figure S18: Global fitting of k_{cat}, K_M and pK_a for the Kemp eliminases.. The initial velocities at all substrate concentrations and pH were used to fit a shared k_{cat}, K_M and pK_a at each pH. For KABLE1$_{K14P}$, KABLE1.2, KABLE1.3, KABLE1.4, The value of k_{cat}/K_M was well described, but the fit values of k_{cat} and K_M tended to covary. The optimal shared value of k_{cat} was therefore obtained from an R^2- analysis, which is shown in Fig. S15. The optimal k_{cat} was used to fit the shared K_M and pK_a. Its standard deviation was obtained by taking the highest ratio of standard deviation to mean of the fitted k_{cat}, considering that there may be more error for the values of the of k_{cat} from R^2 analysis. Protein concentrations used for different pH are summarized in Table. S5. The error bar represents the standard error of the mean. Kinetic parameters and pK_a are summarized in table S4.

bioRxiv preprint doi: https://doi.org/10.1101/2025.01.30.635804; this version posted January 31, 2025. The copyright holder for this preprint (which was not certified by peer review) is the author/funder, who has granted bioRxiv a license to display the preprint in perpetuity. It is made available under aCC-BY 4.0 International license.

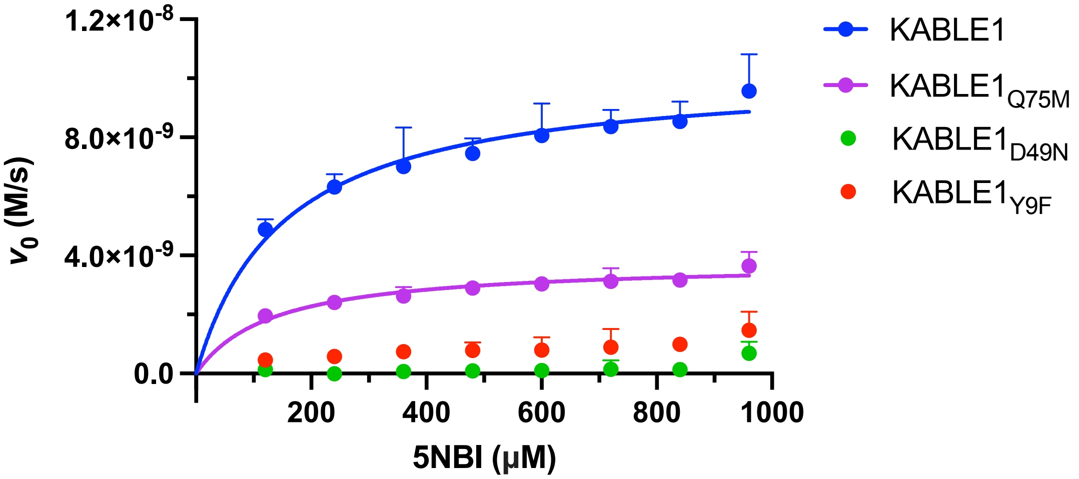

Figure S19. Substrate concentration versus rate data for KABLE1 mutants at Q75 and Y9 and D49. Reactions were measured at pH 7.0 buffer (20 mM HEPES,100 mM NaCl) with 1 μM KABLE1, 2.5 μM KABLE1$_{Q75M}$, 2.5 μM KABLE1$_{Y9F}$ and 2.5 μM KABLE1$_{D49N}$, respectively. Fitting the data with Michaelis-Menten model yielded KABLE1 (k_{cat}/K_M = 68 ± 28 M^{-1} s^{-1}, k_{cat} = 1.0 ± 0.10 x10^{-2} s^{-1}, K_M = 0.15 ± 0.023 mM^{-1}) and KABLE1$_{Q75M}$ (k_{cat}/K_M = 11 +7.3 M^{-1} s^{-1}, k_{cat} = 0.15 ± 0.013 x10^{-2} s^{-1}, K_M = 0.13 ± 0.083 mM^{-1}). For KABLE1$_{Q75M}$ and KABLE1$_{D49N}$, the fitting was not able to return reliable parameters. Error bar represents standard deviation.

bioRxiv preprint doi: https://doi.org/10.1101/2025.01.30.635804; this version posted January 31, 2025. The copyright holder for this preprint (which was not certified by peer review) is the author/funder, who has granted bioRxiv a license to display the preprint in perpetuity. It is made available under aCC-BY 4.0 International license.

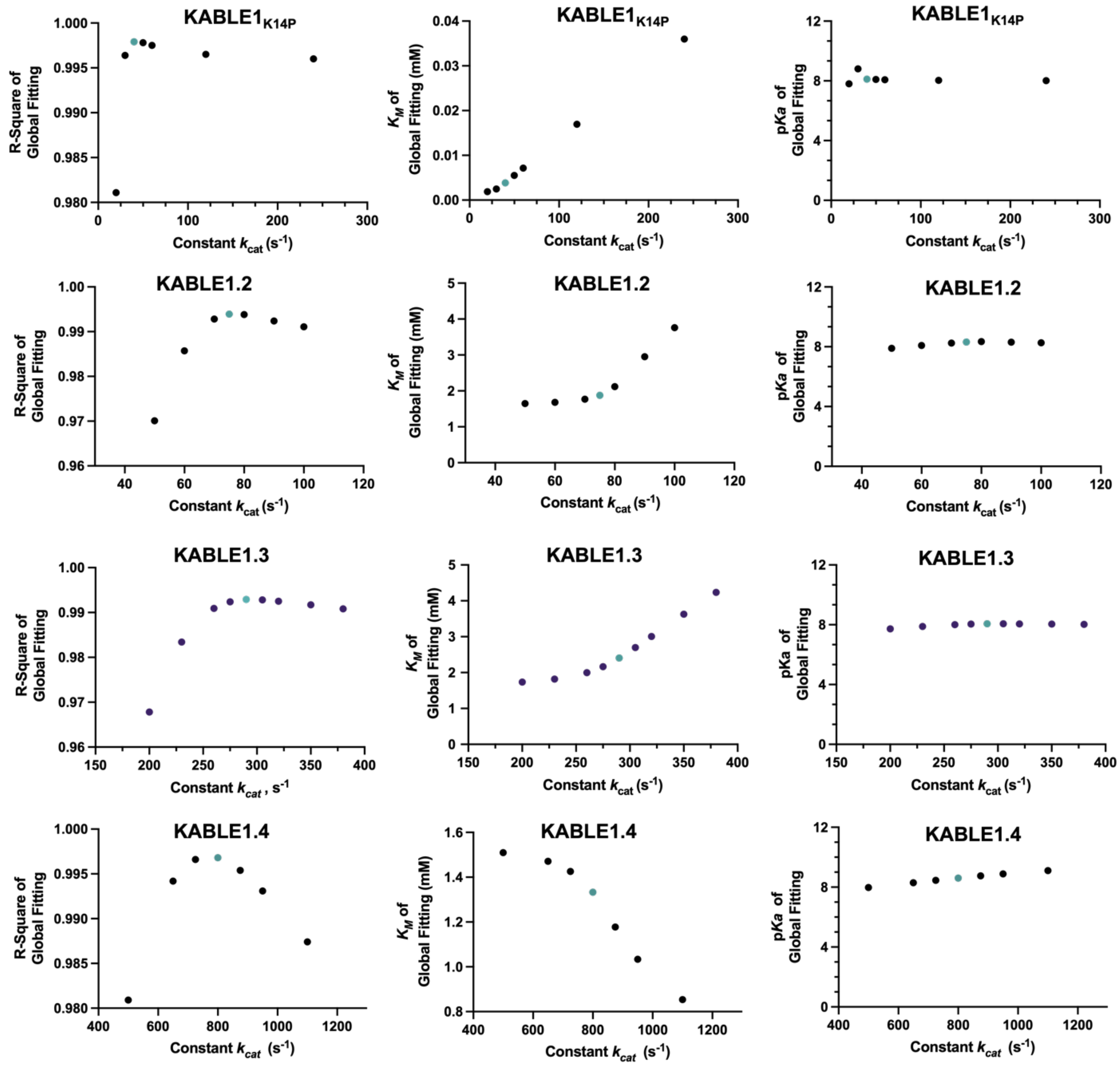

Figure S20. R-square analysis of KABLE1$_{K14P}$, KABLE1.2, KABLE1.3, KABLE1.4. A series of constant k_{cat} was used to globally fit the K_M and pK_a. The constant k_{cat} (in green) with the highest R-square was selected as the optimal k_{cat}. K_M covaries to the constant k_{cat} and minimal effect of constant k_{cat} on pK_a indicates the pK_a is stable over a wide range of constant pK_a around the optimal pK_a.

bioRxiv preprint doi: https://doi.org/10.1101/2025.01.30.635804; this version posted January 31, 2025. The copyright holder for this preprint (which was not certified by peer review) is the author/funder, who has granted bioRxiv a license to display the preprint in perpetuity. It is made available under aCC-BY 4.0 International license.

A

```
ABLE               SVKSEYAEAAAVGQEAVAVFNTMKAAFQNGDKEAVAQYLARLASLYTRHEELLNRILEKA 60
KABLE1.2           SLKEKFEEYEKIGPRILELWQEARDAFEAGDLARVDELLRELKELFKKDLKLAEEMKKEA 60
KABLE1.2.crystal   SLKEKFAEYEAIGPRILELWQAARNAFEAGDLARVANLLAELKELFKKDLNLANAMAAEA 60

ABLE               RREGNKEAVTLMNEFTATFQTGKSIFNAMVAAFKNGDDDSFESYLQALEKVTAKGETLAD 120
KABLE1.2           EEAGNKEAVELLEEQLERLKKIQAMFEEAVEAFRAGDRERFGELLEKIIEEGKALLPLVE 120
KABLE1.2.crystal   AEAGNKEAVALLAEQLERLKKIQAMFAAAVNAFRAGDREAFGALLEAIINEGKALLPLVE 120

ABLE               QIAKAL 126
KABLE1.2           KIKEAI 126
KABLE1.2.crystal   AIKEAI 126
```

B

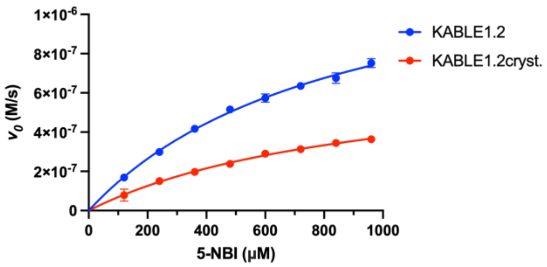

Figure S21. Substrate concentration versus rate data for KABLE1.2 and KABLE1.2$_{cryst}$. (A) Amino acid sequence alignment of ABLE with KABLE1.2 and KABLE1.2$_{cryst}$, shows the surface residues of ABLE were grafted to KABLE1.2$_{cryst}$. **(B)** Reactions were measured at pH 8.0 buffer (20 mM Tris,100 mM NaCl) with 0.1 μM KABLE1.2 and KABLE1.2$_{cryst,}$ respectively. Error bars represent standard deviations.

bioRxiv preprint doi: https://doi.org/10.1101/2025.01.30.635804; this version posted January 31, 2025. The copyright holder for this preprint (which was not certified by peer review) is the author/funder, who has granted bioRxiv a license to display the preprint in perpetuity. It is made available under aCC-BY 4.0 International license.

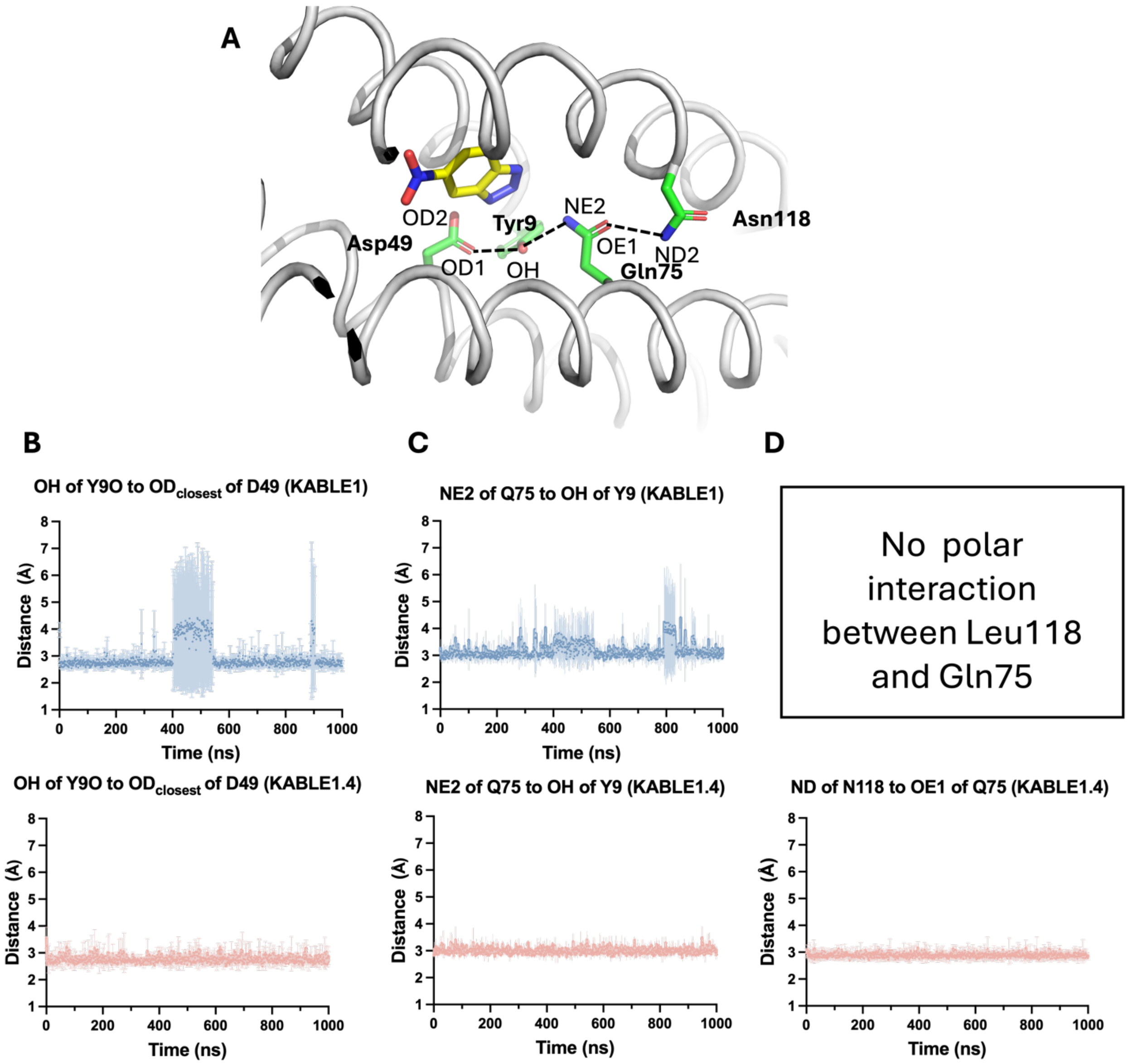

Figure S22. Stabilization of triad by Leu118Asn.

(A) A Representative snap shot of MD simulation of KABLE1.4-6NBT complex showing the triad consisting of Asp49, Tyr9, Gln75 and its stabilization residue Asn118, whose sidechain are shown in green sticks. The hydrogen bonding between these 4 residues are annotated with dotted line and their distance are within 2.8-3.2 Å. **(B-D)** The fluctuation of indicated distances between indicated atoms at the process of 1000ns MD simulation of KABLE1.4-6NBT (pink) complex, in comparison with KABLE1-6NBT (blue) complex. The distances are extracted using the *label bond* function of VMD. The mean of 3 replicates are shown as symbols with dark colors, and the standard deviations are shown as error bars with light colors.

bioRxiv preprint doi: https://doi.org/10.1101/2025.01.30.635804; this version posted January 31, 2025. The copyright holder for this preprint (which was not certified by peer review) is the author/funder, who has granted bioRxiv a license to display the preprint in perpetuity. It is made available under aCC-BY 4.0 International license.

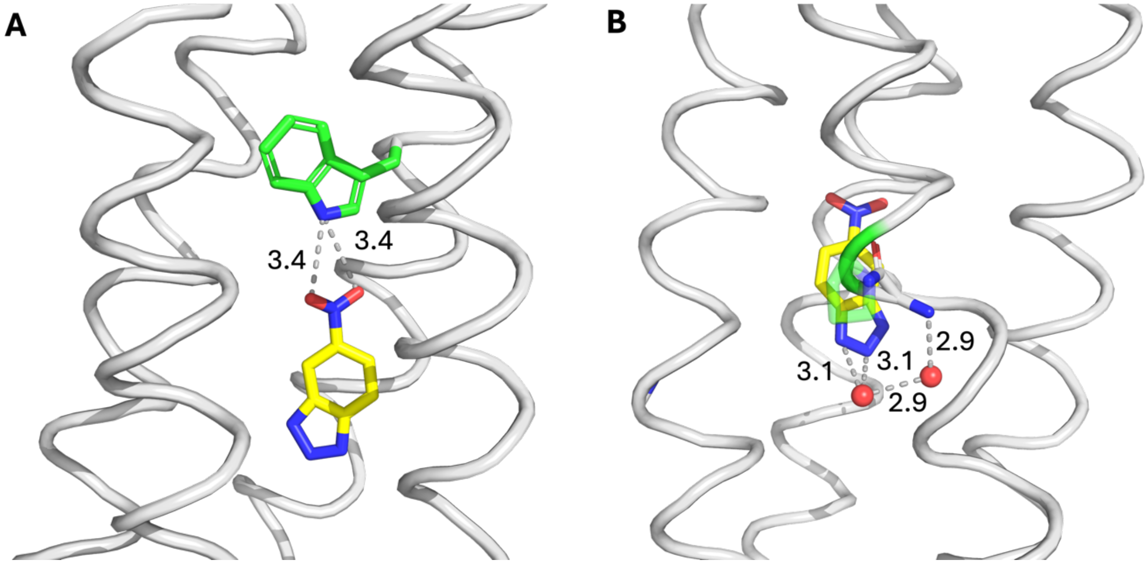

Figure S23: Representative MD snapshots showing the hydrogen bond introduced by Leu20Trp and the active site water introduced by Lys14Pro. **(A)** Representative MD snapshot showing the hydrogen bond between Trp20 and 6NBT. Trp20 is shown in green stick and 6NBT is shown in yellow stick. **(B)** Representative MD snapshot showing the water-mediated interaction to 6NBT, introduced by Pro14-mediated backbone kink. Distances are shown in Å with dashed lines.

bioRxiv preprint doi: https://doi.org/10.1101/2025.01.30.635804; this version posted January 31, 2025. The copyright holder for this preprint (which was not certified by peer review) is the author/funder, who has granted bioRxiv a license to display the preprint in perpetuity. It is made available under aCC-BY 4.0 International license.

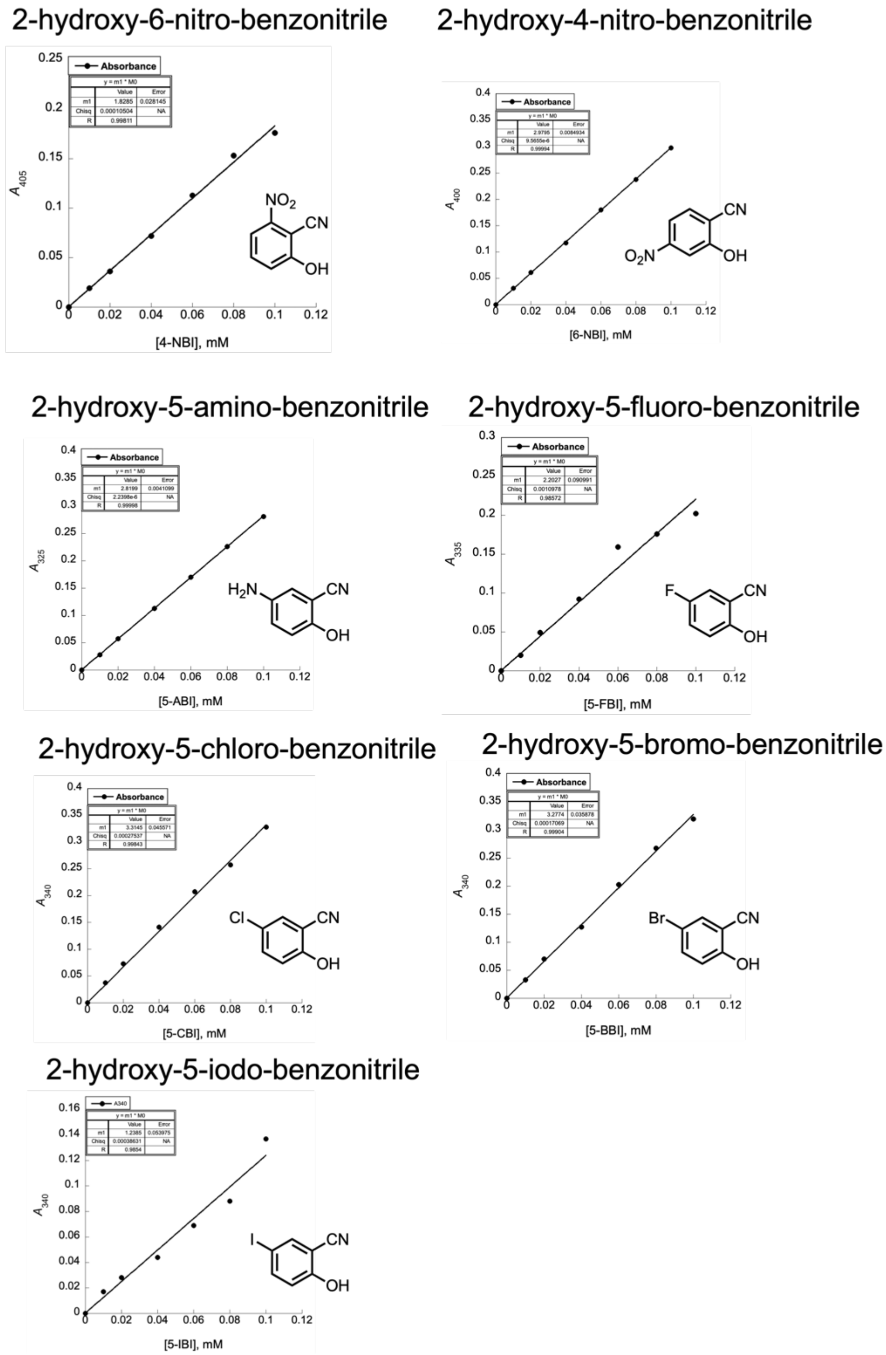

Figure S24. Extinction coefficient determination for products of benzisoxazole substrates using Beer-Lambert law. Data are presented as the mean values and the error bars represent standard deviations obtained from three independent measurements.

bioRxiv preprint doi: https://doi.org/10.1101/2025.01.30.635804; this version posted January 31, 2025. The copyright holder for this preprint (which was not certified by peer review) is the author/funder, who has granted bioRxiv a license to display the preprint in perpetuity. It is made available under aCC-BY 4.0 International license.

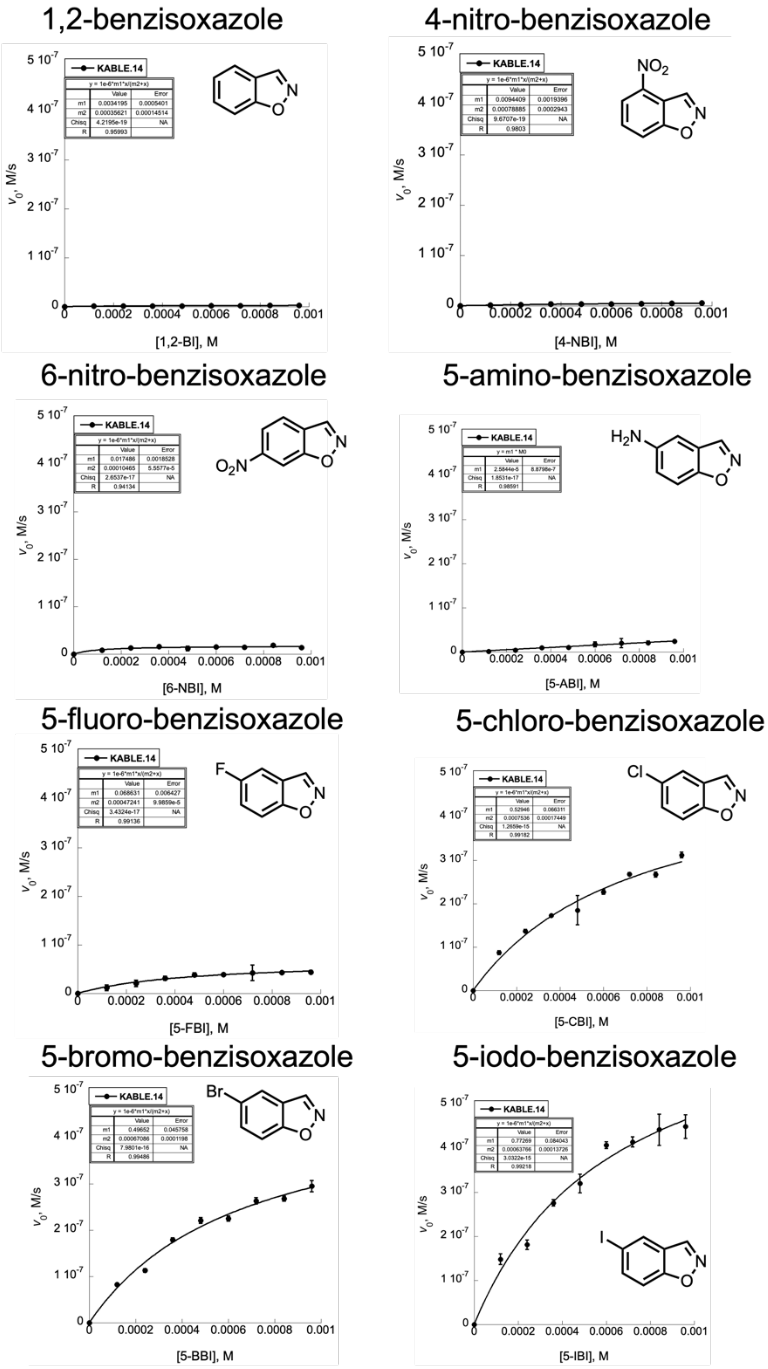

Figure S25. Ring opening of benzisoxazole analogues catalyzed by KABLE1.4. The activity of 1 μM KABLE1.4 was tested with 0.12-0.96 mM substrate in 20 mM TRIS, pH 8.0, 100 mM NaCl (black circle). Data are presented as the mean values and the error bars represent standard deviations obtained from three independent measurements.

bioRxiv preprint doi: https://doi.org/10.1101/2025.01.30.635804; this version posted January 31, 2025. The copyright holder for this preprint (which was not certified by peer review) is the author/funder, who has granted bioRxiv a license to display the preprint in perpetuity. It is made available under aCC-BY 4.0 International license.

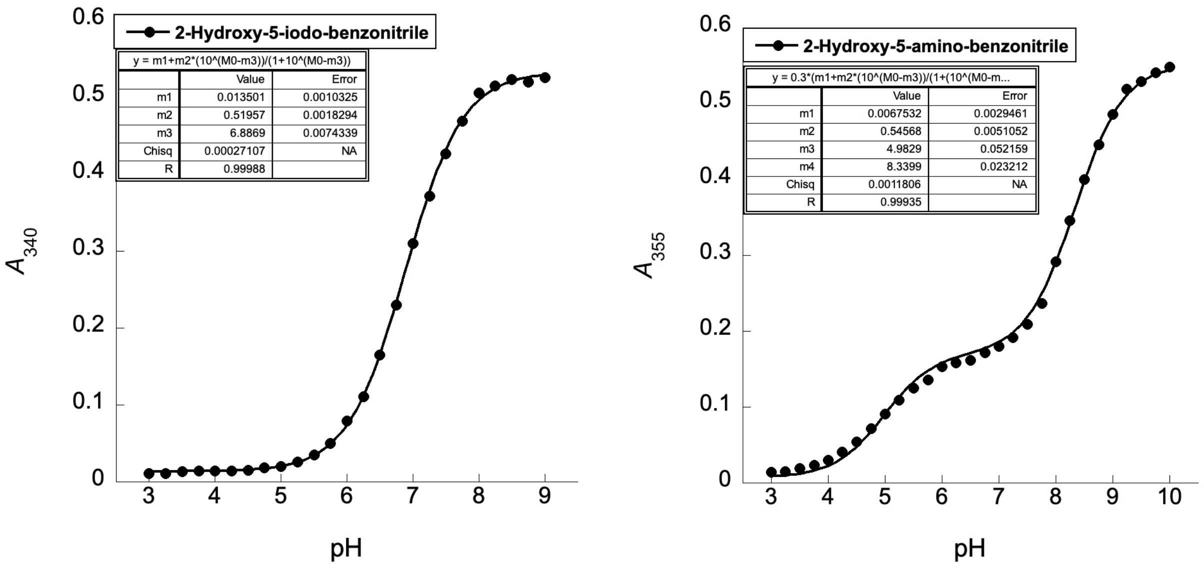

Figure S26. pK_a determination for 2-Hydroxy-5-iodo-benzonitrile (left) and 2-Hydroxy-5-amino-benzonitrile (right) using UV-vis spectrophotometric analyses. Absorbance at wavelength maximum was measured with 100 µM substrate in 100 mM buffer solutions at various pH ranging from 3.0-10.0.

bioRxiv preprint doi: https://doi.org/10.1101/2025.01.30.635804; this version posted January 31, 2025. The copyright holder for this preprint (which was not certified by peer review) is the author/funder, who has granted bioRxiv a license to display the preprint in perpetuity. It is made available under aCC-BY 4.0 International license.

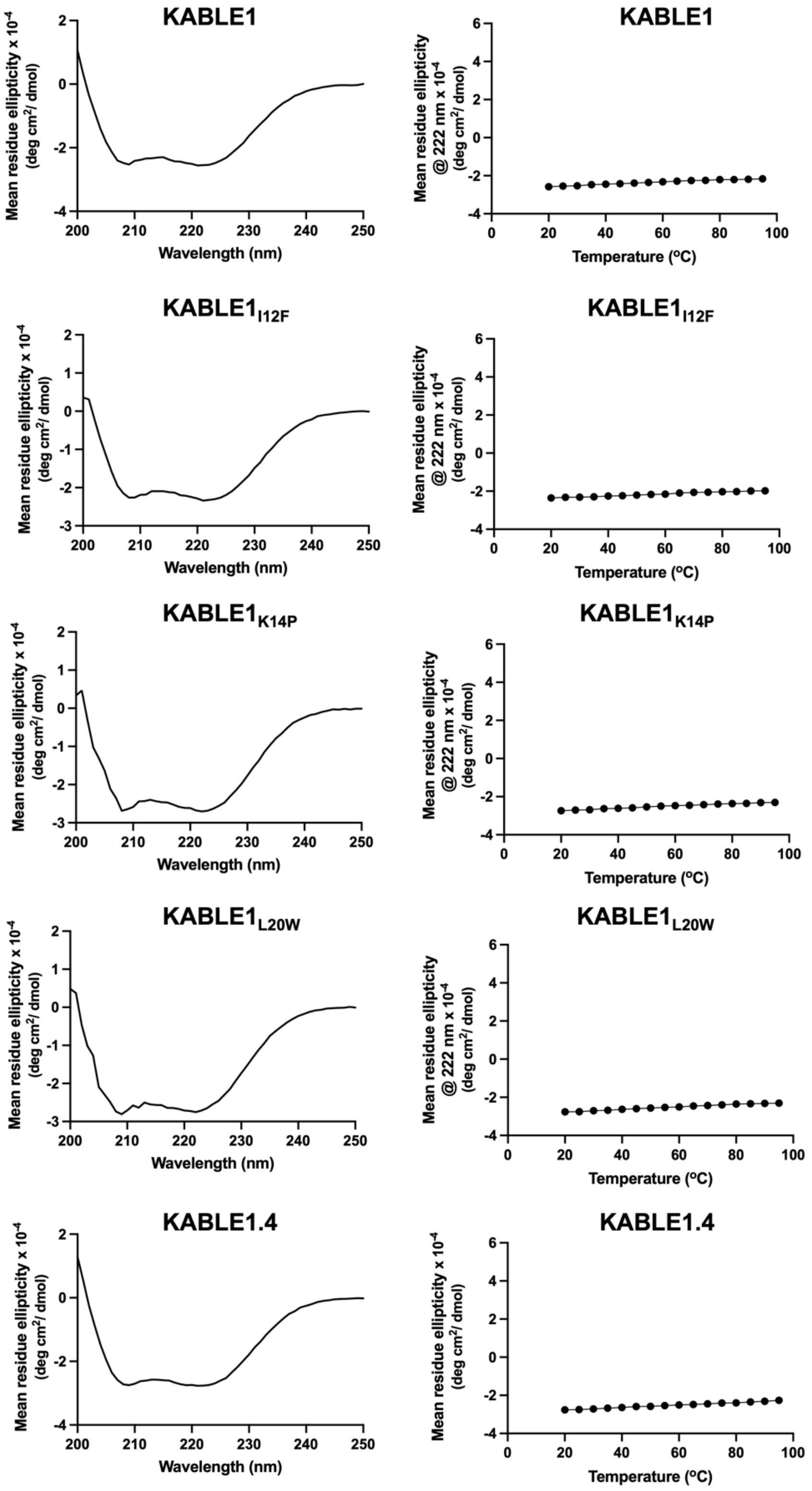

Figure S27. Thermostability of KABLEs. Proteins were prepared in 10 μM concentrations in PBS buffer. CD spectra were collected on a Jasco J-810 CD spectrometer in a 0.1 cm path-length quartz cuvette. Temperature-dependent data were collected at 222 nm from 20 to 95 °C with an interval of 5 °C and an increase rate of 2 °C/minute.

bioRxiv preprint doi: https://doi.org/10.1101/2025.01.30.635804; this version posted January 31, 2025. The copyright holder for this preprint (which was not certified by peer review) is the author/funder, who has granted bioRxiv a license to display the preprint in perpetuity. It is made available under aCC-BY 4.0 International license.

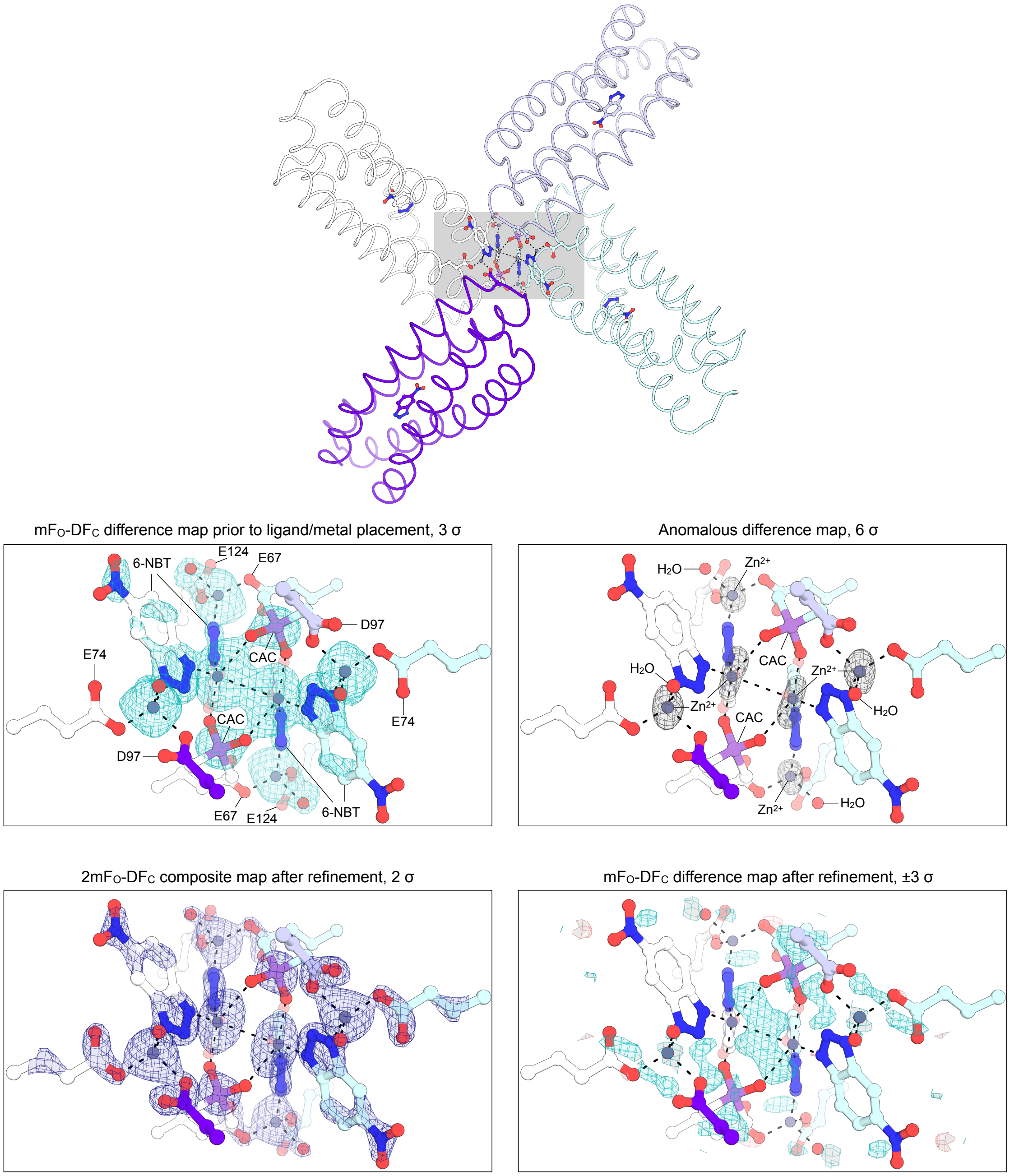

Figure S28. Stabilization of the KABLE12$_{cryst}$ crystal lattice by a 6NBT/zinc/cacodylate complex. The interface between four symmetry-related molecules is shown in the top panel. The gray shaded region is shown in the bottom panels with electron density maps used for assignment and modeling. The modeling of zinc (Zn) complexed by cacodylate (CAC) is supported by the anomalous difference map that shows strong peaks corresponding to the zinc positions and no peaks at the arsenic position, as expected for anomalous scattering for the two elements at 11111 eV. Although all features are supported by the mF_O-DF_C difference map prior to complex modeling and the $2mF_O$-DF_C composite map after refinement, there are residue positive peaks in the mF_O-DF_C difference map after refinement.

bioRxiv preprint doi: https://doi.org/10.1101/2025.01.30.635804; this version posted January 31, 2025. The copyright holder for this preprint (which was not certified by peer review) is the author/funder, who has granted bioRxiv a license to display the preprint in perpetuity. It is made available under aCC-BY 4.0 International license.

Table S1. Ligand efficiency for starting ABLE-apixaban and FABLE-coumarin interactions.

	Protein	Ligand	ΔG (kcal/mol)	#Heavy Atoms	Ligand Efficiency
1	ABLE	Apixaban	-7.23	34	0.213
2	$FABLE_{D1}$[a]	Cou485	-6.21	18	0.345
3	$FABLE_{D2}$	Cou485	-6.19	18	0.344
4	$FABLE_{D3}$	Cou485	-6.02	18	0.334
5	$FABLE_{D4}$	Cou485	-5.56	18	0.309
6	$FABLE_{D5}$	Cou485	-5.70	18	0.317
7	$FABLE_{D1}$[a]	Cou481	-7.14	18	0.357
8	$FABLE_{D1}$[a]	Cou540a	-7.77	18	0.353

[a]$FABLE_{D1}$ was designated as FABLE.

bioRxiv preprint doi: https://doi.org/10.1101/2025.01.30.635804; this version posted January 31, 2025. The copyright holder for this preprint (which was not certified by peer review) is the author/funder, who has granted bioRxiv a license to display the preprint in perpetuity. It is made available under aCC-BY 4.0 International license.

Table S2. Cα RMSD (Å) of FABLE design with AF3 prediction and each monomer of its 3 crystal structures. (PDB: 9DWA, 9DWB, 9DWC)

	Design	9DWA_A	9DWA_B	9DWB_A	9DWB_B	9DWC_A	9DWC_B	9DWC_C	9DWC_D	AF3
Design		0.78	0.79	0.93	0.91	0.91	0.99	1.01	0.93	0.61
9DWA_A	0.78		0.11	0.49	0.53	0.46	0.66	0.66	0.47	0.55
9DWA_B	0.79	0.11		0.51	0.54	0.47	0.67	0.67	0.48	0.57
9DWB_A	0.93	0.49	0.51		0.19	0.32	0.52	0.53	0.34	0.69
9DWB_B	0.91	0.53	0.54	0.19		0.32	0.49	0.50	0.36	0.70
9DWC_A	0.91	0.46	0.47	0.32	0.32		0.43	0.42	0.13	0.74
9DWC_B	0.99	0.66	0.67	0.52	0.49	0.43		0.14	0.44	0.88
9DWC_C	1.01	0.66	0.67	0.53	0.50	0.42	0.14		0.42	0.89
9DWC_D	0.93	0.47	0.48	0.34	0.36	0.13	0.44	0.42		0.78
AF3	0.61	0.55	0.57	0.69	0.70	0.74	0.88	0.89	0.78	

bioRxiv preprint doi: https://doi.org/10.1101/2025.01.30.635804; this version posted January 31, 2025. The copyright holder for this preprint (which was not certified by peer review) is the author/funder, who has granted bioRxiv a license to display the preprint in perpetuity. It is made available under aCC-BY 4.0 International license.

Table S3. Fluorophore library used to probe the specificity of FABLE.

Location	Name	Excitation	Emission	Structure
A1	Coumarin440	350	440	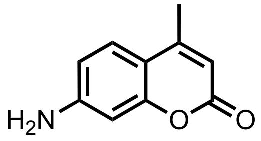
A2	Coumarin445	355	445	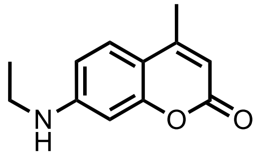
A3	Coumarin450	360	450	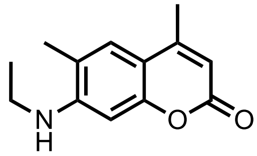
A4	Coumarin456	366	456	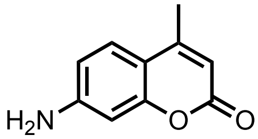
A5	Coumarin460	370	460	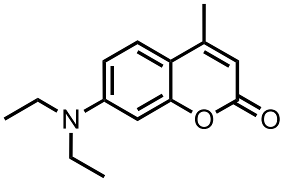
A6	Coumarin461	371	461	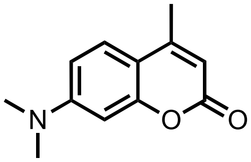
A7	Coumarin466	376	466	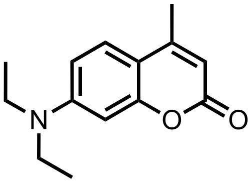

bioRxiv preprint doi: https://doi.org/10.1101/2025.01.30.635804; this version posted January 31, 2025. The copyright holder for this preprint (which was not certified by peer review) is the author/funder, who has granted bioRxiv a license to display the preprint in perpetuity. It is made available under aCC-BY 4.0 International license.

A8	LD473	383	473	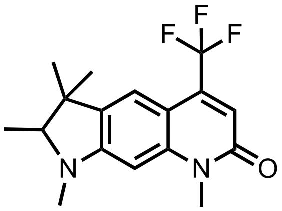
B1	Coumarin478	388	478	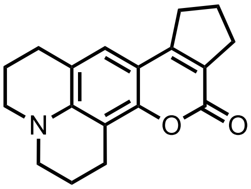
B2	Coumarin480	390	480	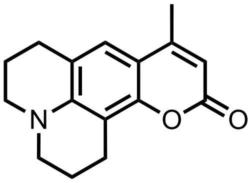
B3	Coumarin481	391	481	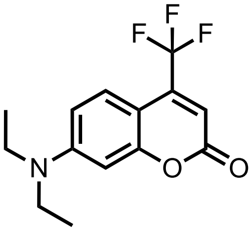
B4	Coumarin485	395	485	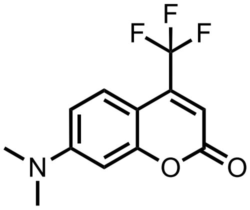
B5	LD489	399	489	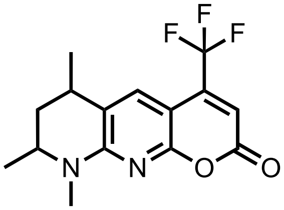
B6	Coumarin490	400	490	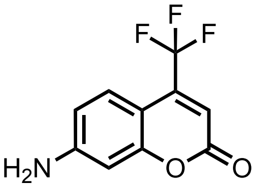
B7	LD490	400	490	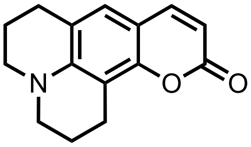

bioRxiv preprint doi: https://doi.org/10.1101/2025.01.30.635804; this version posted January 31, 2025. The copyright holder for this preprint (which was not certified by peer review) is the author/funder, who has granted bioRxiv a license to display the preprint in perpetuity. It is made available under aCC-BY 4.0 International license.

B8	Coumarin498	408	498	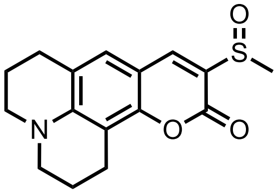
C1	Coumarin500	410	500	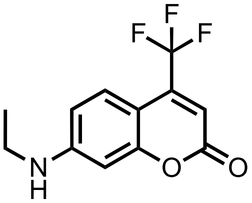
C2	Coumarin503	413	503	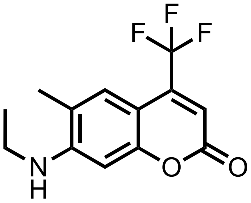
C3	Coumarin504	414	504	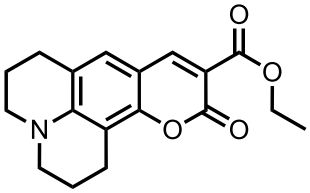
C4	Coumarin510	420	510	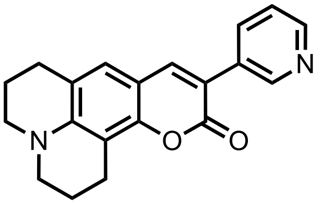
C5	Coumarin515	425	515	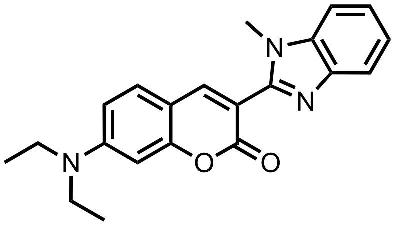
C6	Coumarin519	429	519	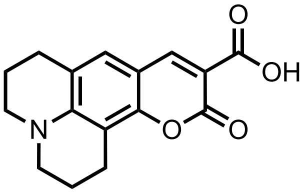
C7	Coumarin521	431	521	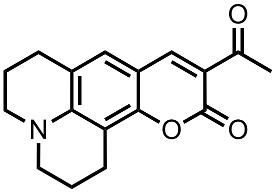

bioRxiv preprint doi: https://doi.org/10.1101/2025.01.30.635804; this version posted January 31, 2025. The copyright holder for this preprint (which was not certified by peer review) is the author/funder, who has granted bioRxiv a license to display the preprint in perpetuity. It is made available under aCC-BY 4.0 International license.

C8	Coumarin523	433	523	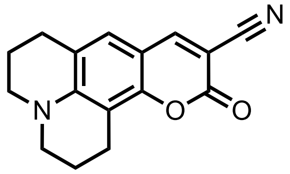
D1	Coumarin525	435	525	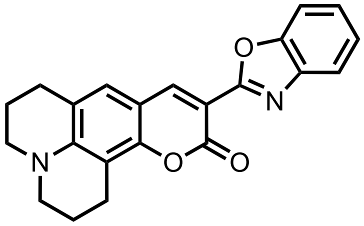
D2	Coumarin535	445	535	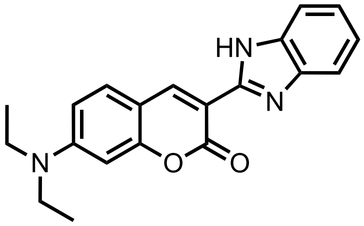
D3	Coumarin545	455	545	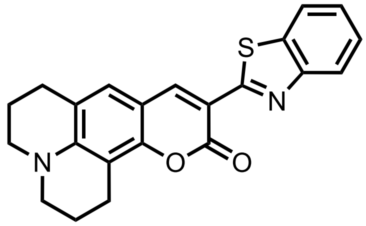
D4	Coumarin540a	460	540a	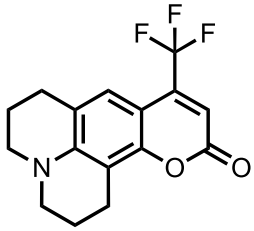
L036	Pyrromethene556	492	518	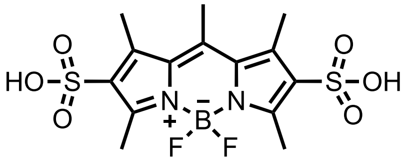
L045	Fluorol555	460	559	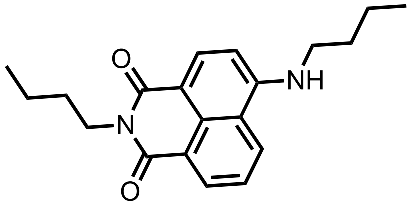
L068	Phenoxazone680	586	670	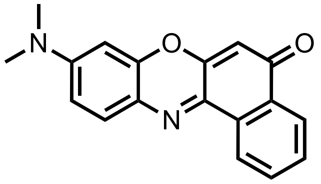

bioRxiv preprint doi: https://doi.org/10.1101/2025.01.30.635804; this version posted January 31, 2025. The copyright holder for this preprint (which was not certified by peer review) is the author/funder, who has granted bioRxiv a license to display the preprint in perpetuity. It is made available under aCC-BY 4.0 International license.

L069	CresylViolet670Perchlorat e	592	628	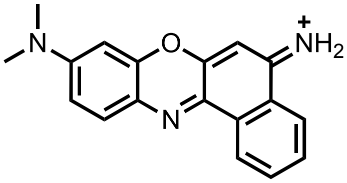
L070	NileBlue690Perchlorate	636	682	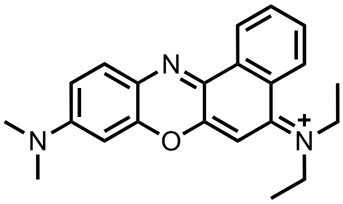
L071	LD690Perchlorate	616	638	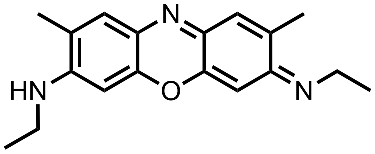
L093	HICl	539	569	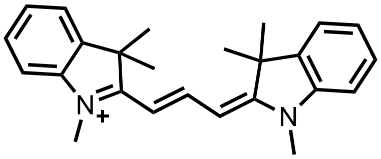
TMR	TMR	550	575	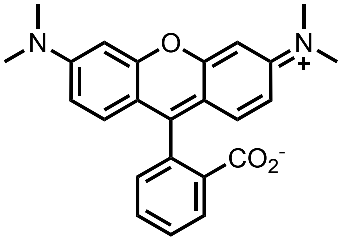

bioRxiv preprint doi: https://doi.org/10.1101/2025.01.30.635804; this version posted January 31, 2025. The copyright holder for this preprint (which was not certified by peer review) is the author/funder, who has granted bioRxiv a license to display the preprint in perpetuity. It is made available under aCC-BY 4.0 International license.

Table S4. Kinetic parameters of KABLEs[a].

**Protein**	**k_{cat}, s^{-1}**	**K_M, mM**	**(k_{cat}/K_M), $M^{-1}s^{-1}$**	**pK_a**
KABLE1	6.6 ± 0.9	0.99 ± 0.29	6,600 ± 2,100	10.01 ± 0.10
KABLE1$_{I12F}$	10.4 ± 0.5	0.99 ± 0.08	10,500 ± 900	9.53 ± 0.53
KABLE1$_{K14P}$[b]	30.0 ± 4.3	2.47 ± 0.10	12,200 ± 1,800	8.08 ± 0.02
KABLE1$_{L20W}$	20.9 ± 0.6	1.33 ± 0.10	15,800 ± 1,300	9.71 ± 0.03
KABLE1$_{L118N}$	17.3 ± 0.6	1.88 ± 0.09	9,200 ± 900	9.85 ± 0.03
KABLE1.2[b]	75 ± 10.7	1.88 ± 0.10	39,900 ± 6,000	8.31 ± 0.03
KABLE1.3[b]	290 ± 41	2.41 ± 0.08	121,000 ± 17,600	8.06 ± 0.03
**KABLE. 1.4[b]**	**800 ± 110**	**1.33 ± 0.05**	**600,000 ± 87,800**	**8.60 ± 0.02**

[a]Error bars represent the standard errors of the mean of 3 independent measurments. (See **section 1.7** of SI for details)
[b]Kinetic parameters are from the R^2 analysis of global fitting. (See **section 1.7** of SI for details)

bioRxiv preprint doi: https://doi.org/10.1101/2025.01.30.635804; this version posted January 31, 2025. The copyright holder for this preprint (which was not certified by peer review) is the author/funder, who has granted bioRxiv a license to display the preprint in perpetuity. It is made available under aCC-BY 4.0 International license.

Table S5. Protein concentrations (nM) used in kinetic assay of Kemp eliminase.

pH	KABLE1	KABLE1 I12F	KABLE1 K14P	KABLE1 L20W	KABLE1 L118N	KABLE1.2	KABLE1.3	KABLE1.4
6		1000		1000	1000	100	100	5
6.5		1000	100	1000	1000	100	100	5
7	1000	1000	100	1000	1000	10	10	5
7.5	1000	1000	100	1000	1000	10	10	5
8	1000	1000	100	1000	1000	10	10	5
8.5	1000	1000	100	1000	1000	10	10	5
9	100	100	10	100	100	10	10	5
9.5	100	100		100	100	10	10	5
10	100	100		100	100	10	10	5

bioRxiv preprint doi: https://doi.org/10.1101/2025.01.30.635804; this version posted January 31, 2025. The copyright holder for this preprint (which was not certified by peer review) is the author/funder, who has granted bioRxiv a license to display the preprint in perpetuity. It is made available under aCC-BY 4.0 International license.

Table S6. Ca RMSD of designed KABLE1, Chai1 prediction of 6NBT-bound structures, and each monomer of its 2 crystal structures. (PDB: 9N0I,9N0J)

	9N0I_A	9N0I_B	9N0I_C	9N0I_D	9N0I_E	9N0I_F	9N0J_A	KABLE1_Chai1	KABLE12cryst_Chai1	KABLE1_Design
9N0I_A		0.5	0.4	0.6	0.5	0.6	0.6	0.7	0.6	0.8
9N0I_B	0.5		0.3	0.5	0.5	0.6	0.4	0.7	0.6	0.9
9N0I_C	0.4	0.3		0.4	0.5	0.5	0.4	0.7	0.5	0.8
9N0I_D	0.6	0.5	0.4		0.3	0.3	0.6	0.7	0.4	1.1
9N0I_E	0.5	0.5	0.5	0.3		0.4	0.7	0.7	0.5	1.0
9N0I_F	0.6	0.6	0.5	0.3	0.4		0.7	0.7	0.4	1.1
9N0J_A	0.6	0.4	0.4	0.6	0.7	0.7		0.8	0.7	0.9
KABLE1_Chai1	0.7	0.7	0.7	0.7	0.7	0.7	0.8		0.5	0.7
KABLE12cryst_Chai1	0.6	0.6	0.5	0.4	0.5	0.4	0.7	0.5		0.9
KABLE1_Design	0.8	0.9	0.8	1.1	1.0	1.1	0.9	0.7	0.9	

bioRxiv preprint doi: https://doi.org/10.1101/2025.01.30.635804; this version posted January 31, 2025. The copyright holder for this preprint (which was not certified by peer review) is the author/funder, who has granted bioRxiv a license to display the preprint in perpetuity. It is made available under aCC-BY 4.0 International license.

Table S7. Catalytic efficiency of KABLE1.4 for benzisoxazole derivative at pH 8.0 with the extinction coefficient at the wavelength maximum. pK_a values for salicylonitriles are listed at 30 ºC unless stated otherwise.

**Benzisoxazole**	**pK_a of salicylonitrile**[a]	**λ_{max} (water)**[c]**, nm (ϵ)**	**Catalytic efficiency, $M^{-1}s^{-1}$**
1,2-benzisoxazole	6.9	325 (5,056)[d]	9 ± 4
4-nitrobenzisoxazole	5.2	405 (1,829)	12 ± 5
5-nitrobenzisoxazole	4.1	380 (15,800)	(2.32 ± 0.42) x 10^5
6-nitrobenzisoxazole	5.2	400 (2,980)	175 ± 107
5-aminobenzisoxazole	5.0, 8.3[b]	325 (2,820)	26 ± 1
5-fluorobenzisoxazole	6.8	335 (2,203)	145 ± 33
5-chlorobenzisoxazole	6.4	340 (3,315)	707 ± 191
5-bromobenzisoxazole	5.8	340 (3,277)	746 ± 157
5-iodobenzisoxazole	6.9[b]	340 (1,239)	1222 ± 293

[a]measured at 30 ºC unless otherwise mentioned (*69*).

[b]measured at 22 ºC in this research.

[c]measured in this research otherwise mentioned.

[d]measured at reference (*68*).

bioRxiv preprint doi: https://doi.org/10.1101/2025.01.30.635804; this version posted January 31, 2025. The copyright holder for this preprint (which was not certified by peer review) is the author/funder, who has granted bioRxiv a license to display the preprint in perpetuity. It is made available under aCC-BY 4.0 International license.

Table S8. Kinetic parameters of reported proteins catalyzing the Kemp elimination of 5-nitrobenzisoxazole with base-mediated mechanism.

Protein	Description	k_{cat}/K_M, $M^{-1}s^{-1}$	k_{cat}, s^{-1}	K_M, mM	Amino acid efficiency[a]	Ref
34E4	Catalytic antibody	5500	0.66	1.2	26	(*72*)
BSA	Natural lipid carrier	6500	6.02	ND	11	(*109*)
KE07	Computational redesign	12.2	0.018	1.4	0.05	(*29*)
KE59	Computational redesign	163	0.29	1.8	0.66	(*29*)
KE70	Computational redesign	78	0.16	2.1	0.31	(*29*)
KE07 R7 10/11G	7 round DE of KE07	2590	1.37	0.54	10	(*66*)
KE59 R13_3/11H	13 round DE of KE59	60,430	9.53	0.16	240	(*68*)
KE70 R8 15/11E	8 round DE of KE70	34,900	5.3	0.15	140	(*110*)
HG3	Computational redesign and rational engineering	425	0.68	1.6	1.4	(*32*)
HG3.17	17 round DE of HG3	150,000	604	3.5	500	(*30*, *31*)
HG3.R5	5 round DE of HG3	170,000	702	4.8	560	(*31*)
HG4	Ensemble-based redesign of HG3	103,000	ND	ND	340	(*35*)
HG649	Ensemble-based redesign of HG3	32,000	ND	ND	110	(*107*)
AlleyCat	Single mutation of Calmodulin	5.8	ND	ND	0.08	(*33*)
AlleyCat10	NMR-guided DE of AlleyCat7	4378	21.2	4.8	59	(*34*)
Lysozyme L99A/M102H	Double mutation of Lysozyme	1.8	ND	ND	0.01	(*111*)
GNCA4 W229D F290W	Double mutation of GNCA4	5497	10	ND	21	(*36*)
V4	DE of GNCA4 W229D F290W	200,000	635	3.14	710	(*112*)
KE15	Computational redesign	35	0.022	0.63	0.14	(*29*)
KE15 Tyr167Lys+ R4	Electrical field-guided design of KE15	403	0.31	0.77	1.6	(*63*)
tKSI	Natural enzyme	2.5	ND	ND	0.02	(*60*)

bioRxiv preprint doi: https://doi.org/10.1101/2025.01.30.635804; this version posted January 31, 2025. The copyright holder for this preprint (which was not certified by peer review) is the author/funder, who has granted bioRxiv a license to display the preprint in perpetuity. It is made available under aCC-BY 4.0 International license.

D38N tKSI	Single mutation of tKSI	17,000	ND	ND	140	(*60*)
Des27	Computational design	130	0.07	0.5	0.52	(*61*)
Des27.7	Computational redesign	12,700	2.85	0.21	51	(*61*)
F113L Des27.7	Single mutation on Des27.7	123,000	30	ND	490	(*61*)
**KABLE1**	De Novo Design	**6,600**	**6.6**	**0.99**	**57**	This work
**KABLE1.4**	Quadruple mutant of KABLE1	**600,000**	**800**	**1.33**	**4,800**	This work

[a]Amino acid catalytic efficiency, which is the quotient of catalytic efficiency (k_{cat}/K_M, $M^{-1}s^{-1}$), by the total number of amino acid for each proteins.

S9. Primer sequences used in this study.

Primer	Sequence
T7	TAATACGACTCACTATAGGG
T7_term	GCTAGTTATTGCTCAGCGG
Y9NNK	AAGTTTGAGGAG**NNK**GAGAAAATCGGG
I12NNK	GAGTATGAGAAA**NNK**GGGAAGCGTATT
K14NNK	GAGAAAATCGGG**NNK**CGTATTCTCGAA
I16NNK	ATCGGGAAGCGT**NNK**CTTGAACTGTTA
L17NNK	GGGAAGCGTATT**NNK**GAACTGTTACAG
L20NNK	ATTCTCGAACTG**NNK**CAGGAAGCCCGT
L42NNK	TTACTTCGGGAA**NNK**AAGGAGCTTTTT
F46NNK	TTAAAGGAGCTT**NNK**AAGAAGGATCTC
L50NNK	TTTAAGAAGGAT**NNK**AAGTTAGCTGAA
L52NNK	TTTAAGAAGGATCTCAAG**NNK**GCTGAA
Q75NNK	CTGCTGGAAGAA**NNK**CTCGAACGTCTC
L79NNK	CAGCTCGAACGT**NNK**AAGAAAATCCAG
I82NNK	CGTCTCAAGAAA**NNK**CAGGCGATGTTC
G112NNK	ATCATCGAGGAG**NNK**AAAGCACTTTTA
K113NNK	ATCGAGGAGGGG**NNK**GCACTTTTACCT
L115NNK	GAGGGGAAAGCA**NNK**TTACCTCTTGTG
L116NNK	GGGAAAGCACTT**NNK**CCTCTTGTGGAG
P117NNK	GGGAAAGCACTTTTA**NNK**CTTGTGGAG
L118NNK	GCACTTTTACCT**NNK**GTGGAGAAGATC
V119NNK	CTTTTACCTCTT**NNK**GAGAAGATCAAA

bioRxiv preprint doi: https://doi.org/10.1101/2025.01.30.635804; this version posted January 31, 2025. The copyright holder for this preprint (which was not certified by peer review) is the author/funder, who has granted bioRxiv a license to display the preprint in perpetuity. It is made available under aCC-BY 4.0 International license.

Table S10. Amino acid sequences of designed proteins and their mutants.

Protein	Amino acid sequence	DNA Sequence
FABLE	MHHHHHHENLYFQSIKSEFAEGAAVFVEGVAVFLTMMAAFQNGDKEAVAQYLARGASLYTRHEELLNRLLQKLRREGNKEAVTLMNEFTATFQTGKSLANALIAAFKNGDDDSFESYLQAIWKVIAKMATILDQIAKAI	ATGCACCACCACCACCACCACGAAAACCTGTACTTCCAGTCAATCAAGAGCGAGTTCGCCGAGGGGGCGGCTGTATTTGTAGAAGGCGTCGCCGTGTTTCTGACCATGATGGCCGCATTTCAGAACGGCGATAAGGAGGCTGTCGCACAGTACCTCGCGCGGGGGGCTTCCCTGTACACCCGTCACGAGGAGTTGCTGAACCGGCTTTTACAAAAGTTGCGGCGCGAAGGGAACAAAGAAGCAGTGACTCTTATGAACGAATTTACCGCCACTTTTCAAACTGGTAAGTCCTTAGCTAATGCTCTGATCGCGGCATTCAAAAATGGTGACGATGACTCCTTTGAAAGTTACCTTCAAGCAATCTGGAAGGTCATCGCCAAGATGGCAACTATTTTGGATCAAATTGCAAAAGCAATT
KABLE0	MHHHHHHENLYFQSLKELFEEWLEVRKKIDEVFDAMKAAFEAGDMKTVGEYIKELKELYKEYLAMLEELLRRARALGLEEAVALLEEYKGLVEEASKAFEAAVKAFEAGDWAAFKKYLEKEEEIRKKMEPLVKALEEAL	ATGCACCACCACCACCACCACGAAAACCTGTACTTCCAGAGCTTAAAAGAGTTATTTGAAGAGTGGTTGGAAGTTCGTAAGAAGATTGATGAAGTATTCGATGCAATGAAGGCCGCTTTCGAGGCCGGGGATATGAAAACAGTGGGGGAGTATATCAAAGAATTGAAGGAACTTTATAAAGAATACTTAGCTATGCTTGAGGAGTTGCTCCGGCGTGCTCGTGCCTTAGGTCTTGAAGAGGCAGTCGCATTACTGGAAGAGTACAAAGGGTTAGTCGAGGAGGCATCGAAGGCTTTTGAAGCTGCCGTAAAAGCTTTCGAGGCTGGGGACTGGGCCGCCTTCAAAAAATACCTTGAGAAGGAAGAGGAGATCCGCAAAAAGATGGAACCGCTCGTCAAAGCCCTGGAAGAGGCACTC
KABLE1	MHHHHHHENLYFQSSLKEKFEEYEKIGKRILELLQEARDAFEAGDLARVDELLRELKELFKKDLKLAEEMKKEAEEAGNKEAVELLEEQLERLKKIQAMFEEAVEAFRAGDRERFGELLEKIIEEGKALLPLVEKIKEAI	ATGCACCACCACCACCACCACGAAAACCTGTACTTCCAGAGCAGCCTCAAAGAAAAGTTTGAGGAGTATGAGAAAATCGGGAAGCGTATTCTCGAACTGTTACAGGAAGCCCGTGACGCTTTCGAGGCTGGTGATCTCGCGCGGGTTGATGAGTTACTTCGGGAATTAAAGGAGCTTTTTAAGAAGGATCTCAAGTTAGCTGAAGAA

bioRxiv preprint doi: https://doi.org/10.1101/2025.01.30.635804; this version posted January 31, 2025. The copyright holder for this preprint (which was not certified by peer review) is the author/funder, who has granted bioRxiv a license to display the preprint in perpetuity. It is made available under aCC-BY 4.0 International license.

		ATGAAAAAGGAAGCGGAAGAAGC<br>TGGGAATAAAGAGGCGGTGGAGC<br>TGCTGGAAGAACAGCTCGAACGT<br>CTCAAGAAAATCCAGGCGATGTT<br>CGAGGAGGCAGTTGAGGCGTTTC<br>GGGCCGGGGACCGCGAGCGTTTT<br>GGTGAGCTCCTCGAAAAGATCAT<br>CGAGGAGGGGAAAGCACTTTTAC<br>CTCTTGTGGAGAAGATCAAAGAG<br>GCTATC
KABLE1.2	MHHHHHHENLYFQSSLKEKFEEYEKIGPRI<br>LELWQEARDAFEAGDLARVDELLRELKELF<br>KKDLKLAEEMKKEAEEAGNKEAVELLEEQL<br>ERLKKIQAMFEEAVEAFRAGDRERFGELLE<br>KIIEEGKALLPLVEKIKEAI	ATGCACCACCACCACCACCACGA<br>AAACCTGTACTTCCAGAGCAGCC<br>TCAAAGAAAAGTTTGAGGAGTAT<br>GAGAAAATCGGGCCGCGTATTCT<br>TGAACTGTGGCAGGAAGCCCGTG<br>ACGCTTTCGAGGCTGGTGATCTC<br>GCGCGGGTTGATGAGTTACTTCG<br>GGAATTAAAGGAGCTTTTTAAGA<br>AGGATCTCAAGTTAGCTGAAGAA<br>ATGAAAAAGGAAGCGGAAGAAGC<br>TGGGAATAAAGAGGCGGTGGAGC<br>TGCTGGAAGAACAGCTCGAACGT<br>CTCAAGAAAATCCAGGCGATGTT<br>CGAGGAGGCAGTTGAGGCGTTTC<br>GGGCCGGGGACCGCGAGCGTTTT<br>GGTGAGCTCCTCGAAAAGATCAT<br>CGAGGAGGGGAAAGCACTTTTAC<br>CTCTTGTGGAGAAGATCAAAGAG<br>GCTATC
KABLE1.2$_{cryst}$	MHHHHHHENLYFQSSLKEKFAEYEAIGPRI<br>LELWQAARNAFEAGDLARVANLLAELKELF<br>KKDLNLANAMAAEAAEAGNKEAVALLAEQL<br>ERLKKIQAMFAAAVNAFRAGDREAFGALLE<br>AIINEGKALLPLVEAIKEAI	ATGCACCACCACCACCACCACGA<br>AAACCTGTACTTCCAGAGCTCAC<br>TGAAGGAGAAATTCGCCGAATAC<br>GAGGCGATTGGGCCGCGGATTCT<br>CGAATTGTGGCAGGCAGCGCGTA<br>ATGCTTTCGAAGCTGGGGATCTC<br>GCTCGGGTTGCCAATTTGCTTGC<br>GGAATTGAAGGAACTGTTCAAGA<br>AAGACCTGAATCTTGCGAACGCA<br>ATGGCGGCCGAGGCGGCGGAAGC<br>CGGGAATAAGGAAGCTGTAGCCC<br>TTTTGGCGGAACAGCTTGAGCGG<br>TTAAAGAAAATCCAGGCAATGTT<br>CGCCGCGGCGGTGAATGCTTTCC<br>GGGCGGGGGACCGCGAGGCATTT<br>GGTGCATTGTTGGAGGCAATCAT<br>CAACGAAGGCAAGGCTCTCTTAC<br>CGCTCGTGGAGGCGATTAAGGAG<br>GCCATC
KABLE1.3	MHHHHHHENLYFQSSLKEKFEEYEKFGPRI<br>LELWQEARDAFEAGDLARVDELLRELKELF<br>KKDLKLAEEMKKEAEEAGNKEAVELLEEQL<br>ERLKKIQAMFEEAVEAFRAGDRERFGELLE<br>KIIEEGKALLPLVEKIKEAI	ATGCACCACCACCACCACCACGA<br>AAACCTGTACTTCCAGAGCAGCC<br>TCAAAGAAAAGTTTGAGGAGTAT<br>GAGAAATTTGGGCCGCGTATTCT<br>TGAACTGTGGCAGGAAGCCCGTG<br>ACGCTTTCGAGGCTGGTGATCTC

bioRxiv preprint doi: https://doi.org/10.1101/2025.01.30.635804; this version posted January 31, 2025. The copyright holder for this preprint (which was not certified by peer review) is the author/funder, who has granted bioRxiv a license to display the preprint in perpetuity. It is made available under aCC-BY 4.0 International license.

		GCGCGGGTTGATGAGTTACTTCG GGAATTAAAGGAGCTTTTTAAGA AGGATCTCAAGTTAGCTGAAGAA ATGAAAAAGGAAGCGGAAGAAGC TGGGAATAAAGAGGCGGTGGAGC TGCTGGAAGAACAGCTCGAACGT CTCAAGAAAATCCAGGCGATGTT CGAGGAGGCAGTTGAGGCGTTTC GGGCCGGGGACCGCGAGCGTTTT GGTGAGCTCCTCGAAAAGATCAT CGAGGAGGGGAAAGCACTTTTAC CTCTTGTGGAGAAGATCAAAGAG GCTATC
KABLE1.4	MHHHHHHENLYFQSSLKEKFEEYEKFGPRI LELWQEARDAFEAGDLARVDELLRELKELF KKDLKLAEEMKKEAEEAGNKEAVELLEEQL ERLKKIQAMFEEAVEAFRAGDRERFGELLE KIIEEGKALLPNVEKIKEAI	ATGCACCACCACCACCACCACGA AAACCTGTACTTCCAGAGCAGCC TCAAAGAAAAGTTTGAGGAGTAT GAGAAATTTGGGCCGCGTATTCT TGAACTGTGGCAGGAAGCCCGTG ACGCTTTCGAGGCTGGTGATCTC GCGCGGGTTGATGAGTTACTTCG GGAATTAAAGGAGCTTTTTAAGA AGGATCTCAAGTTAGCTGAAGAA ATGAAAAAGGAAGCGGAAGAAGC TGGGAATAAAGAGGCGGTGGAGC TGCTGGAAGAACAGCTCGAACGT CTCAAGAAAATCCAGGCGATGTT CGAGGAGGCAGTTGAGGCGTTTC GGGCCGGGGACCGCGAGCGTTTT GGTGAGCTCCTCGAAAAGATCAT CGAGGAGGGGAAAGCACTTTTAC CTAACGTGGAGAAGATCAAAGAG GCTATC

bioRxiv preprint doi: https://doi.org/10.1101/2025.01.30.635804; this version posted January 31, 2025. The copyright holder for this preprint (which was not certified by peer review) is the author/funder, who has granted bioRxiv a license to display the preprint in perpetuity. It is made available under aCC-BY 4.0 International license.

Supplemental text

Computational design of FABLE_RosettaScript(.xml)

```
<ROSETTASCRIPTS>
  <SCOREFXNS>
    <ScoreFunction name="ligand_soft_rep" weights="ligand_soft_rep" />
    <ScoreFunction name="hard_rep" weights="ligandprime" />
    <ScoreFunction name="ref15" weights="ref2015">
      <Reweight scoretype="atom_pair_constraint" weight="5"/>
    </ScoreFunction>
    <ScoreFunction name="ref15_1" weights="ref2015">
      <Reweight scoretype="aa_composition" weight="1" />
      <Reweight scoretype="netcharge" weight="1.0" />
      <Reweight scoretype="atom_pair_constraint" weight="5"/>
      <Set aa_composition_setup_file="helix_composition.comp" />
      <Set netcharge_setup_file="helix_netcharge.charge" />
    </ScoreFunction>
  </SCOREFXNS>
  <RESIDUE_SELECTORS>
  </RESIDUE_SELECTORS>
  <TASKOPERATIONS>
    <InitializeFromCommandline name="ifcl"/>
    <ReadResfile name="resfile" filename="resfile_topology.txt"/>
    <ExtraRotamersGeneric name="extrachi" ex1="1" ex2="1" ex1_sample_level="1" ex2_sample_level="1"
extrachi_cutoff="14"/>
    <DetectProteinLigandInterface name="design_interface" cut1="6.0" cut2="8.0" cut3="10.0" cut4="12.0"
design="1" resfile="resfile_topology.txt" />
    IncludeCurrent name="include_curr" />
  </TASKOPERATIONS>
  <FILTERS>
    <PackStat name="pstat" confidence="0" threshold="0" repeats="10"/>
    <PackStat name="pstat_mc" threshold="0" repeats="10"/>
    <NetCharge name="net_charge" confidence="0"/>
    <LigInterfaceEnergy name="ligen"  scorefxn="ref15" include_cstE="0" energy_cutoff="0" confidence="0"/>
    <ScoreType name="total_score_1" scorefxn="ref15_1" score_type="total_score" threshold="0"/>
    <ScoreType name="total_score" scorefxn="ref15" score_type="total_score" threshold="0"/>
    <BuriedUnsatHbonds name="bu" report_sc_heavy_atom_unsats="true" scorefxn="ref15" cutoff="0"
residue_surface_cutoff="20.0" ignore_surface_res="true" print_out_info_to_pdb="true" confidence="0" />
  </FILTERS>
  <LIGAND_AREAS>
    <LigandArea name="docking_sidechain" chain="X" cutoff="6.0" add_nbr_radius="true"
all_atom_mode="true" minimize_ligand="10" />
    <LigandArea name="final_sidechain" chain="X" cutoff="6.0" add_nbr_radius="true" all_atom_mode="true"
/>
    <LigandArea name="final_backbone" chain="X" cutoff="7.0" add_nbr_radius="false" all_atom_mode="true"
Calpha_restraints="0.3" />
  </LIGAND_AREAS>
  <INTERFACE_BUILDERS>
    <InterfaceBuilder name="side_chain_for_docking" ligand_areas="docking_sidechain" />
    <InterfaceBuilder name="side_chain_for_final" ligand_areas="final_sidechain" />
    <InterfaceBuilder name="backbone" ligand_areas="final_backbone" extension_window="3" />
  </INTERFACE_BUILDERS>
  <MOVEMAP_BUILDERS>
```

bioRxiv preprint doi: https://doi.org/10.1101/2025.01.30.635804; this version posted January 31, 2025. The copyright holder for this preprint (which was not certified by peer review) is the author/funder, who has granted bioRxiv a license to display the preprint in perpetuity. It is made available under aCC-BY 4.0 International license.

```
        <MoveMapBuilder name="docking" sc_interface="side_chain_for_docking" minimize_water="true" />
        <MoveMapBuilder name="final" sc_interface="side_chain_for_final" bb_interface="backbone"
minimize_water="true" />
    </MOVEMAP_BUILDERS>
    <SCORINGGRIDS ligand_chain="X" width="46">
        <ClassicGrid grid_name="vdw" weight="1.0" />
    </SCORINGGRIDS>
    <MOVERS>
        <ConstraintSetMover name="atomic" cst_file="constraints_helix.cst" />
        FavorNativeResidue name="favor_native" bonus="0.00" />
        <Transform name="transform" chain="X" box_size="10.0" move_distance="0.1" angle="5" cycles="500"
repeats="1" temperature="5" rmsd="4.0" use_constraints="true" cst_fa_file="constraints_helix.cst"
cst_fa_weight="5" />
        HighResDocker name="high_res_docker" cycles="6" repack_every_Nth="3" scorefxn="ligand_soft_rep"
movemap_builder="docking" />
        <PackRotamersMover name="design_interface" scorefxn="hard_rep" task_operations="design_interface" />
        FinalMinimizer name="final" scorefxn="hard_rep" movemap_builder="final" />
        <InterfaceScoreCalculator name="add_scores" chains="X" scorefxn="hard_rep" />
        <ParsedProtocol name="low_res_dock">
            <Add mover_name="transform" />
        </ParsedProtocol>
        ParsedProtocol name="high_res_dock">
            Add mover_name="high_res_docker" />
            Add mover_name="final" />
        /ParsedProtocol>
        <PackRotamersMover name="fixbb_aro" scorefxn="ref15_1" task_operations="resfile,extrachi,ifcl"/>
        <PackRotamersMover name="pack" scorefxn="ref15_1" task_operations="ifcl,resfile,extrachi"/>
        <PackRotamersMover name="pack_fast" scorefxn="ref15_1" task_operations="ifcl,resfile"/>
        <MinMover name="min_bb" scorefxn="ref15" tolerance="0.0000001" max_iter="1000" chi="false"
bb="true">
            <MoveMap name="map_bb">
                <Span begin="1" end="200" bb="true" chi="false" />
                <Span begin="201" end="999" bb="false" chi="false"/>
            </MoveMap>
        </MinMover>
        <Idealize name="idealize"/>
        <MinMover name="min_sc" scorefxn="ref15" tolerance="0.0000001" max_iter="1000" chi="true"
bb="false">
            <MoveMap name="map_sc">
                <Span begin="1" end="200" bb="false" chi="true" />
                <Span begin="201" end="999" bb="false" chi="false"/>
            </MoveMap>
        </MinMover>
        <MinMover name="min_sc_bb" scorefxn="ref15" tolerance="0.0000001" max_iter="1000" chi="true"
bb="true">
            <MoveMap name="map_sc_bb">
                <Span begin="1" end="200" bb="true" chi="true" />
                <Span begin="201" end="999" bb="false" chi="false"/>
            </MoveMap>
        </MinMover>
        <ParsedProtocol name="parsed_pack_fast" >
            <Add mover_name="pack_fast"/>
            <Add mover_name="min_bb"/>
        </ParsedProtocol>
        <ParsedProtocol name="parsed_pack" >
            <Add mover_name="pack"/>
```

bioRxiv preprint doi: https://doi.org/10.1101/2025.01.30.635804; this version posted January 31, 2025. The copyright holder for this preprint (which was not certified by peer review) is the author/funder, who has granted bioRxiv a license to display the preprint in perpetuity. It is made available under aCC-BY 4.0 International license.


```
            <Add mover_name="min_bb"/>
            <Add mover_name="min_sc"/>
        </ParsedProtocol>
        <GenericMonteCarlo name="pack_mc" preapply="0" trials="3" temperature="0.03" filter_name="pstat_mc"
sample_type="high" mover_name="parsed_pack">
                <Filters>
                    <AND filter_name="total_score_1" temperature="15" sample_type="low"/>
                </Filters>
        </GenericMonteCarlo>
        <GenericMonteCarlo name="pack_fast_mc" preapply="0" trials="2" temperature="0.03"
filter_name="pstat_mc" sample_type="high" mover_name="parsed_pack_fast">
                <Filters>
                    <AND filter_name="total_score_1" temperature="15" sample_type="low"/>
                </Filters>
        </GenericMonteCarlo>
    </MOVERS>
    <PROTOCOLS>
        <Add mover="atomic"/>
        Add mover_name="favor_native" />
        <Add mover_name="fixbb_aro"/>
        <Add mover_name="low_res_dock" />
        <Add mover_name="design_interface" />
        Add mover_name="high_res_dock" />
        <Add mover_name="parsed_pack_fast"/>
        <Add mover_name="pack_fast_mc"/>
        <Add mover_name="pack_mc"/>
        <Add mover_name="min_sc_bb"/>
        Add mover_name="low_res_dock" />
        Add mover_name="design_interface" />
        Add mover_name="high_res_dock" />
        <Add mover_name="add_scores" />
        <Add filter_name="pstat"/>
        <Add filter_name="net_charge"/>
        <Add filter_name="ligen"/>
        <Add filter_name="total_score"/>
        <Add filter_name="bu"/>
    </PROTOCOLS>
    <OUTPUT scorefxn="ref15_1"/>
</ROSETTASCRIPTS>
```

bioRxiv preprint doi: https://doi.org/10.1101/2025.01.30.635804; this version posted January 31, 2025. The copyright holder for this preprint (which was not certified by peer review) is the author/funder, who has granted bioRxiv a license to display the preprint in perpetuity. It is made available under aCC-BY 4.0 International license.

Computational design of FABLE_Flag(.txt)

-out:path:pdb output
-out:path:score output
-extra_res_fa cou485.params
-packing:multi_cool_annealer 10
-packing:linmem_ig 10
-ignore_zero_occupancy false
-s able128_cou485.pdb

Computational design of FABLE_Command line inputs

~/rosetta_bin_linux_2020.08.61146_bundle/main/source/bin/rosetta_scripts.static.linuxgccrelease -database /wynton/home/degradolab/lonelu/rosetta_bin_linux_2020.08.61146_bundle/main/database/ -parser:protocol rscript_dock_fixbb_design_round1.xml @options_helix_1.txt -run:constant_seed -run:jran $SGE_TASK_ID -out:suffix _$SGE_TASK_ID -out:no_nstruct_label

Computational design of FABLE_constraints (.cst)

AtomPair O2 1X ND1 49A HARMONIC 3.0 0.2
AtomPair C8 1X ND1 49A HARMONIC 3.7 0.2
Angle ND1 49A O2 1X C10 1X HARMONIC 1.91 0.12
Dihedral ND1 49A O2 1X C10 1X O1 1X CIRCULARHARMONIC 3.14 0.17

Computational design of FABLE_net charge (.charge)

DESIRED_CHARGE -5 #Desired net charge is zero.
PENALTIES_CHARGE_RANGE -10 -1 #Penalties are listed in the observed net charge range of -2 to +4.
PENALTIES 10 0 0 0 0 0 0 0 0 10 #The penalties are 10 for an observed charge of -2, 0 for an observed charge of -1 to +3, and 10 for an observed charge of +4.
BEFORE_FUNCTION QUADRATIC #Ramp quadratically for observed net charges of -3 or less.
AFTER_FUNCTION QUADRATIC #Ramp quadratically for observed net charges of +5 or greater.

bioRxiv preprint doi: https://doi.org/10.1101/2025.01.30.635804; this version posted January 31, 2025. The copyright holder for this preprint (which was not certified by peer review) is the author/funder, who has granted bioRxiv a license to display the preprint in perpetuity. It is made available under aCC-BY 4.0 International license.

Computational design of FABLE_resfile (.txt)

```
NATRO
ALLAAxc
NOTAA MTC
USE_INPUT_SC
start

1 A PIKAA S
2 A PIKAA TGIFYALSWVHM
3 A PIKAA K
4 A PIKAA S
5 A PIKAA E
6 A PIKAA TGIFYALSWVHM
7 A PIKAA A
8 A PIKAA E
9 A PIKAA TGIFYALSWVMH
10 A PIKAA KVSNLFRTDYAQWEIGHM
11 A PIKAA A
12 A PIKAA V
13 A PIKAA TGIFYALSWVH
14 A PIKAA KVSNLFRTDYAQWEIGHM
15 A PIKAA E
16 A PIKAA TGIFYALSWVNQH
17 A PIKAA KVSNLFRTDYAQWEIGHM
18 A PIKAA A
19 A PIKAA V
20 A PIKAA TGIFYALSWVHM
21 A PIKAA KVSNLFRTDYAQWEIGHM
22 A PIKAA T
23 A PIKAA TGIFYALSWVMH
24 A PIKAA KVSNLFRTDYAQWEIGHM
25 A PIKAA A
26 A PIKAA A
27 A PIKAA TGIFYALSWVHM
28 A PIKAA Q
29 A PIKAA N
30 A PIKAA G
31 A PIKAA D
32 A PIKAA K
33 A PIKAA E
34 A PIKAA A
35 A PIKAA TGIFYALSWVHM
36 A PIKAA A
37 A PIKAA Q
38 A PIKAA Y
39 A PIKAA TGIFYALSWVMH
40 A PIKAA A
41 A PIKAA R
42 A PIKAA TGIFYALSWVMNQH
43 A PIKAA A
44 A PIKAA S
45 A PIKAA L
46 A NATRO
47 A PIKAA T
48 A PIKAA R
49 A NATRO
```

bioRxiv preprint doi: https://doi.org/10.1101/2025.01.30.635804; this version posted January 31, 2025. The copyright holder for this preprint (which was not certified by peer review) is the author/funder, who has granted bioRxiv a license to display the preprint in perpetuity. It is made available under aCC-BY 4.0 International license.

50 A PIKAA E
51 A PIKAA E
52 A PIKAA L
53 A PIKAA TGIFYALSWVHM
54 A PIKAA N
55 A PIKAA R
56 A PIKAA TGIFYALSWVHM
57 A PIKAA L
58 A PIKAA Q
59 A PIKAA K
60 A PIKAA TGIFYALSWVHM
61 A PIKAA R
62 A PIKAA R
63 A PIKAA E
64 A PIKAA G
65 A PIKAA N
66 A PIKAA K
67 A PIKAA E
68 A PIKAA TGIFYALSWVHM
69 A PIKAA V
70 A PIKAA T
71 A PIKAA L
72 A PIKAA TGIFYALSWVHM
73 A PIKAA N
74 A PIKAA E
75 A PIKAA TGIFYALSWVHM
76 A PIKAA T
77 A PIKAA A
78 A PIKAA KVSNLFRTDYAQWEIGHM
79 A PIKAA TGIFYALSWVHM
80 A PIKAA Q
81 A PIKAA T
82 A PIKAA G
83 A PIKAA K
84 A PIKAA S
85 A PIKAA KVSNLFRTDYAQWEIGHM
86 A PIKAA TGIFYALSWVNQHM
87 A PIKAA N
88 A PIKAA A
89 A PIKAA TGIFYALSWVMH
90 A PIKAA KVSNLFRTDYAQWEIGHM
91 A PIKAA A
92 A PIKAA A
93 A PIKAA TGIFYALSWVMH
94 A PIKAA K
95 A PIKAA N
96 A PIKAA G
97 A PIKAA D
98 A PIKAA D
99 A PIKAA D
100 A PIKAA S
101 A PIKAA TGIFYALSWVMH
102 A PIKAA E
103 A PIKAA S
104 A PIKAA KVSNLFRTDYAQWEIGHM
105 A PIKAA VSNLFTYAQWIGHM

bioRxiv preprint doi: https://doi.org/10.1101/2025.01.30.635804; this version posted January 31, 2025. The copyright holder for this preprint (which was not certified by peer review) is the author/funder, who has granted bioRxiv a license to display the preprint in perpetuity. It is made available under aCC-BY 4.0 International license.

106 A PIKAA Q
107 A PIKAA A
108 A PIKAA TGIFYALSWVNQHM
109 A PIKAA KVSNLFRTDYAQWEIGHM
110 A PIKAA K
111 A PIKAA KVSNLFRTDYAQWEIGHM
112 A PIKAA VSNLFTYAQWIGHM
113 A PIKAA A
114 A PIKAA K
115 A PIKAA KVSNLFRTDYAQWEIGHM
116 A PIKAA KVSNLFRTDYAQWEIGHM
117 A PIKAA T
118 A PIKAA KVSNLFRTDYAQWEIGHM
119 A PIKAA TGIFYALSWVHM
120 A PIKAA KVSNLFRTDYAQWEIGHM
121 A PIKAA Q
122 A PIKAA TGIFYALSWVNQHM
123 A PIKAA A
124 A PIKAA K
125 A PIKAA A
126 A PIKAA VSNLFTYAQWIGHM

bioRxiv preprint doi: https://doi.org/10.1101/2025.01.30.635804; this version posted January 31, 2025. The copyright holder for this preprint (which was not certified by peer review) is the author/funder, who has granted bioRxiv a license to display the preprint in perpetuity. It is made available under aCC-BY 4.0 International license.

Computational design of FABLE_cou485 parameters (.params)

```
NAME cou
IO_STRING cou Z
TYPE LIGAND
AA UNK
ATOM  C2  aroC  X   -0.02
ATOM  O1  Oaro  X   -0.57
ATOM  C10 COO   X   0.71
ATOM  O2  OOC   X   -0.67
ATOM  C8  aroC  X   -0.02
ATOM  C4  aroC  X   -0.02
ATOM  C1  aroC  X   -0.02
ATOM  C6  aroC  X   -0.02
ATOM  C7  aroC  X   -0.02
ATOM  C3  aroC  X   -0.02
ATOM  N1  Nhis  X   -0.44
ATOM  C11 CH3   X   -0.18
ATOM  H5  Hapo  X   0.19
ATOM  H6  Hapo  X   0.19
ATOM  H7  Hapo  X   0.19
ATOM  C12 CH3   X   -0.18
ATOM  H8  Hapo  X   0.19
ATOM  H9  Hapo  X   0.19
ATOM  H10 Hapo  X   0.19
ATOM  C5  aroC  X   -0.02
ATOM  H1  Haro  X   0.21
ATOM  H3  Haro  X   0.21
ATOM  H2  Haro  X   0.21
ATOM  C9  CH1   X   0.00
ATOM  F1  F     X   -0.16
ATOM  F2  F     X   -0.16
ATOM  F3  F     X   -0.16
ATOM  H4  Haro  X   0.21
BOND_TYPE  F1   C9  1
BOND_TYPE  F2   C9  1
BOND_TYPE  F3   C9  1
BOND_TYPE  O1   C2  1
BOND_TYPE  O1   C10 1
BOND_TYPE  O2   C10 2
BOND_TYPE  N1   C3  1
BOND_TYPE  N1   C11 1
BOND_TYPE  N1   C12 1
BOND_TYPE  C1   C2  2
BOND_TYPE  C1   C4  1
BOND_TYPE  C1   C6  1
BOND_TYPE  C2   C5  1
BOND_TYPE  C3   C5  2
BOND_TYPE  C3   C7  1
BOND_TYPE  C4   C8  2
BOND_TYPE  C4   C9  1
BOND_TYPE  C5   H1  1
BOND_TYPE  C6   C7  2
BOND_TYPE  C6   H2  1
BOND_TYPE  C7   H3  1
BOND_TYPE  C8   C10 1
BOND_TYPE  C8   H4  1
```

bioRxiv preprint doi: https://doi.org/10.1101/2025.01.30.635804; this version posted January 31, 2025. The copyright holder for this preprint (which was not certified by peer review) is the author/funder, who has granted bioRxiv a license to display the preprint in perpetuity. It is made available under aCC-BY 4.0 International license.

```
BOND_TYPE  C11  H5  1
BOND_TYPE  C11  H6  1
BOND_TYPE  C11  H7  1
BOND_TYPE  C12  H8  1
BOND_TYPE  C12  H9  1
BOND_TYPE  C12  H10 1
CHI 1  C7   C3   N1   C11
CHI 2  C8   C4   C9   F1
NBR_ATOM  C2
NBR_RADIUS 5.923196
ICOOR_INTERNAL    C2     0.000000    0.000000    0.000000   C2    O1    C10
ICOOR_INTERNAL    O1     0.000000  180.000000    1.409420   C2    O1    C10
ICOOR_INTERNAL    C10    0.000000   58.463932    1.386066   O1    C2    C10
ICOOR_INTERNAL    O2   179.925876   57.937170    1.226078   C10   O1    C2
ICOOR_INTERNAL    C8  -179.925892   63.301065    1.472680   C10   O1    O2
ICOOR_INTERNAL    C4    -0.015728   56.938781    1.340468   C8    C10   O1
ICOOR_INTERNAL    C1     0.014038   61.035345    1.469219   C4    C8    C10
ICOOR_INTERNAL    C6  -179.980373   56.250303    1.401339   C1    C4    C8
ICOOR_INTERNAL    C7   179.987003   59.272543    1.399558   C6    C1    C4
ICOOR_INTERNAL    C3    -0.002786   59.626591    1.393812   C7    C6    C1
ICOOR_INTERNAL    N1  -179.998592   59.914843    1.409634   C3    C7    C6
ICOOR_INTERNAL    C11  -29.995192   59.285655    1.444938   N1    C3    C7
ICOOR_INTERNAL    H5  -160.400020   68.664840    1.095868   C11   N1    C3
ICOOR_INTERNAL    H6   118.861740   68.474544    1.096109   C11   N1    H5
ICOOR_INTERNAL    H7   121.608625   69.215932    1.095223   C11   N1    H6
ICOOR_INTERNAL    C12  179.691253   59.261398    1.444710   N1    C3    C11
ICOOR_INTERNAL    H8    79.879643   69.218588    1.095174   C12   N1    C3
ICOOR_INTERNAL    H9   119.524176   68.641682    1.095983   C12   N1    H8
ICOOR_INTERNAL    H10  118.889069   68.492185    1.095932   C12   N1    H9
ICOOR_INTERNAL    C5   179.996131   60.228042    1.390873   C3    C7    N1
ICOOR_INTERNAL    H1   179.316588   58.204851    1.086138   C5    C3    C7
ICOOR_INTERNAL    H3  -179.255672   61.873585    1.087037   C7    C6    C3
ICOOR_INTERNAL    H2  -179.746324   51.829077    1.075641   C6    C1    C7
ICOOR_INTERNAL    C9   179.986364   58.867821    1.500828   C4    C8    C1
ICOOR_INTERNAL    F1    59.996833   68.979155    1.347636   C9    C4    C8
ICOOR_INTERNAL    F2   120.009390   69.167204    1.347464   C9    C4    F1
ICOOR_INTERNAL    F3   119.943271   69.078969    1.347408   C9    C4    F2
ICOOR_INTERNAL    H4   179.999796   63.686307    1.085843   C8    C10   C4
```

bioRxiv preprint doi: https://doi.org/10.1101/2025.01.30.635804; this version posted January 31, 2025. The copyright holder for this preprint (which was not certified by peer review) is the author/funder, who has granted bioRxiv a license to display the preprint in perpetuity. It is made available under aCC-BY 4.0 International license.

Computational design of FABLE_composition (.comp)

```
TYPE THR
DELTA_START 0
DELTA_END 1
PENALTIES 0 100
ABSOLUTE 5
BEFORE_FUNCTION CONSTANT
AFTER_FUNCTION QUADRATIC
END_PENALTY_DEFINITION

PENALTY_DEFINITION
TYPE GLY
DELTA_START 0
DELTA_END 1
PENALTIES 0 100
ABSOLUTE 9
BEFORE_FUNCTION CONSTANT
AFTER_FUNCTION QUADRATIC
END_PENALTY_DEFINITION

PENALTY_DEFINITION
TYPE SER
DELTA_START 0
DELTA_END 1
PENALTIES 0 100
ABSOLUTE 5
BEFORE_FUNCTION CONSTANT
AFTER_FUNCTION QUADRATIC
END_PENALTY_DEFINITION

PENALTY_DEFINITION
TYPE ASN
DELTA_START 0
DELTA_END 1
PENALTIES 0 100
ABSOLUTE 8
BEFORE_FUNCTION CONSTANT
AFTER_FUNCTION QUADRATIC
END_PENALTY_DEFINITION

PENALTY_DEFINITION
TYPE ARG
DELTA_START 0
DELTA_END 1
PENALTIES 0 100
ABSOLUTE 8
BEFORE_FUNCTION CONSTANT
AFTER_FUNCTION QUADRATIC
END_PENALTY_DEFINITION

PENALTY_DEFINITION
TYPE ALA
DELTA_START 0
DELTA_END 1
PENALTIES 0 100
ABSOLUTE 18
```

bioRxiv preprint doi: https://doi.org/10.1101/2025.01.30.635804; this version posted January 31, 2025. The copyright holder for this preprint (which was not certified by peer review) is the author/funder, who has granted bioRxiv a license to display the preprint in perpetuity. It is made available under aCC-BY 4.0 International license.

BEFORE_FUNCTION CONSTANT
AFTER_FUNCTION QUADRATIC
END_PENALTY_DEFINITION

PENALTY_DEFINITION
TYPE TRP
DELTA_START 0
DELTA_END 1
PENALTIES 0 100
ABSOLUTE 3
BEFORE_FUNCTION CONSTANT
AFTER_FUNCTION QUADRATIC
END_PENALTY_DEFINITION

PENALTY_DEFINITION
TYPE LYS
DELTA_START 0
DELTA_END 1
PENALTIES 0 100
ABSOLUTE 8
BEFORE_FUNCTION CONSTANT
AFTER_FUNCTION QUADRATIC
END_PENALTY_DEFINITION

PENALTY_DEFINITION
TYPE GLN
DELTA_START 0
DELTA_END 1
PENALTIES 0 100
ABSOLUTE 8
BEFORE_FUNCTION CONSTANT
AFTER_FUNCTION QUADRATIC
END_PENALTY_DEFINITION

PENALTY_DEFINITION
TYPE TYR
DELTA_START 0
DELTA_END 1
PENALTIES 0 100
ABSOLUTE 8
BEFORE_FUNCTION CONSTANT
AFTER_FUNCTION QUADRATIC
END_PENALTY_DEFINITION

PENALTY_DEFINITION
TYPE GLU
DELTA_START 0
DELTA_END 1
PENALTIES 0 100
ABSOLUTE 8
BEFORE_FUNCTION CONSTANT
AFTER_FUNCTION QUADRATIC
END_PENALTY_DEFINITION

PENALTY_DEFINITION
TYPE ASP

bioRxiv preprint doi: https://doi.org/10.1101/2025.01.30.635804; this version posted January 31, 2025. The copyright holder for this preprint (which was not certified by peer review) is the author/funder, who has granted bioRxiv a license to display the preprint in perpetuity. It is made available under aCC-BY 4.0 International license.

```
DELTA_START 0
DELTA_END 1
PENALTIES 0 100
ABSOLUTE 9
BEFORE_FUNCTION CONSTANT
AFTER_FUNCTION QUADRATIC
END_PENALTY_DEFINITION

PENALTY_DEFINITION
TYPE VAL
DELTA_START 0
DELTA_END 1
PENALTIES 0 100
ABSOLUTE 12
BEFORE_FUNCTION CONSTANT
AFTER_FUNCTION QUADRATIC
END_PENALTY_DEFINITION

PENALTY_DEFINITION
TYPE MET
DELTA_START 0
DELTA_END 1
PENALTIES 0 100
ABSOLUTE 8
BEFORE_FUNCTION CONSTANT
AFTER_FUNCTION QUADRATIC
END_PENALTY_DEFINITION
```

bioRxiv preprint doi: https://doi.org/10.1101/2025.01.30.635804; this version posted January 31, 2025. The copyright holder for this preprint (which was not certified by peer review) is the author/funder, who has granted bioRxiv a license to display the preprint in perpetuity. It is made available under aCC-BY 4.0 International license.

KABLE_Design_Strategy_1_ Constraints

```
AtomPair N3 1X OE2 108A HARMONIC 2.5 0.1
Dihedral N1 1X N2 1X N3 1X OE2 108A CIRCULARHARMONIC 3.14 0.17
Angle OE2 108A N3 1X N2 1X HARMONIC 1.92 0.17
Angle CD 108A OE2 108A N3 1X HARMONIC 1.92 0.17
```

KABLE_Design_Strategy_2_Constraints

```
AtomPair N3 1X OD2 49A HARMONIC 2.5 0.1
Dihedral N1 1X N2 1X N3 1X OD2 49A CIRCULARHARMONIC 3.14 0.17
Dihedral CB 49A CG 49A OD2 49A N3 1X CIRCULARHARMONIC 3.14 0.17
Angle OD2 49A N3 1X N2 1X HARMONIC 1.92 0.17
Angle CG 49A OD2 49A N3 1X HARMONIC 1.92 0.17
```

Example of KABLE_sequence_design_LigandMPNN

```
/home/degradolab/yuda/LigandMPNN/run.py \
        --model_type "ligand_mpnn" \
        --seed 111 \
        --pdb_path "… " \
        --out_folder "…" \
        --fixed_residues "A49" \
        --omit_AA "C" \
        --batch_size 2 \
        --number_of_batches 5 \
```

Example of KABLE_sequence_design_Rosetta FastRelax

```
~/rosetta_bin_linux_2020.08.61146_bundle/main/source/bin/relax.static.linuxgccrelease \
    -database ~ /rosetta_bin_linux_2020.08.61146_bundle/main/database/ \
    -in:file:s $pdb_filename \
    -extra_res_fa ~/…/ 6nt.params \
    -constraints:cst_fa_file ~/…/constraints.cst \
        -constraints:cst_fa_weight "5" \
    -relax:quick \
```

bioRxiv preprint doi: https://doi.org/10.1101/2025.01.30.635804; this version posted January 31, 2025. The copyright holder for this preprint (which was not certified by peer review) is the author/funder, who has granted bioRxiv a license to display the preprint in perpetuity. It is made available under aCC-BY 4.0 International license.

Extract_Chi1_and_Chi2.py

```
import os
import csv
from Bio.PDB import PDBParser
from Bio.PDB.vectors import calc_dihedral
import math

# Define a mapping of residue types to the atoms involved in chi1 and chi2 calculations
residue_atoms = {
    'default': [('N', 'CA', 'CB', 'CG'), ('CA', 'CB', 'CG', 'CD')],  # Default for most amino acids
    'TYR': [('N', 'CA', 'CB', 'CG'), ('CA', 'CB', 'CG', 'CD1')],  # Tyrosine
    'HIS': [('N', 'CA', 'CB', 'CG'), ('CA', 'CB', 'CG', 'ND1')],  # Histidine
    'ASP': [('N', 'CA', 'CB', 'CG'), ('CA', 'CB', 'CG', 'OD1')],  # Aspartic acid
    'GLN': [('N', 'CA', 'CB', 'CG'), ('CA', 'CB', 'CG', 'CD')],   # Glutamine
    'LEU': [('N', 'CA', 'CB', 'CG'), ('CA', 'CB', 'CG', 'CD1')],  # Leucine
    'ILE': [('N', 'CA', 'CB', 'CG1'), ('CA', 'CB', 'CG1', 'CD1')],  # Isoleucine
    'PHE': [('N', 'CA', 'CB', 'CG'), ('CA', 'CB', 'CG', 'CD1')],  # Phenylalanine
    'THR': [('N', 'CA', 'CB', 'OG1')],  # Threonine (chi1 only)
    # Add more residue types as needed
}

def calculate_dihedrals(pdb_file, residue_number, residue_type='default'):
    parser = PDBParser()
    structure = parser.get_structure('X', pdb_file)
    atom_pairs = residue_atoms.get(residue_type, residue_atoms['default'])
    chi1_atoms = atom_pairs[0]  # Chi1 is always the first set of atoms
    chi2_atoms = atom_pairs[1] if len(atom_pairs) > 1 else None  # Chi2 is optional
    for model in structure:
        for chain in model:
            for residue in chain:
                if residue.id[1] == residue_number:
                    try:
                        # Extract vectors for chi1 calculation
                        vectors_chi1 = [residue[x].get_vector() for x in chi1_atoms]
                        chi1 = calc_dihedral(*vectors_chi1)
                        chi1_deg = math.degrees(chi1)

                        # Extract vectors for chi2 calculation if available
                        chi2_deg = None
                        if chi2_atoms:
                            vectors_chi2 = [residue[x].get_vector() for x in chi2_atoms]
                            chi2 = calc_dihedral(*vectors_chi2)
                            chi2_deg = math.degrees(chi2)
                        return chi1_deg, chi2_deg
                    except KeyError:
                        # Atoms required not found
                        return None, None
    return None, None  # Residue not found

def process_directory(directory_path, residue_number, residue_type, output_csv):
    with open(output_csv, 'w', newline='') as csvfile:
        fieldnames = ['File Name', 'Chi1 (degrees)', 'Chi2 (degrees)']
        writer = csv.DictWriter(csvfile, fieldnames=fieldnames)
        writer.writeheader()

        for file in os.listdir(directory_path):
```

bioRxiv preprint doi: https://doi.org/10.1101/2025.01.30.635804; this version posted January 31, 2025. The copyright holder for this preprint (which was not certified by peer review) is the author/funder, who has granted bioRxiv a license to display the preprint in perpetuity. It is made available under aCC-BY 4.0 International license.

```
        if file.endswith(".pdb"):
            pdb_file_path = os.path.join(directory_path, file)
            chi1, chi2 = calculate_dihedrals(pdb_file_path, residue_number, residue_type)
            writer.writerow({'File Name': file, 'Chi1 (degrees)': chi1, 'Chi2 (degrees)': chi2 if chi2 is not None else
"N/A"})

# Example usage
directory_path =
'/Users/chenyuda/Documents/Code/ABLE_Fragment/fragment_paper/md_278k_hie_202404/chi1chi2_for_H49/all_
pdb_for_H49/'
residue_number = 49  # Example residue number
residue_type = 'HIS'  # Specify residue type here
output_csv = 'Chi1Chi2_md_Thr112_6w70_ABLE_6w6x_6w70_and_pdb.csv'
process_directory(directory_path, residue_number, residue_type, output_csv)
```

bioRxiv preprint doi: https://doi.org/10.1101/2025.01.30.635804; this version posted January 31, 2025. The copyright holder for this preprint (which was not certified by peer review) is the author/funder, who has granted bioRxiv a license to display the preprint in perpetuity. It is made available under aCC-BY 4.0 International license.

Generate_contour_from_tyr_chi1and2.py

```
import numpy as np
import pandas as pd
import matplotlib.pyplot as plt
from scipy.stats import gaussian_kde
from matplotlib.colors import LinearSegmentedColormap

def plot_density_contour(file_path, labels, x_range, y_range, contour_color, scatter_colors, contour_line_color,
bg_color='white', contour_alpha=0.6, contour_linewidth=3, symbol_sizes=None, output_file='density_contour.svg'):
    fig, ax = plt.subplots(figsize=(10, 8), facecolor=bg_color)
    ax.set_facecolor(bg_color)

    # Read the CSV file
    data_frame = pd.read_csv(file_path, header=None)

    # Extract group names
    group_names = data_frame.iloc[0, ::2].values

    # Process contour group (first group)
    chi1_contour = data_frame.iloc[1:, 0].dropna().values.astype(float)
    chi2_contour = data_frame.iloc[1:, 1].dropna().values.astype(float)
    data_contour = np.vstack([chi1_contour, chi2_contour])
    kde_contour = gaussian_kde(data_contour)
    x_min, x_max = x_range
    y_min, y_max = y_range
    x_grid, y_grid = np.mgrid[x_min:x_max:100j, y_min:y_max:100j]
    grid_coords = np.vstack([x_grid.ravel(), y_grid.ravel()])
    density_contour = kde_contour(grid_coords).reshape(x_grid.shape)
    cmap = LinearSegmentedColormap.from_list(f'custom_cmap_{labels[0]}', ['white', contour_color])
    contour_filled = ax.contourf(x_grid, y_grid, density_contour, levels=4, cmap=cmap, alpha=contour_alpha,
extend='both')
    contour_lines = ax.contour(x_grid, y_grid, density_contour, levels=4, colors=contour_line_color,
linewidths=contour_linewidth)
    ax.scatter(chi1_contour, chi2_contour, c=contour_color, s=symbol_sizes[0], label=labels[0])
    print(f"{labels[0]}: {len(chi1_contour)} points")

    # Process scatter groups (all other groups)
    for i in range(1, len(labels)):
        chi1_scatter = data_frame.iloc[1:, 2*i].dropna().values.astype(float)
        chi2_scatter = data_frame.iloc[1:, 2*i+1].dropna().values.astype(float)

        if len(chi1_scatter) == 0 or len(chi2_scatter) == 0:
            print(f"{labels[i]}: No data found")
            continue

        ax.scatter(chi1_scatter, chi2_scatter, c=scatter_colors[i-1], s=symbol_sizes[i], label=labels[i])
        print(f"{labels[i]}: {len(chi1_scatter)} points")

    # Add color bar for the filled contour
    plt.colorbar(contour_filled, ax=ax, label='Density')

    # Customizing the range for the x and y axes
    ax.set_xlim([x_min, x_max])
    ax.set_ylim([y_min, y_max])

    ax.set_xlabel('chi1')
```

bioRxiv preprint doi: https://doi.org/10.1101/2025.01.30.635804; this version posted January 31, 2025. The copyright holder for this preprint (which was not certified by peer review) is the author/funder, who has granted bioRxiv a license to display the preprint in perpetuity. It is made available under aCC-BY 4.0 International license.

```
    ax.set_ylabel('chi2')
    ax.set_title('Density Contour Plot of chi1 and chi2')
    ax.legend()

    # Save the plot as an SVG file
    plt.savefig(output_file, format='svg')
    print(f"Plot saved as {output_file}")

    plt.show()

# File path to the CSV file
file_path = '…' # Replace with your actual file path

# Labels for the datasets
labels = ['complexed ABLE MD Simulation', 'Uncomplexed ABLE', 'Uncomplexed ABLE H49A', 'ABLE-apixaban complex']

# Colors for the contour plot
contour_color = '#F39191'

# Colors for the scatter plots
scatter_colors = ['#1873E5', '#59B358', '#5E4B56']

# Color for the contour lines
contour_line_color = '#ffc8c8'

# Custom ranges for x and y axes
x_range = [225, 340]
y_range = [-125, 50]

# Symbol sizes for the datasets (contour and five scatter plots)
symbol_sizes = [20, 80, 80, 80]

# Call the function with desired customizations and specify output file
output_file = '…'
plot_density_contour(file_path, labels, x_range, y_range, contour_color, scatter_colors, contour_line_color, bg_color='white', contour_alpha=0.8, contour_linewidth=0.1, symbol_sizes=symbol_sizes, output_file=output_file)
```

bioRxiv preprint doi: https://doi.org/10.1101/2025.01.30.635804; this version posted January 31, 2025. The copyright holder for this preprint (which was not certified by peer review) is the author/funder, who has granted bioRxiv a license to display the preprint in perpetuity. It is made available under aCC-BY 4.0 International license.

Count_water.py.

```
import prody as pr
import matplotlib.pyplot as plt
import csv
#import warnings
#warnings.filterwarnings('ignore')

####### Script settings ######
discard_frames = […]

lig_dist = 3.5

traj_pdb_path = '…'

lig_chain = '…'

lig_resnum = 127

output_csv_file = '…'

######## Functions #######

def main():
    # Load MD trajectory
    traj_pdb = pr.parsePDB(traj_pdb_path)
    print('-'*20, 'Total number of frames in traj: ', len(traj_pdb.getCoordsets()), '-'*20)

    # Perform analysis
    num_waters_per_frame = [] # Each element in this list represents a single frame.
    num_frames_processed = 0
    # Iterate over each frame (model), which is a Coordset in prody
    for frame_ix, coordset in enumerate(traj_pdb.getCoordsets()):
        if frame_ix in discard_frames:
            continue
        num_frames_processed += 1
        # Make a standalone prody obj for each frame
        par = pr.atomic.atomgroup.AtomGroup()
        par.setCoords(coordset)
        par.setNames(traj_pdb.getNames())
        par.setResnames(traj_pdb.getResnames())
        par.setChids(traj_pdb.getChids())
        par.setResnums(traj_pdb.getResnums())
        par.setElements(traj_pdb.getElements())

        # Define ligand.
        if lig_chain:
            pr_lig = par.select(f'chain {lig_chain} and resnum {lig_resnum}')
        else:
            pr_lig = par.select(f'resnum {lig_resnum}')

        lig_resname = set(pr_lig.getResnames())
        lig_heavyatoms = pr_lig.select('not element H')
        if len(lig_resname) > 1:
            print(f'Note: more than one ligand selected. Residue names {lig_resname}.')
```

bioRxiv preprint doi: https://doi.org/10.1101/2025.01.30.635804; this version posted January 31, 2025. The copyright holder for this preprint (which was not certified by peer review) is the author/funder, who has granted bioRxiv a license to display the preprint in perpetuity. It is made available under aCC-BY 4.0 International license.

```
        if len(num_waters_per_frame) == 0:
            print(f'Ligand {lig_resname} selected with {len(lig_heavyatoms)} heavy atoms.')

        # Count waters in the active site, defined by a distance cutoff from the ligand.
        wat_neighbs = par.select(
                f'(water and name O) within {lig_dist} of Lig', Lig=pr_lig)
        if wat_neighbs is None:
            num_waters_in_frame = 0
        else:
            num_waters_in_frame = len(wat_neighbs)

        # Record the frame number (1-indexed) and the number of waters in that frame.
        num_waters_per_frame.append(tuple([frame_ix+1, num_waters_in_frame]))

    framenumbers = [i[0] for i in num_waters_per_frame]
    numberwaters = [i[1] for i in num_waters_per_frame]

    # Write out the results to a csv file.
    with open(output_csv_file, 'w', newline='') as csvfile:
        writer = csv.writer(csvfile)
        writer.writerow(['Frame #', '# waters'])
        for frame, waters in zip(framenumbers, numberwaters):
            writer.writerow([frame, waters])

    # Plot
    plt.plot(framenumbers, numberwaters)
    plt.xlabel('Frame #')
    plt.ylabel('# waters in active site')
    plt.title(traj_pdb_path)
    plt.grid(True)
    plt.show()

    print(f'Number of frames processed: {num_frames_processed}')
    print(f'Number of frames discarded: {len(discard_frames)}')



######## Main #######
if __name__ == "__main__":
    main()
```
